# Anion-Facilitated Hydrogen–Deuterium Exchange as a Tool to Probe Weak Anion–Protein Interactions Responsible for Hofmeister Effects

**DOI:** 10.1021/acs.jpcb.4c08619

**Published:** 2025-02-13

**Authors:** Thien H. Tran, Meghan Ricciardi, Lilly I. Grunski, William C. Wimley, Marcey L. Waters, Bruce C. Gibb

**Affiliations:** † Department of Chemistry, Tulane University School of Science and Engineering, New Orleans, Louisiana 70118, United States; ‡ Department of Chemistry, University of North Carolina at Chapel Hill, Chapel Hill, North Carolina 27599, United States; § Department of Biochemistry and Molecular Biology, Tulane University School of Medicine, New Orleans, Louisiana 70112, United States

## Abstract

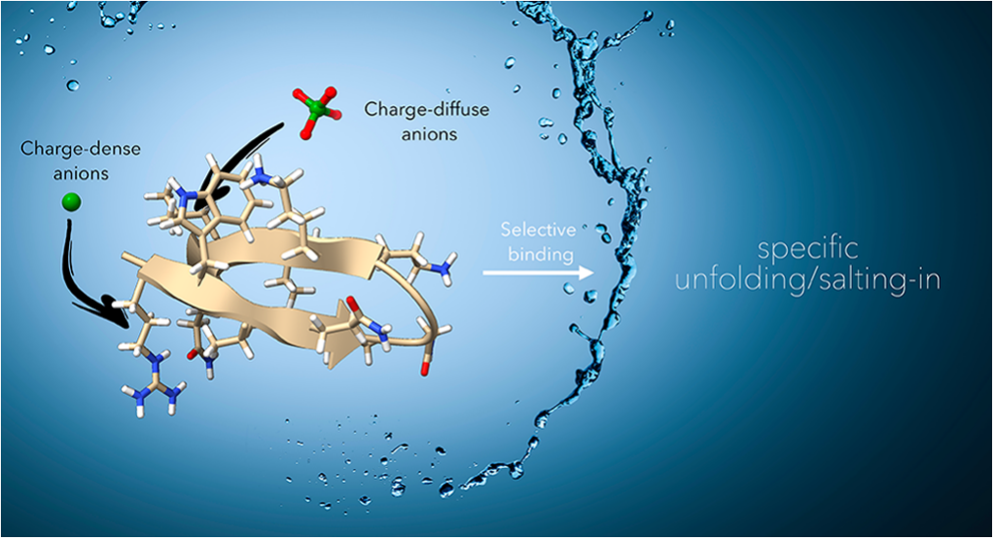

Impeded by the complexity of proteinaceous structure and the very weak nature of the noncovalent interactions involved, the detailed mechanisms by which anions induce salting-in Hofmeister effects in proteins and peptides remain unclear. Here, using β-hairpin peptides as models, we examine two approaches to qualify (map) anion binding: ^1^H NMR chemical shifts and hydronium-catalyzed hydrogen–deuterium exchange (HDX) rate changes. We demonstrate that each salt investigated—despite an affinity too weak to quantify accurately, caused denaturation to an extent that is both peptide and anion-specific, with more charge-diffuse anions inducing a greater degree of unfolding. Our studies reveal that the HDX mapping provides more detail than chemical shift data. Thus, HDX mapping reveals two slightly different mechanisms of denaturation, depending on the nature of the anion. Namely, assisted by a N-terminal Arg residue, charge-dense Cl^–^ is chelated by the terminal N–H groups of the hairpin and induces a small degree of denaturation, whereas charge-diffuse anions intercalate deeply into the cation-π-hydrophobic core of the peptide and induce more substantial unfolding. These findings provide a glimpse of the different mechanisms by which anions can induce the salting-in Hofmeister effect in peptides and proteins and suggest HDX as a useful tool to map weak anion binding.

## Introduction

The ability of salts to alter the physiochemical properties of aqueous solutions has been recognized since the 1880s.^1−4^ For biomolecules, these Hofmeister effects can occur through two distinct mechanisms. First, strong water–salt interactions and the attendant water sequestration can indirectly affect the properties of other solutes dissolved in solution. Alternatively, weak water–salt interactions can allow salt ions to interact directly with and affect the properties of a solute. By this second route, direct salt–protein interactions can increase or decrease the solubility of a protein. Thus, if an anion binds to a positively charged protein and so reduces its net charge, complexation can induce precipitation.^5−12^ Alternatively, if ion-binding results in an increase in net charge, then the solubility of a protein or peptide can be increased. Whether ion–protein interactions lead to salting-out—commonly called the inverse or reverse Hofmeister effect, or salting-in depends not only on the pI of the protein and the pH of the solution but also whether ion-binding induces unfolding. Unfortunately, a structurally detailed understanding of ion-induced unfolding is lacking.

Complicating an already complex situation, there are multiple types of noncovalent interactions (NCIs) possible between ions and proteins. Anions typically induce larger Hofmeister effects than cations,^2−4^ and these involve combinations of three general types of NCIs: (1) Coulombic NCIs with the ammonium, guanidinium, and imidazolium side chains of Lys, Arg, and His residues;^13−17^ (2) hydrogen bonding to both amide N–H and C_α_–H moieties;^18−25^ and (3) van der Waals (VdW) type NCIs between anions and nonpolar regions of a solute.^21,26−43^ The combination of the complexity of protein structure and the range of NCIs they can form with anions results in a complex, multidimensional chemical space.

Understanding how anions bind to proteins and hence advancing our understanding of Hofmeister effects has been slowed by the general weakness of the NCIs involved. This situation is essentially the reverse of what is typical of supramolecular chemistry, where hosts are specifically designed to bind guests as strongly as possible (desirable). How do we gain information about weak interactions that cannot be quantified (a *K*_a_ value) and are typically described as “nonspecific”?

To examine ways to address this issue we have targeted two 12-mer β-hairpins^44,45^ conceived by the Gellman and Waters groups.^46−50^ As discussed below, we demonstrate that these adopt a hairpin structure possessing a Type-1′ turn,^51,52^ and that anion binding to these recipients^53^ induces their denaturation (salting-in). As anticipated, the association of anion to the β-hairpins is demonstrated to be weak. Thus, to probe for specific binding sites and learn more about how they are denatured, we investigated mainchain amide N–H ^1^H NMR chemical shifts and hydrogen–deuterium exchange (HDX) rates as a function of added salt. These studies revealed that although chemical shift data proved structurally informative, HDX experiments provided a more precise map of anion association. Thus, this approach—anion-facilitated HDX (AF-HDX)—reveals that charge-dense anions such as Cl^–^, and charge-diffuse anions such as ClO_4_^–^ or ReO_4_^–^, bind in different ways and so induce the denaturation of the hairpins via slightly different mechanisms. Moreover, these early results suggest that AF-HDX may be a valuable strategy for mapping anion-binding events to proteins too weak to directly quantify.

## Results and Discussion

### Peptide Recipient Selection

**Figure 1:**
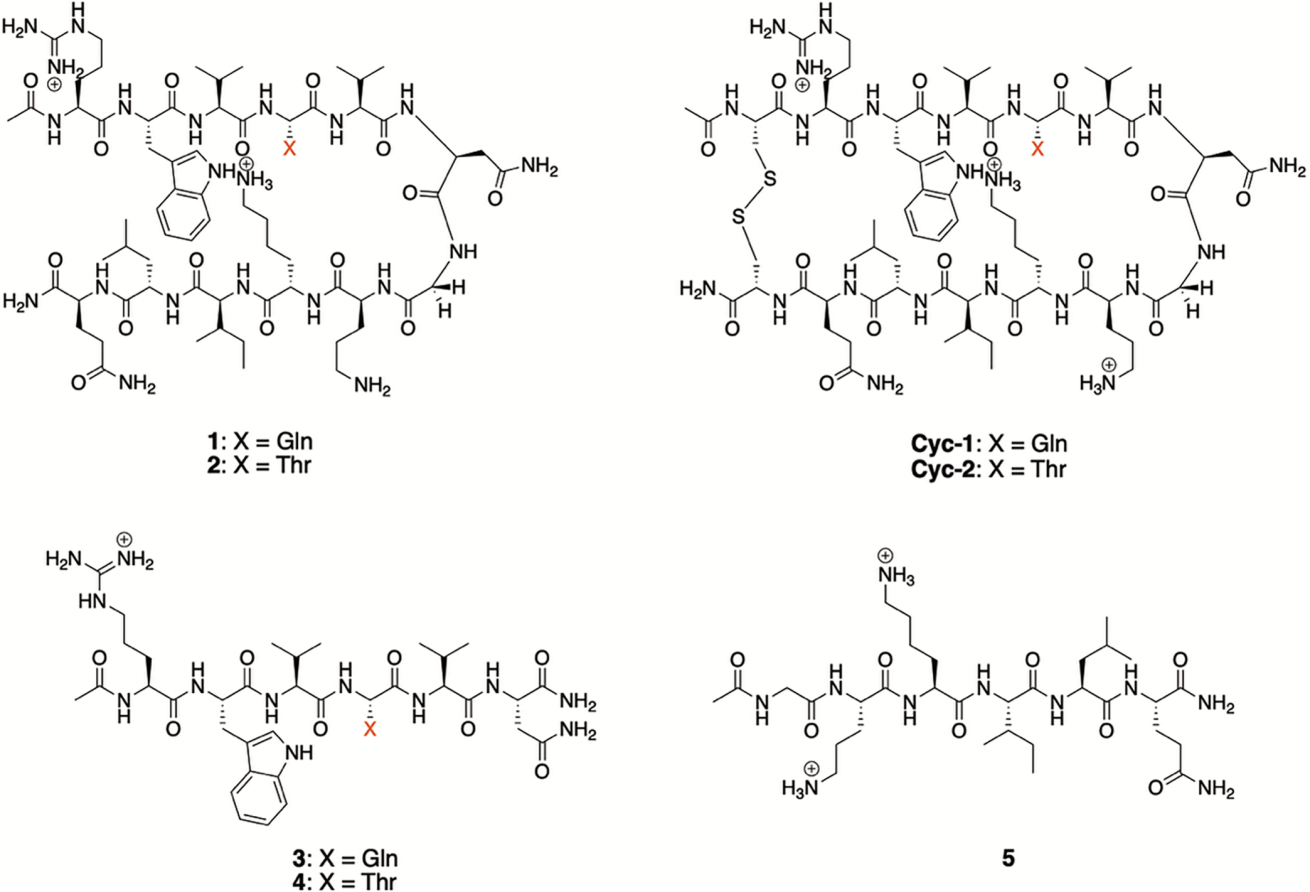
Peptides used in this study.

The structures of the recipient peptides used in this study are shown in Figure 1. The general design of β-sheets **1** and **2** originated in the Gellman group^46−48^ but included two key modifications from the Waters’ group:^49,50^ a Y2W mutation to ensure a strong cation–π interaction with K9, and a P6N mutation to yield a _5_VNGO_8_ Type-1′ turn that was demonstrated by the Searle group to strongly promote β-hairpin formation.^54^ In their studies, the Waters group had identified that an E4 residue also led to a high degree of hairpin structure, but as we were focusing on anion complexation, we targeted uncharged residues possessing reasonable β-strand propensities at this site (i.e., **1** and **2**, X = Q or T). Thus, the general sequence Ac-RWVXVNGOKILQ-NH_2_ is shown (Figure 1). We expected peptides **1** and **2** to adopt β-hairpin structures held together in part by (1) a cation–π interaction between W2 and K9; (2) nonpolar (hydrophobic) interactions between W2 and L11, as well as V3, V5, and I10; and (3) the intramolecular mainchain amide hydrogen bonds.

As discussed below, our studies also required several reference peptides, specifically macrocyclic peptides **Cyc-1** and **Cyc-2**, and single-strand, half-peptides **3**-**5** (Figure 1). The 14-mer cyclic peptides possessed the same main sequence as **1** and **2** but were constrained by a cystine bridge between Cys residues added to the N- and C-termini. We use these macrocyclic peptides as surrogates for the fully folded state. Our studies also required three half-peptides possessing the same sequences as peptides **1** and **2**, but with the addition of capping groups; specifically, a C-terminal −NH_2_ group in **3** and **4**, and an N-terminal −Ac group in peptide **5**. These half peptides are taken as surrogates for the fully unfolded state.^55^ Synthesis details of peptides **1**–**5** are given in Section 1 of the Supporting Information (SI).

### Recipient Structure: Spectroscopic Analysis

^1^H, TOCSY, ROESY, and COSY NMR experiments were used to characterize each peptide at pH = 2.3 (50 mM sodium phosphate buffer). These confirmed the hairpin nature of compounds **1** and **2**. Thus, both peptides possessed common, strong NOEs between: the aromatic protons of W2 and side-chain methylenes of K9, the aromatic protons of W2 and the methyl and methine proton of L11, and the side-chain N–Hs of R1 and the side-chain methylenes of Q12 (for representative data from **1**, see Section 2, SI). These latter NOEs suggest hydrogen bonding not only between the mainchain amide groups of R1 and Q12 but also between the guanidinium of R1 and the side-chain carbonyl of Q12 (vide infra). Peptides **1** and **2** also possessed weak NOEs commensurate with all of the aforementioned interactions. As anticipated, similar sets of NOEs were seen in the cyclic peptides (Section 2, SI). Taken together, these data demonstrate the folding of peptides **1** and **2** into well-defined β-hairpins held together by the aforementioned NCIs.

**Figure 2:**
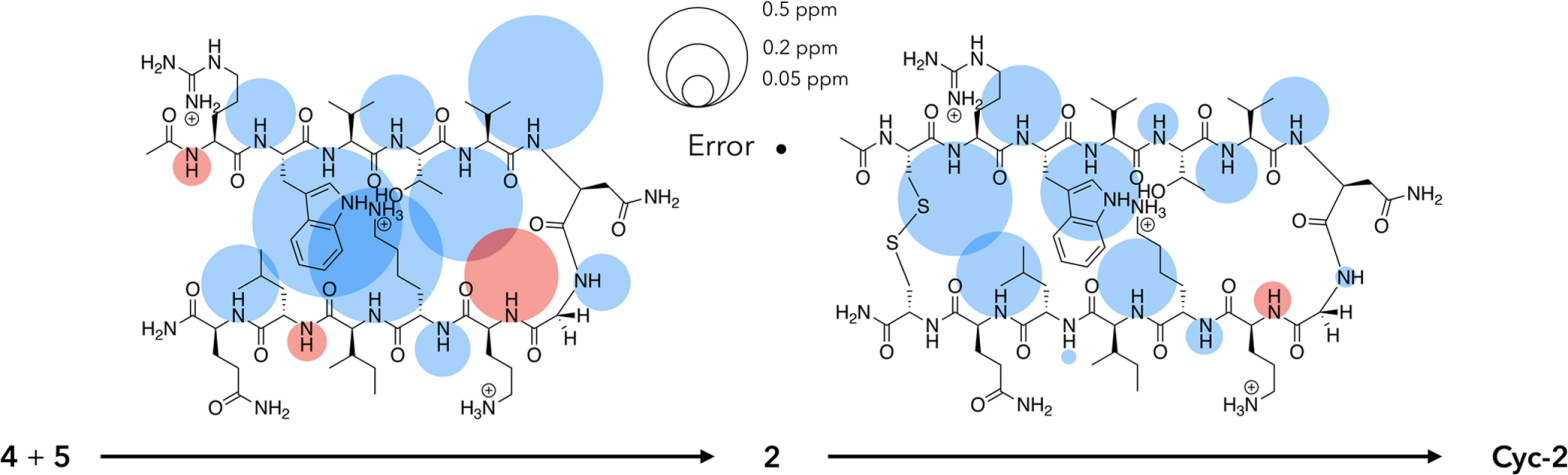
Unreferenced response (Δδ) of amide N–H ^1^H NMR signals upon the linking of half peptides **4** and **5** to generate hairpin **2** (Δδ = (δ_N–H_ values for **2**) – (δ_N–H_ values for **4** or **5**)), and the macrocyclization of **2** by the inclusion of a cystine bridge across the termini to generate **Cyc-2** (Δδ = (δ_N–H_ values for **Cyc-2**) – (δ_N–H_ values for **2**)). In both bubble maps, the areas of the circles associated with each N–H group are proportional to its ^1^H NMR signal shift, with upfield signals shown in red and downfield shifts in blue. Where shifts are small, the circle is shown above or below the amide H atom. A scale and the error for ^1^H NMR signal shifts (±0.005 ppm) are shown in the *top center*.

We sought to both qualify and quantify hairpin formation. Figure 2 shows one approach to qualification using mainchain amide N–H chemical shifts. This shows the ^1^H NMR signal shift for each residue as a shaded circle whose area is proportional to the Δδ (ppm) value observed when performing two sequential thought processes: the joining of the two half-peptides to form peptide **2**, and the joining of the termini of **2** to form **Cyc-2**. Thus, the *left* structure shows the effect of joining **4** and **5** to form **2** (Δδ = (δ_N–H_ values for **2**) – (δ_N–H_ values for **4** or **5**)), while the *right* structure shows the signal shifts associated with macrocyclization (Δδ = (δ_N–H_ values for **Cyc-2**) – (δ_N–H_ values for **2**)). In these Δδ bubble maps, downfield (upfield) shifts are shown in blue (red). The general predominance of downfield shifts is in accord with β-sheet formation,^56^ while the smaller differences between **2** and **Cyc-2** (*right*) versus **4** and **5** and **2** (*left*) suggest that **2** exists primarily in a hairpin conformation, and that inserting the cystine bridge leads to only slight structural enhancement. It is evident from joining the half peptides that the greatest shifts are observed with the inward-pointing N–H groups, particularly those in the core. An exception to this is the outward-pointing N6 mainchain amide that, as anticipated for the *i* + 1 residue in a Type-1′ turn, also undergoes a large downfield shift.^51,52^ The sizable upfield shift in O8 is also in accord with the *i* + 3 residue shift in a Type-1′ turn; typically, this amide N–H cannot adopt a linear hydrogen bond to the V5 carbonyl because of its proximity to the turn. In the “building” of **2**, also of note are the small upfield shifts in the mainchain amides of R1 and L11. The upfield shift of R1 likely reflects φ and ψ angles that deviate substantially from those typical of the β-sheet, commensurate with the frayed nature of termini, as well as weak hydrogen bonding to the Q12 carbonyl. Considering the known affinity of phosphate (buffer) for guanidiniums,^14^ this upfield shift may also arise from buffer anion binding to the side chain of **2**. In contrast, the upfield shift in L11 upon hairpin formation has been attributed to the N–H amide residing in the shielding zone of the indole side chain of W2;^50^ a hypothesis that is supported by molecular modeling work (*vide infra*). Examining the signal shifts when hairpin **2** is converted to **Cyc-2** (Figure 2, *right*), it is evident that the largest signal shifts occur adjacent to the inserted cystine bridge. These significant downfield shifts at R1 and Q12 suggest an enhanced β-sheet structure at the termini. **Cyc-2** also differs from **2** by showing considerably enhanced sheet formation in the core V3 and I10 residues and, to a lesser extent, enhanced Type-1′ turn characteristics at N6 and O8. It is also interesting to note that inserting the cystine bridge has much more of an effect on strand-1 than it does on strand-2; regardless of whether the mainchain amide groups point inward or outward. This suggests that in **2**, strand-2 has more intrinsic sheet-like structure.

To quantify hairpin formation, we followed literature precedent and, treating the hairpin as a two-state model (folded versus unfolded),^57^ first examined the signal splitting of the diastereotopic methylene protons of G7. As has been shown by the Searle group,^58^ the fraction folded can be determined using G7 as a global reporter and eq 1
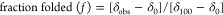
1where δ_obs_ is the observed G7 signal split of the diastereotopic C_α_H protons of the hairpin (in ppm), δ_100_ is the G7 signal (in ppm) of its macrocyclic form, and δ_0_ is the G7 signal of its corresponding 6-mer half peptide. By this approach, β-hairpins **1** and **2** were found to be 52 and 79% folded. Comparing the signal shift data in Figure 2 with the corresponding Δδ, bubble maps for the formation and macrocyclization of β-hairpin **1** concur with these percentage folds. Thus, although the data for **1** (Section 2, SI) show the same general upfield and downfield shift pattern as peptide **2** (Figure 2), the “joining” of the two half peptides (**3** and **5**) to form **1** led to relatively small signal shifts, whereas the joining of the termini of **1** to form **Cyc-1** and **1** led to much larger chemical shifts.

**Figure 3:**
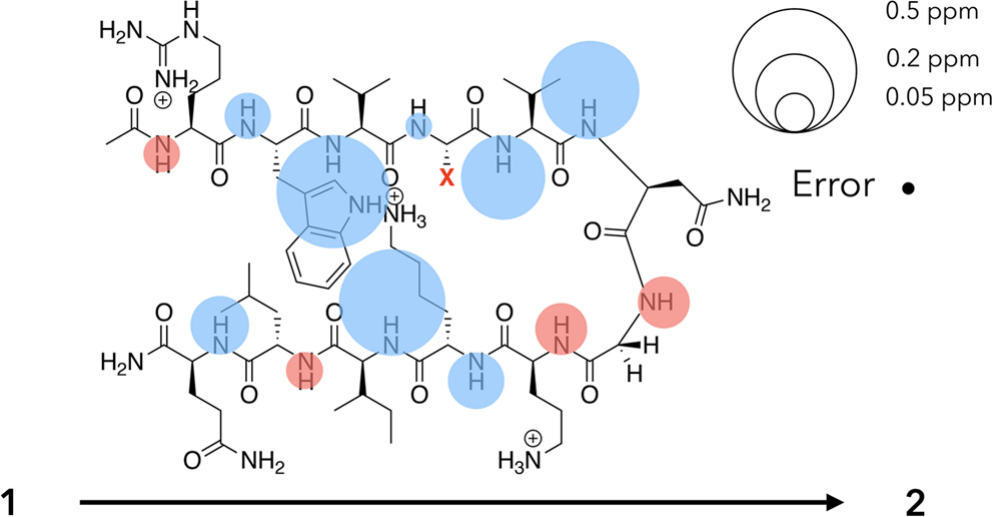
Unreferenced response (Δδ) of amide N–H ^1^H NMR signals upon the Q4T mutation converting **1** to **2** (Δδ = (δ_N–H_ values for **2**) – (δ_N–H_ values for **1**)). The areas of each circle associated with an N–H group are proportional to its ^1^H NMR signal shift, with upfield signals shown in red and downfield shifts shown in blue. Where shifts are small, the circle is shown above or below the amide H atom. A scale and the error-bubble for ^1^H NMR signal shifts (±0.005 ppm) are shown in the *upper right*.

Another way to express the fold differences of **1** and **2** is shown in Figure 3, which qualifies the signal shifts upon the Q4T mutation and the formation of more folded **2**. The pattern of Δδ values is commensurate with the tightening of the structure (cf. Figure 2). The one exception to this is the upfield shift in the G7 amide. As we discuss below, we attribute this change to a through-space effect arising from this mutation.

We also carried out a signal shift study of each C_α_–H proton of peptides **1** and **2**, as well as Circular Dichroism analysis of folding (Section 2 of SI). The data from both approaches supported the formation of β-hairpin structure formation.

### Recipient Structure: Energy-Minimized Conformations

To confirm the findings from ^1^H NMR, we carried out computational calculations. Specifically, we examined the structures of **1**, **Cyc-1**, and **2** using AlphaFold^59^ with ColabFold^60^ interface powered by the Many-against-Many sequence-searching (MMseqs2) cluster. Each resulting structure was then minimized using the Gaussian 16 package and the M06/6-31(d,p)^61^ method to model the key cation-π interaction in each peptide. Full details are given in Section 2.3 of the SI.

The analysis of the three peptides revealed the expected close proximity of the aromatic protons of W2 and side-chain methylenes of K9; the aromatic protons of W2 and the methyl and methine proton of L11; and the side-chain N–Hs of R1 and the side-chain methylenes of Q12.^50^ On this last point, the minimized structures demonstrated not only mainchain amide hydrogen bonds between R1 and Q12 but also—as suggested by the NOE studies—a bifurcated hydrogen bond involving the side-chain guanidinium of R1 and the side-chain carbonyl of Q12.

**Figure 4:**
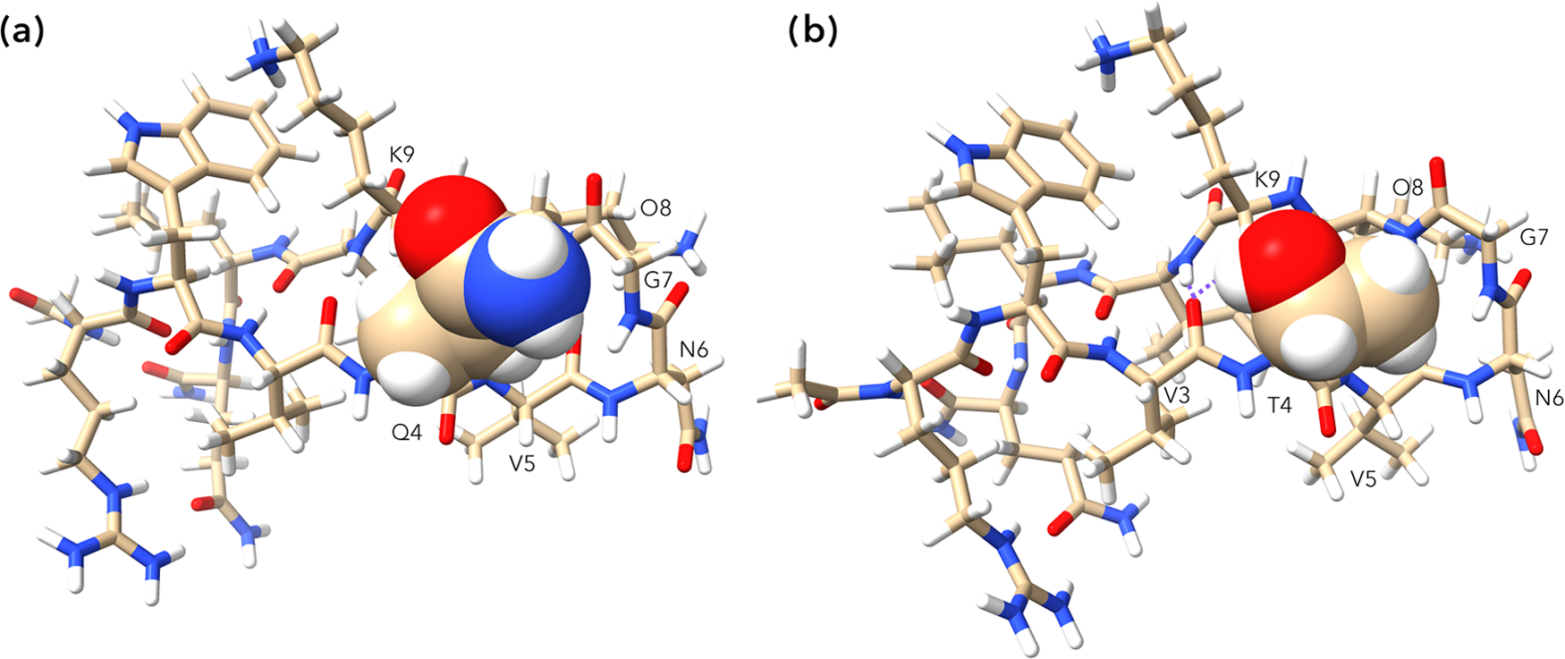
Energy minimized structures of **1** (a) and **2** (b) showing their respective Q4 and T4 side chains in space-filling representation. In the latter, the hydrogen bonds between the V3 carbonyl and the I10 amide N–H and the side-chain O–H of T4 are highlighted with dashed purple lines.

This study also revealed that the turns of **1** and **Cyc-1** were very similar, but that there was a key difference in the turns of **1** and **2**. Thus, in the former, the side-chain amide of Q4 is oriented such that the H_γ_ protons of Q4 are proximal to the amide N–H protons of V5, G7, and O8, and the side-chain carbonyl is oriented to point its dipole at the relatively distant (7.86 Å) K9 ammonium group (Figure 4, *left*). Oriented thus, the side-chain amide −NH_2_ points toward the turn, where it does not pair with any hydrogen bond acceptors. In contrast, in the case of **2**, the T4 side-chain OH forms a hydrogen bond to the carbonyl of V3 (Figure 4, *right*), while simultaneously placing its methyl into the small concavity formed by the turn. These findings were confirmed by NOE data (Section 2, SI) and concur with the upfield shift of the G7 amide N–H in the Q4T mutation (Figure 3). We conclude that the nestling of the methyl group in the concavity of the turn and the formation of the OH···O=C hydrogen bond enhance the overall stability of hairpin **2** relative to **1**.

Finally, returning to the upfield shift of the L11 amide upon joining **4** and **5** to form **2** (Figure 2, *left*), we also examined the proximity of this group to the indole of W2. In the obtained model, this distance was calculated to be only 3.37 Å, suggesting that despite its outward-pointing nature, this residue is a sensitive reporter of changes to the cation-π-hydrophobic core.

### Anion-Induced Recipient Denaturation

We assessed the effects of adding NaCl, NaClO_4_, and NaReO_4_ to solutions of **1** and **2**. In these titration experiments, we again monitored the signal splitting (Δδ) of the G7 methylene as a function of salt. Initially, we ascertained that the effects of adding salts to **Cyc-1** and **Cyc-2** and half-peptides **3**-**5** were negligible. This allowed us to determine the extent of folding of **1** and **2** in the presence of salts (Section 4, SI) at any salt concentration using eq 1. As Table 1 shows, there is a reduction in the %-folding of all hairpins with each anion, with increased charge-diffusivity and higher salt concentration leading to greater denaturation. The data reveal a complex relationship between the nature of the anion and the peptide sequence/structure. For example, despite **2** being more stable than **1**, the addition of ReO_4_^–^ leads to a greater degree of unfolding of the former. Our interpretation of these results is that a combination of slightly different packing within each peptide and the precise nature of the anion leads to different modes of anion binding and hence different mechanisms of anion-induced denaturation. In short, there is specificity.

**Table 1:** Percentage (%) Unfolding of Peptides **1**–**4** Induced by the Addition of Sodium Salts^a^

	48 eq (230 mM) anion		160 eq (650 mM) anion	
	**1**	**2**	**1**	**2**
Cl^–^	1.7	3.4	2.6	5.7
ClO_4_^–^	1.9	5.7	4.4	11.2
ReO_4_^–^	7.5	11.3	14.6	22.0

a For recipients **1** and **2**, the corresponding cyclic and half peptides were used to evaluate changes in folding. The maximum errors were ±0.7%.

### Mapping Anion Binding: ^1^H NMR Chemical Shifts

We sought to qualify anion binding using bubble diagrams to map changes induced by anions. We first turned to chemical shift data, looking for residue-specific information concerning where anions interact with the hairpins to induce the observed denaturation. To assess anion binding, we focused on β-hairpin **1** and macrocycle **Cyc-1**. Studies were carried out at pH = 2.3 (50 mM phosphate buffer) to allow all amide N–H proton signals to report on anion binding. In total, we explored three sodium salts covering a range of charge-diffusivity: Cl^–^, ClO_4_^–^, and ReO_4_^–^ (Section 4, SI). In each titration experiment, the salt concentration ranged from 0 to 650 mM,^62^ and to correct for ionic strength effects, the N-terminal acetyl methyl protons were employed as an internal reference; the behavior of this group was essentially identical to that of external standards such as 3-(trimethylsilyl)propane-1-sulfonate.

**Figure 5:**
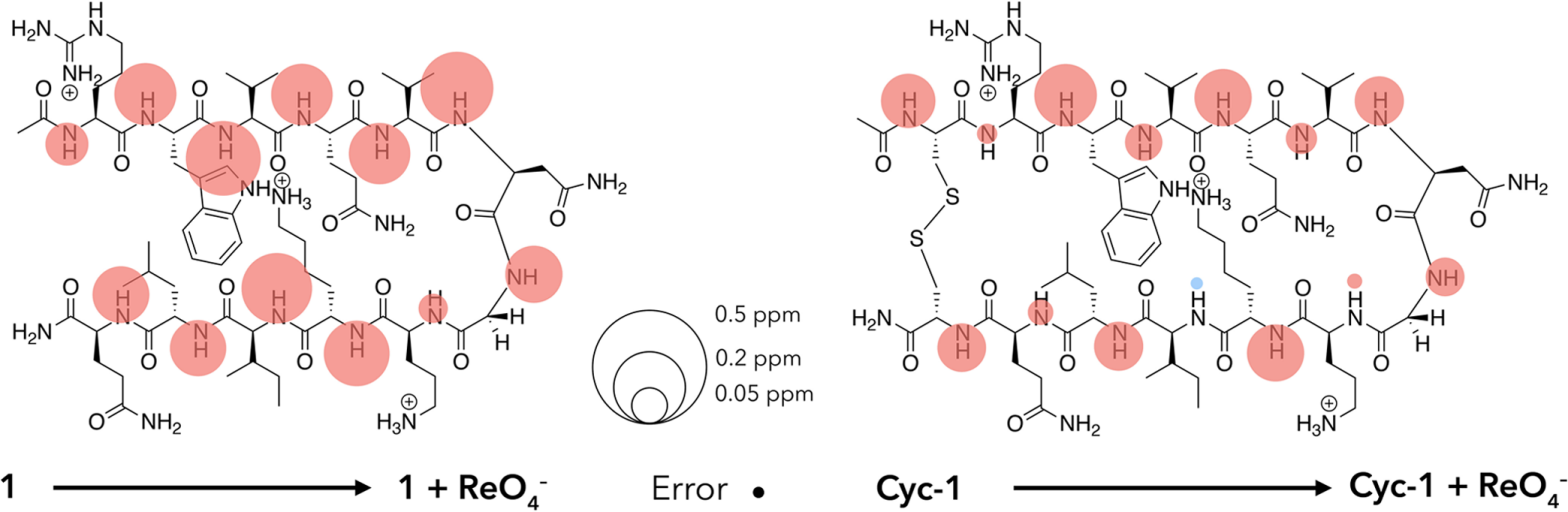
Bubble maps showing the referenced signal shifts of the mainchain amide groups of recipient **1***(left*) and **Cyc-1** (*right*) for the addition of 230 mM ReO_4_^–^. In each map, the effect of adding ReO_4_^–^ to the recipient to form a complex is defined by Δδ = (δ_N–H_ values for **1** (or **Cyc-1**) + salt) – (δ_N–H_ values for **1** (or **Cyc-1**)). Upfield signals are shown in red, and downfield shifts in blue. Where shifts are small, the circle is shown above or below the amide H atom. A scale and error-bubble (±0.005 ppm) are shown in the *lower center*.

Figure 5 shows the (referenced) responses of **1** and **Cyc-1** to the addition of ReO_4_^–^ (final concentration = 230 mM, equivalent bar graphs are given in Section 4 of the SI). The general upfield signal shifts are commensurate with anion binding.^63^ Consider first the outward-pointing N–H groups. A comparison of the differences between similar residues in **1** and **Cyc-1** reveals that the responses of the W2, Q4, and L11 residues are similar. In contrast, N6, G7, and K9 undergo significantly larger shifts in the case of recipient **1**. We attribute the relatively large shifts in N6 and G7 to the sensitivity of these turn residues to (un)folding. Relatedly, we attribute the relatively large upfield shift of K9 and L11 in **1** to an alteration in the cation-π interaction in the core. This dove-tails with the AF-HDX data described below.

Regarding the inward-pointing amide groups, in general, the signal shifts of **1** are much larger than the comparable shifts in **Cyc-1**. We attribute this difference to the inability of **Cyc-1** to unfold; it is difficult for the anion to intercalate into the structure of this recipient. Examining the responses of like-residues in both peptides, the largest differences between **1** and **Cyc-1** are between the pairs of residues R1, V3, V5, I10, and Q12.^64^ These represent the core of hairpin **1** (V3, V5, and I10) and its frayed terminus residues (R1 and Q12). We interpret these shifts to two potential binding sites: the cation-π-hydrophobic core (W2, K9, and L11) in which the anion can intercalate into the pincer-like terminal region (R1 and Q12) of **1** that can chelate an anion. We initially suspected that one potential site for anion interactions in these peptides was at the turn,^65^ where there are the free amide N–H groups of N6 and G7, as well as the exposed C_α_-H atoms of G7. However, the data in Figure 5—particularly the small downfield shifts in the N6 and G7 amide signals of **Cyc-1**—does not support this idea. Rather, the amide shift data suggest ReO_4_^–^ targets the cation-π-hydrophobic core region of **1**.

With Cl^–^ (Section 4, SI), much smaller upfield shifts indicative of limited hairpin structure breaking and/or weak anion association^63^ were observed. However, the differences in the maps of Cl^–^ and ReO_4_^–^ complex formation with **1** were not very informative (Section 4 of the SI), and similar conclusions were obtained with combinations of **2** and the three anions. Thus, although all bubble maps revealed that anion binding is focused on the terminal region and the cation-π-hydrophobic core rather than the turn “half” of the peptide (∼_4_TVNGOK_9_,), the data did not shed light on the mechanism of selectivity (Table 1).

We anticipated that the affinity of anions to the recipients would be weak, and to confirm this, we carried out titration studies involving **1** and the anions Cl^–^, ClO_4_^–^, and ReO_4_^–^. In each case, we monitored all amide N–H shifts as a function of salt to produce—using the standard 1:1 model^66^—a matrix of affinity constants reported by each residue for each guest (Section 4.3, SI). The obtained binding constants ranged from 0.37 to 3.36 M^–1^, with the average values of all residues for each anionic guest  ranging between 1.31 and 1.82 M^–1^. Considering the errors associated with each fitting (±15%), we do not read too much into these data other than to confirm that binding is weak.

We also examined anion binding by using molecular dynamics (MD) simulations. Because of the difficulties with accounting for dispersion forces, charge transfer, and induced fit of these relatively large systems, MD simulations can provide only a crude picture of anion binding. Nevertheless, we examined recipient **1** in the presence of excess NaCl, NaI, or NaClO_4_. These (200 ns) simulations were performed in bulk water at 25 °C and 1 bar, with the peptide modeled using the Amber-ff03 all-atom force field,^67,68^ the ions modeled using the generalized Amber force field (GAFF)^69^ and their partial charges obtained from AM1-BCC calculations,^70^ and the water modeled using TIP4P-Ew.^71^ To identify binding areas, we analyzed the anion trajectories (TRAVIS^72,73^) during the MD simulations, extracting and rendering as volumes the spatial distribution functions (ChimeraX^74^ for visualization). The net charge on the peptide was set to +3 to represent its protonation state at pH 2.3. Each simulation included one peptide and thirty-three anions in a bath of three-thousand waters. All simulations were run in the isothermal–isobaric ensemble, with the temperature and pressure maintained using the Nosé–Hoover thermostat^75,76^ and the Parrinello–Rahman barostat.^77^ These simulations confirmed the higher affinity of charge-diffuse ions, and the propensity of anions to bind to nonpolar pockets and crevices on the folded structure. However, similar to NMR chemical shifts, they could not provide precise information about anion binding. Full details of the simulation results are given in Section 7 of the SI.

### Mapping Anion Binding: Anion-Facilitated HDX (AF-HDX)

With only limited success in using chemical shift data to map anion binding, we sought an alternative approach. Rationalizing that anion complexation would accelerate hydronium-catalyzed amide N–H exchange by facilitating hydronium ion intercalation into the peptide structure, we turned to hydrogen–deuterium exchange (HDX) experiments. Ideally, baseline conditions in such experiments would be salt-free. However, with the necessity of a low pH, truly salt-free conditions were not possible. Nevertheless, we anticipated that 50 mM sodium phosphate buffer (pH = 2.3) as a reference state would still serve our purposes and that the addition of 230 mM salt would lead to measurable rate changes.

**Figure 6:**
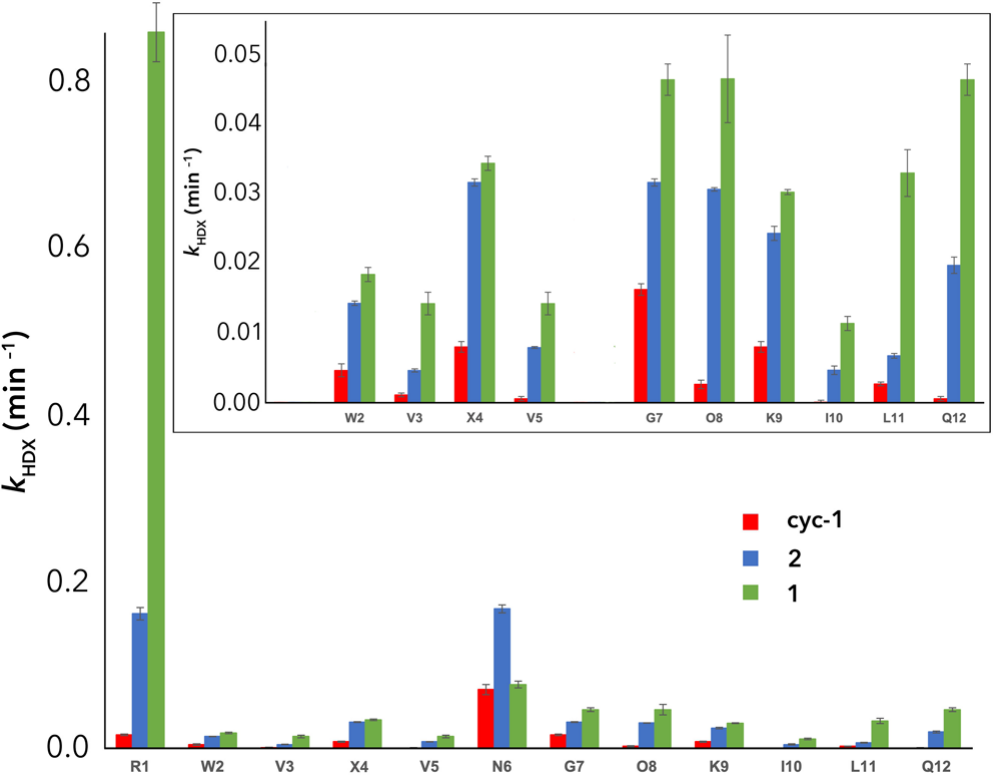
Hydrogen–deuterium exchange rates (*k*_HDX_, min^–1^) for peptides **1**, **2**, and **Cyc-1**. In the *inset*, the data for the fast-exchanging R1 and N6 have been removed to highlight differences in the rate constants for the slower-exchanging amides.

Our studies first examined peptides **1**, **Cyc-1**, and **2** in the absence of added salt (Figure 6 and Section 5, SI). Commensurate with the estimation of their degree of folding using the G7 global reporter methylene (52, 100, and 79% respectively), we found that with only one exception (N6 in **2**), all residues exchanged more slowly in **Cyc-1** than in **2**, than in **1**. Two mainchain amides were found to exchange particularly fast: R1 and N6. These fast rates were attributed to the exposed nature of the residues. This noted, in small peptide models, positively charged residues typically slow the rate of hydronium-catalyzed HDX rates via Coulombic interactions,^78,79^ and inductive and steric effects.^80^ We assume that the effects of the limited extent of folding of **1** and the N-terminal position of R1 is much larger than any effect from the guanidinium side chain, but it should be acknowledged that another possibility is the known affinity of phosphate (buffer) for guanidiniums^14^ and buffer anion binding facilitating exchange.

In contrast, the slowest exchanging amide protons in all peptides were for the inward-pointing residues V3, V5, and I10 in the cation-π-hydrophobic core (Figure 6, *inset*). These three protons exchanged at approximately the same rate, slightly slower than the exchange rate of the amide of W2. This last exchange rate is itself relatively slow because the amide is screened on one face by the bulky side chains of W2 and L11.^80^ In contrast, the amides of G7, O8, and Q12 exchange relatively quickly. We attribute these faster exchanges to the absence of a side chain in G7, the poor hydrogen bonding of the O8 amide because of its tight proximity to the turn, and the frayed C-terminal nature of Q12. In this light, the exchange rates of X4 and L11 can be interpreted as typical, fully exposed amide groups in these hairpin structures.

**Figure 7:**
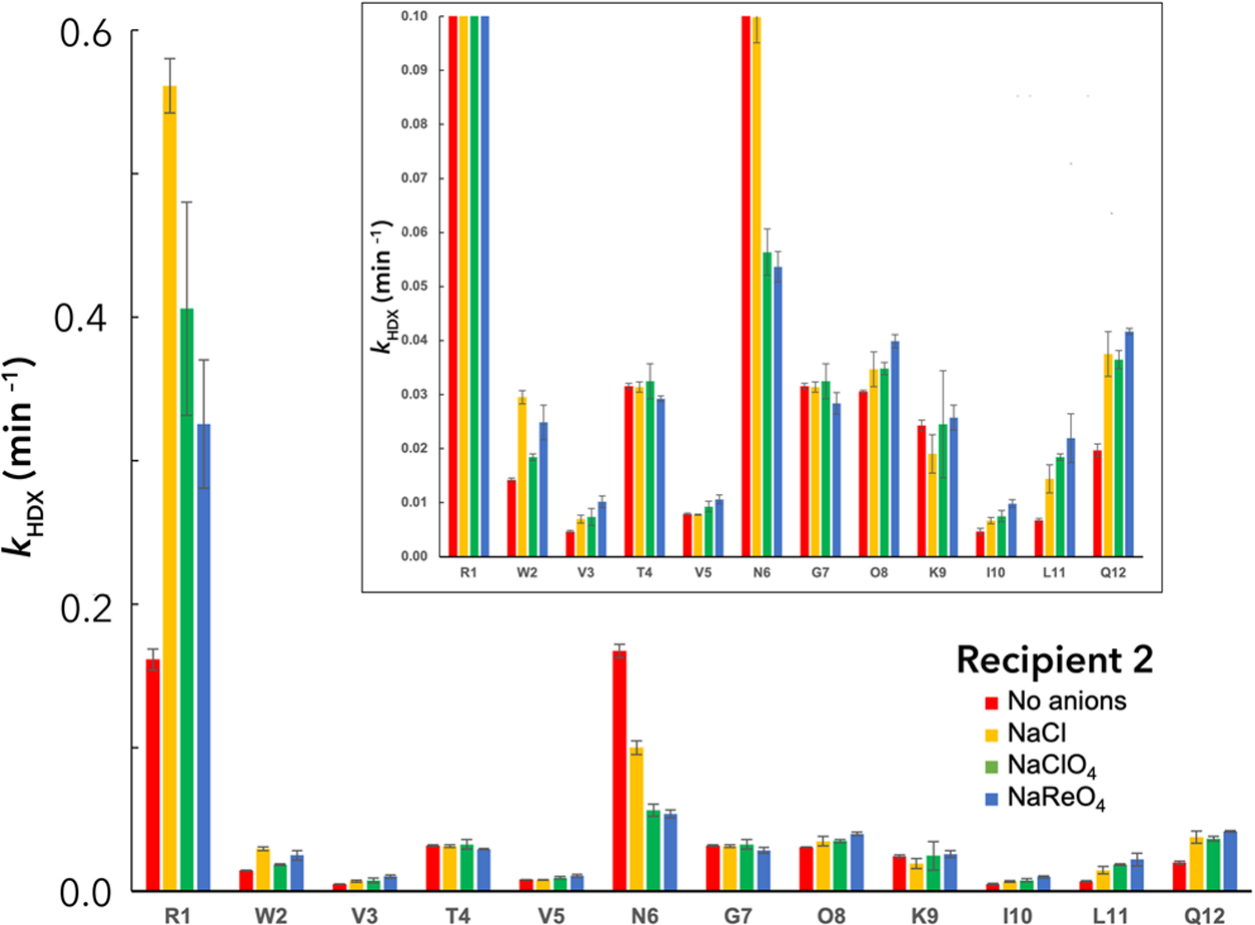
Hydrogen–deuterium exchange rates (*k*_HDX_, min^–1^) for peptide **2** as a function of added Cl^–^, ClO_4_^–^, and ReO_4_^–^. In the *inset*, the data have been rescaled to emphasize the exchange rates of the slower-exchanging amide protons.

We selected recipient **2** to probe how the HDX rates were affected by the presence of Cl^–^, ClO_4_^–^, and ReO_4_^–^ (Figure 7). Commensurate with the idea of anion binding to the side chain of R1 and/or the chelating amide groups of the termini, HDX rates were increased substantially with the addition of all anions. Moreover, the observed anion dependency meshes with the known higher affinity of Cl^–^ anion for guanidiniums^13^ relative to charge-diffuse anions.

For the slowest exchanging V3, V5, and I10, the addition of anions increased *k*_HDX_. Moreover, the trend in the data for each residue is that charge-diffuse anions promote exchange more than charge dense Cl^–^. This is in accord with both the trends in the ability of these anions to unfold the peptide, and the known affinity of charge-diffuse anions for nonpolar surfaces.^21,26−34^ Indeed, this trend in *k*_HDX_ as a function of the nature of the anion is also observed with an inward-pointing O8. Together, this data suggests an opening up of the peptide core and unfolding. The remaining two inward-pointing N–H groups (R1 and Q12) do not follow this trend, but their exchange data are complicated by their terminal positions.

In contrast, for the outward-pointing amide groups, trends in the data are more complicated. L11 follows the aforementioned trend of increasing anion charge-diffusivity leading to increased *k*_HDX_, which may reflect its proximity to the core of the peptide. Additionally, *k*_HDX_ at N6 is seen to decrease with increasing anion charge-diffusivity, indicative of a loss of exposure because of breakdown of the hairpin structure and hence loss of its unique turn conformation. However, T4 and G7 proved to be essentially insensitive to the nature of the anion, whereas the W2 and K9 amides show no distinct trend.

**Figure 8:**
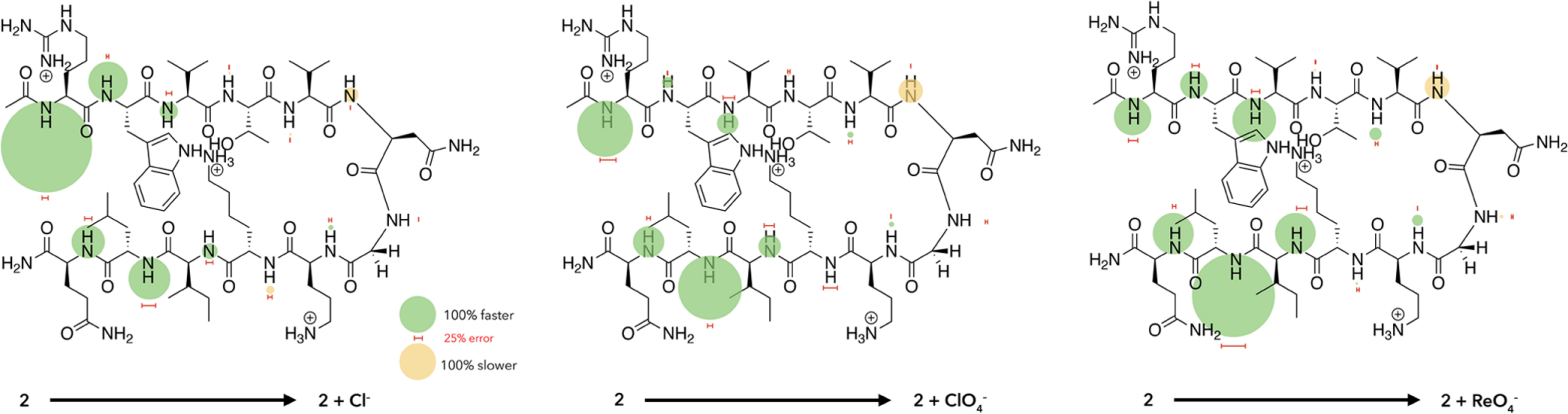
Percentage HDX rate differences for recipient **2** in the absence (*k*_HDX-0_) and presence (*k*_HDX-salt_) of 230 mM Cl^–^ (*left*), ClO_4_^–^ (*center*), and ReO_4_^–^ (*right*). In the figure, the areas of the bubble are proportional to the percentage change in exchange rate (= [*k*_HDX-salt_ – *k*_HDX-0_]/*k*_HDX-0_ × 100%), with increases shown in green and attenuations shown in yellow. All values are shown to the same scale for illustrative purposes, including the small percentage changes at the turn half of the hairpin (see the main text). The associated errors are shown with red horizontal bars. A scale for both the values and the determined errors is shown in the *lower center*. For reference, specific values and errors are given in Figure 7.

An alternative way to look at these data is the HDX difference bubble maps shown in Figure 8, which show how the addition of Cl^–^, ClO_4_^–^, or ReO_4_^–^ differentially affect the rate of exchange of each mainchain amide N–H. These patterns of HDX changes reveal a bifurcation of the peptide. For the terminal “half” (_1_RWV_3_, and _10_ILQ_12_), there are large increases in exchange rates, whereas in the turn “half” _4_TVNGOK_9_, the responses from each residue are much weaker and mixed. In this turn half of the peptide, the changes in *k*_HDX_ induced by Cl^–^, ClO_4_^–^, and ReO_4_^–^ were not significant for one-third of the data: T4 (Cl^–^ and ClO_4_^–^), V5 (Cl^–^ only), G7 (Cl^–^ and ClO_4_^–^), and K9 (ClO_4_^–^ and ReO_4_^–^). However, the remaining 18 differences were significant. The largest change observed was a slowing of the exchange of N6, indicating again a relative loss of access from the fully folded shape to a more random peptide structure. Overall, however, the rate changes at the turn were small compared to the rate changes determined in the terminal “half” of the peptide, suggesting hydronium access to the turn region was largely independent of the presence and nature of anions. Thus, if anion binding occurs at the turn, binding does not significantly affect the local structure.

In the terminal “half”, all residues reported a substantial increase in *k*_HDX_ rates. These general increases are interpreted as resulting from binding of the anion to the region and the facilitation of the hydronium-catalyzed exchange process. Strikingly, the patterns of changes in response to Cl^–^, ClO_4_^–^, and ReO_4_^–^ are distinctive. For Cl^–^, the focus of change is at R1, whereas this focus shifts toward L11 in the case of ClO_4_^–^ and quite definitively moves to L11 upon the addition of ReO_4_^–^. Our interpretation of this data is that small, charge-dense Cl^–^ can both bind to the guanidinium side chain of R1^13,14^ and be chelated by the R1 and Q12 mainchain N–Hs. In contrast, charge-diffuse ClO_4_^–^ and ReO_4_^–^ are weak hydrogen bond acceptors and preferentially bind to nonpolar regions of solutes,^21,26−34^ and as a result bind into the cation-π-hydrophobic core. In the simplest of terms, binding to the core could involve intercalation between W2 and K9, or between W2 and L11, and the bubble maps suggest that ClO_4_^–^ binding involves intercalation between W2 and L11, whereas ReO_4_^–^ binding is focused more between W2 and L11.^81^ In either case, intercalation leads to a relocation of both the indole ring of W2 and the isobutyl side chain of L11, resulting in greater exposure of the mainchain amide groups and facilitation of hydronium-catalyzed exchange. Referring back to the change in the percentage fold of the hairpin upon addition of 230 mM salt (−3.4 and −11.3% for Cl^–^ and ReO_4_^–^ respectively), we conclude that Cl^–^ is—as measured by (distal) global reporter G7—a weaker denaturant because it is chelated by the terminal amide groups R1 and Q12 and most directly affects the part of the peptide with least secondary structure, whereas at the other extreme, ReO_4_^–^ intercalates deeply into the cation-π-hydrophobic core and so affects a greater degree of denaturation.

Folded proteins and peptides are most easily understood using the two-state model, whereby the molecule is either folded or unfolded. However, the HDX data strongly points to intermediate states of unfolding, where Cl^–^ binding disrupts approximately one-third of the peptide, whereas ReO_4_^–^ binds into the cation-π-hydrophobic core disrupting approximately one-half of the fold. How the mapping data emerging from AF-HDX experiments can be reconciled with the two-state model is, as of yet, uncertain.

## Conclusions

The studies outlined here demonstrate that all of the salts investigated cause specific denaturation of the β-hairpins investigated. Using G7 as a global reporter of the extent of folding, we find that the more charge diffuse an anion is, the greater its ability to unfold the hairpin. ^1^H NMR chemical shift data and MD simulations point to the key role of anion binding in these denaturation processes. However, these binding events are too weak to quantify accurately. As an alternative, we have sought to qualify anion binding by mapping; both by using the chemical shift and the exchange rate of each mainchain amide. Regarding the former, ^1^H NMR chemical shifts are in accord with the weakening of the secondary structure as a result of anion binding. However, this approach only maps anion binding to the half of the hairpin nearer the termini and does not differentiate between different anions. In contrast, examining AF-HDX rate changes demonstrates two different mechanisms of anion-induced denaturation. Thus, assisted by the N-terminal R1, charge-dense Cl^–^ is chelated by the terminal N–H groups and induces a small degree of unfolding. In contrast, charge-diffuse anions intercalate deeply into the cation-π-hydrophobic core and, thus, induce a greater extent of denaturation. AF-HDX therefore offers an unprecedented view of anion binding, bringing specificity to what are normally described as nonspecific NCIs. Our understanding is that the processes identified here represent just two examples of how anions can induce the salting-in Hofmeister effect. Consequently, we are investigating other hairpin sequences to learn more about how anions directly interact with folded peptides as well as the thermodynamic consequences of such events. These findings will be reported in due course.

## Data Availability

The data that support the findings of this study are available in the SI of this article.

## Supporting Information Available

Materials, instrumentation, and sample preparation; peptide characterization; NMR titration with anions; binding analysis and fitting; hydrogen−deuterium exchange (HDX) experiments; anion binding to Cyc−2: Hα and side chain analysis; and molecular dynamics simulations and spatial distribution functions (PDF)
