# Supplementary material for: Anion-Facilitated Hydrogen–Deuterium Exchange as a Tool to Probe Weak Anion–Protein Interactions Responsible for Hofmeister Effects
Source: J Phys Chem B. 2025 Feb 13;129(8):2235–45. doi: 10.1021/acs.jpcb.4c08619 (PMC11873968; doi:10.1021/acs.jpcb.4c08619)
Supplement: Supplementary file 1 — jp4c08619_si_001.pdf [file jp4c08619_si_001.pdf]

Supporting Information

Anion facilitated hydrogen-deuterium exchange as a tool to probe weak anion-protein interactions responsible for Hofmeister Effects

Thien H. Tran,[1] Meghan Ricciardi,[2] Lilly I. Grunski,[1]
William C. Wimley,[3] Marcey L. Waters,[2] Bruce C. Gibb[1]

[1] Department of Chemistry
Tulane University School of Science and Engineering
New Orleans, LA 70118, USA

[2] Department of Chemistry
University of North Carolina at Chapel Hill
Chapel Hill, NC 27599, USA

[3] Department of Biochemistry and Molecular Biology
Tulane University School of Medicine
New Orleans, LA 70112, USA

Thien H. Tran orcid.org/0009-0001-9359-5829
Meghan Ricciardi orcid.org/0000-0003-3249-2241
Lilly I. Grunski orcid.org/0009-0002-4112-9727
William C. Wimley orcid.org/0000-0003-2967-5186
Marcey L. Waters orcid.org/0000-0002-4917-5755
Bruce C. Gibb orcid.org/0000-0002-4478-4084

1. Materials, instrumentation, and sample preparation S4
1.1. Peptide synthesis and purification S4
1.1.1. Peptide sequences S4
1.1.2. General peptide synthesis protocol S4
1.1.3. Peptide workup and cleavage protocol S4
1.1.4. Cyclization of fully folded β-hairpin controls S5
1.1.5. LCMS Data of β-Hairpin peptides S5
1.2. Other materials and instrumentation S5
1.3. NMR sample preparation S5
1.4. Circular dichroism (CD) sample preparation S5
2. Peptide characterization S7
2.1. NMR assignments S7
2.2. Quantification of folding S19
2.2.1. Fraction folded S19
2.2.2. Backbone proton shifts analysis S19
2.2.3. CD characterization S21
2.3. Structure prediction by AlphaFold and energy minimization S22
2.4. Orientation of the 4th residue side chain S32
3. NMR titration with anions S34
3.1. Peptide 1 ..... S34
3.2. Peptide 2 ..... S36
3.3. Peptide cyc-1 ..... S38
3.4. Peptide cyc-2 ..... S39
4. Binding analysis and fitting S41
4.1. Anion affinity S41
4.2. Free energy of unfolding S44
4.3. Anion affinity determination S45
5. Hydrogen-deuterium exchange (HDX) experiments S46
5.1. Without salts S46
5.2. With salts S48
6. Anion binding to Cyc-2: H_α and side chain analysis S50
7. Molecular dynamics simulations and spatial distribution functions (SDFs) S54
References S56

Acronyms and abbreviations

Ac	Acetyl
ACN	Acetonitrile
DCM	Dichloromethane
DIC	*N,N'*-diisopropycarbodiimide
DMF	*N,N*-dimethylformamide
Eq	Equivalent
Fmoc	Fluorenylmethoxycarbonyl
HPLC	High performance liquid chromatography
LCMS	Liquid chromatography – mass spectroscopy
NMR	Nuclear magnetic resonance
O (in a peptide sequence)	Orn – ornithine
Oxyma	Ethyl cyanohydroxyiminoacetate
TFA	Trifluoroacetic acid

1. Materials, instrumentation, and sample preparation

1.1. Peptide synthesis and purification

1.1.1. Peptide sequences

Four β-hairpin peptides were chosen for this study based on previous report from the Waters group (Figure S1).[1] To assess their percentage fold, the corresponding cyclic peptides of **1** and **2** (Cyc-**1** and Cyc-**2**), as well as 6-mer half peptides (**3-5**) were also synthesized and used as references.

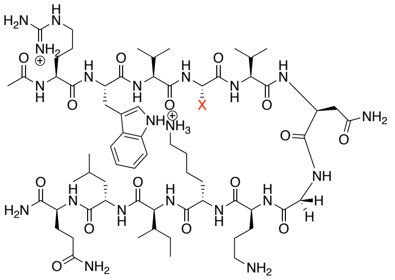

1: X = Gln
2: X = Thr

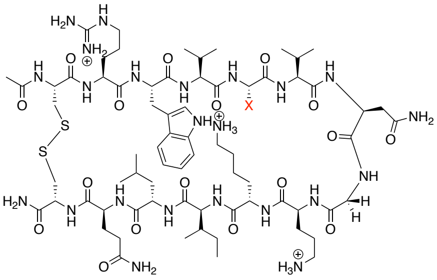

Cyc-1: X = Gln
Cyc-2: X = Thr

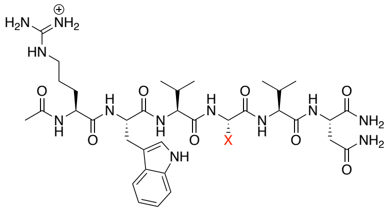

3: X = Gln
4: X = Thr

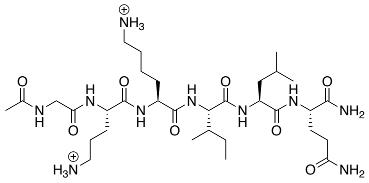

5

Figure S1. *Peptides' primary structures in the study*

Table S1. *One-letter code sequence of each peptide in the study*

Peptide	Sequence
**1**	Ac-RWVQVNGOKILQ-NH_2
**2**	Ac-RWVTVNGOKILQ-NH_2
**Cyc-1**	Ac-CRWVQVNGOKILQC-NH_2
**Cyc-2**	Ac-CRWVTVNGOKILQC-NH_2
**3**	Ac-RWVQVN-NH_2
**4**	Ac-RWVTVN-NH_2
**5**	Ac-GOKILQ-NH_2

1.1.2. General peptide synthesis protocol

All peptides (except **2** and **4**, which were purchased from GenScript and confirmed with mass spectroscopy analysis) were synthesized utilizing a CEM Liberty Blue peptide synthesizer. Standard Oxyma-DIC couplings were conducted with Fmoc protected amino acids with Rink Amide AM resin. Five eq of each canonical amino acid (4 eq of non-canonical amino acids) were coupled using 5 eq of Oxyma and 10 eq of DIC during two coupling cycles at 90 °C for 4 minutes (10 minutes for Arg) in DMF. Deprotection utilized a solution of 20% piperidine in DMF bubbled through the resin for two cycles (1 minute long each), with washing the resin with DMF between each cycle.

1.1.3. Peptide workup and cleavage protocol

All peptides had the final Fmoc deprotection occur in a peptide flask with subsequent *N*-terminal acetylation. Each β-Hairpin was transferred to a peptide flask and bubbled with a 20%

piperidine in DMF solution for 15 minutes each, twice, washing with DMF between cycles. After the deprotection, 6% 2,5-lutidine and 5% acetic anhydride in DMF was added and bubbled through the resin for 30 minutes. The resin was then washed 3 times with DMF, followed by DCM and MeOH alternating 3 times ending on DCM. The peptide was then cleaved using 95%: 2.5%: 2.5% TFA:triisopropylsilane:water bubbling for 3 hours. Peptides are then purified via reverse phase column chromatography using a gradient of 0-1 % B with solvent A comprising of 95:5 water:ACN with 0.1% TFA and solvent B is 95:5 ACN:water with 0.1% TFA. Additional purification was conducted using reverse phase HPLC with a gradient of 0-70% B in 60 minutes with solvent A containing 95:5 water: ACN with 0.1% TFA and solvent B having 95:5 ACN: water with 0.1% TFA. Pure peptides were concentrated and lyophilized to dryness, with purity being confirmed via LCMS.

1.1.4. Cyclization of fully folded β-hairpin controls

Cyclic peptides **cyc-1** and **cyc-2** were formed via disulfide bond formation of cystine residues at *C* and *N* terminus of the peptides following previous protocols.[2] After cleavage of the cyclic peptides from the resin and reverse phase column purification, lyophilized peptides are then dissolved in 10mM phosphate buffer pH 7.4 with 1% DMSO and left overnight at room temperature. Upon completion confirmed via LCMS, peptides are then purified via reverse phase HPLC as described above.

1.1.5. LCMS Data of β-Hairpin peptides

The main LCMS peaks and their expected mass are summarized in the table below.

Table S2. *Expected and observed mass of the peptides from LCMS*

Peptide	Expected Mass (amu)	Observed Mass (amu)
**1**	1495.80	1494.76
**2**	1468.75	1468.40
**Cyc-1**	1700.06	1698.89
**Cyc-2**	1673.03	1671.56
**3**	841.97	841.41
**4**	814.94	814.90
**5**	712.89	712.52

1.2. Other materials and instrumentation

All sodium salts were purchased from Aldrich (purity > 99%) and were used without further purification. All solutions were prepared in ultra-pure water (resistivity of 18.2 MΩ·cm at 25 °C). All peptide structures were characterized by collecting NMR spectra on a Bruker 700 MHz spectrometer (Louisiana State University). Titration experiments were performed on a Bruker 400 MHz. Hydrogen-deuterium exchange (HDX) rate assays were conducted on the Bruker 700 MHz. Peptide secondary β-hairpin structure was predicted using the deep learning-based model AlphaFold. The obtained structures were then minimized using the Gaussian 16 package; and the resulting structure confirmed by NMR and circular dichroism (CD). All data were analyzed by either Matlab 2022a or Microsoft Excel.

1.3. NMR sample preparation

All salts were prepared as a stock solution of 4 M concentration. The (initial) concentration of peptide solutions were 5 mM, and unless otherwise noted, were formed in 90% H_2O:10% D_2O buffered to pH 2.3 with 50 mM H_3PO_4.

1.4. Circular dichroism (CD) sample preparation

CD spectra were obtained using a Jasco J-810 spectropolarimeter. The hairpin peptides were dissolved in a solution containing 10 mM phosphate buffer and the pH of the solution adjusted to 2.30. The sample volume was 200 µL containing 100 µM of each peptide. Wavelength scans ranging from 260 to 190 nm were conducted using a quartz cuvette with a path length of 0.1 cm and a slit width of 1 nm. Baseline was also collected, and all spectra were all baseline-subtracted.

1.5. Error analysis

Error propagation analysis[3] was conducted on all dataset. Generally, in ^{1}H NMR the error in chemical shift is ± 0.005 ppm, whilst the error in temperature for variable temperature studies is ± 0.1 K. Using these values translates to fraction folded uncertainties of 2-3%. The error in amide chemical shift change ($\Delta\delta$) was obtained from data triplication, with a maximum error (standard deviation) of ± 0.015 ppm.

2. Peptide characterization

2.1. NMR assignments

1D NMR spectra were collected using 32 K data points, 16 scans, and 1–3 s presaturation. TOCSY, ROESY, and COSY experiments were performed with the pulse sequences DIPSI2ESGPPH, ROESYESGPPH, and COSYDFESGPPHPP respectively. 2D NMR experiments involved 8–16 scans in the first dimension, and 16–64 scans in the second dimension. Mixing times of 0.2 s were used in the ROESY experiments. Assignments were made by using standard methods as described by Wüthrich.[4] Chemical shift values, and TOCSY, spectra are given below for peptides **1**, cyc-**1**, **2**, cyc-**2** (Tables S3-S6, Figures S2-S11). For ROESY, we show here only for **1** and cyc-**1**, since NOEs in the rest are similar (raw data for all peptides are available). Obtained chemical shift values for half peptides **3-5** are given in Tables S7-S9).

Table S3. *Chemical shift values (δ, ppm) for peptide **1***

Residue/ Group	Amide N–H	H_α	H_β	H_γ	H_δ	H_ε	Side chain N–H
N-Acetyl		1.82					
R1	8.00	4.19	1.56	1.47, 1.39	3.01		7.13, 7.00
W2	8.18	4.86	3.02	7.13, 7.31	7.39	7.31	10.06
V3	8.51	4.20	1.92	0.77			
Q4	8.36	4.62	1.93, 1.83	2.12			7.34, 6.69
V5	8.55	4.07	1.89	0.81			
N6	9.02	4.46	2.89, 2.67				7.50, 6.83
G7	8.47	3.96, 3.69					
O8	7.87	4.43	1.80	1.71, 1.62	2.93		7.52
K9	8.35	4.52	1.60	1.19	1.33	2.60	7.24
I10	8.72	4.32	1.75	1.34, 1.08	0.79		
L11	8.25	4.06	1.32, 0.92	1.13	0.50, 0.37		
Q12	8.45	4.20	1.96, 1.80	2.19			7.30, 6.76
C-Amide							7.55, 7.01

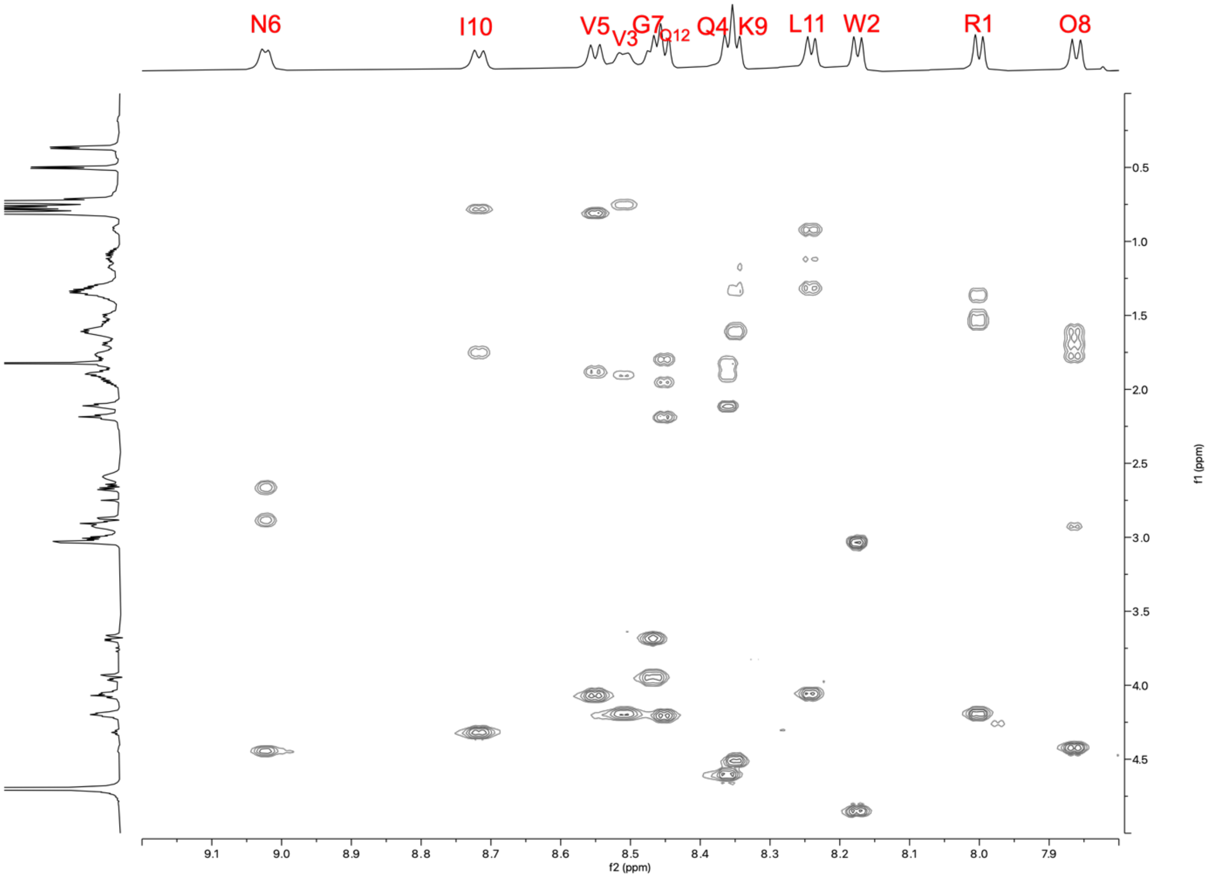

Figure S2. *TOCSY spectrum of **1** showing expected amino acid patterns.*

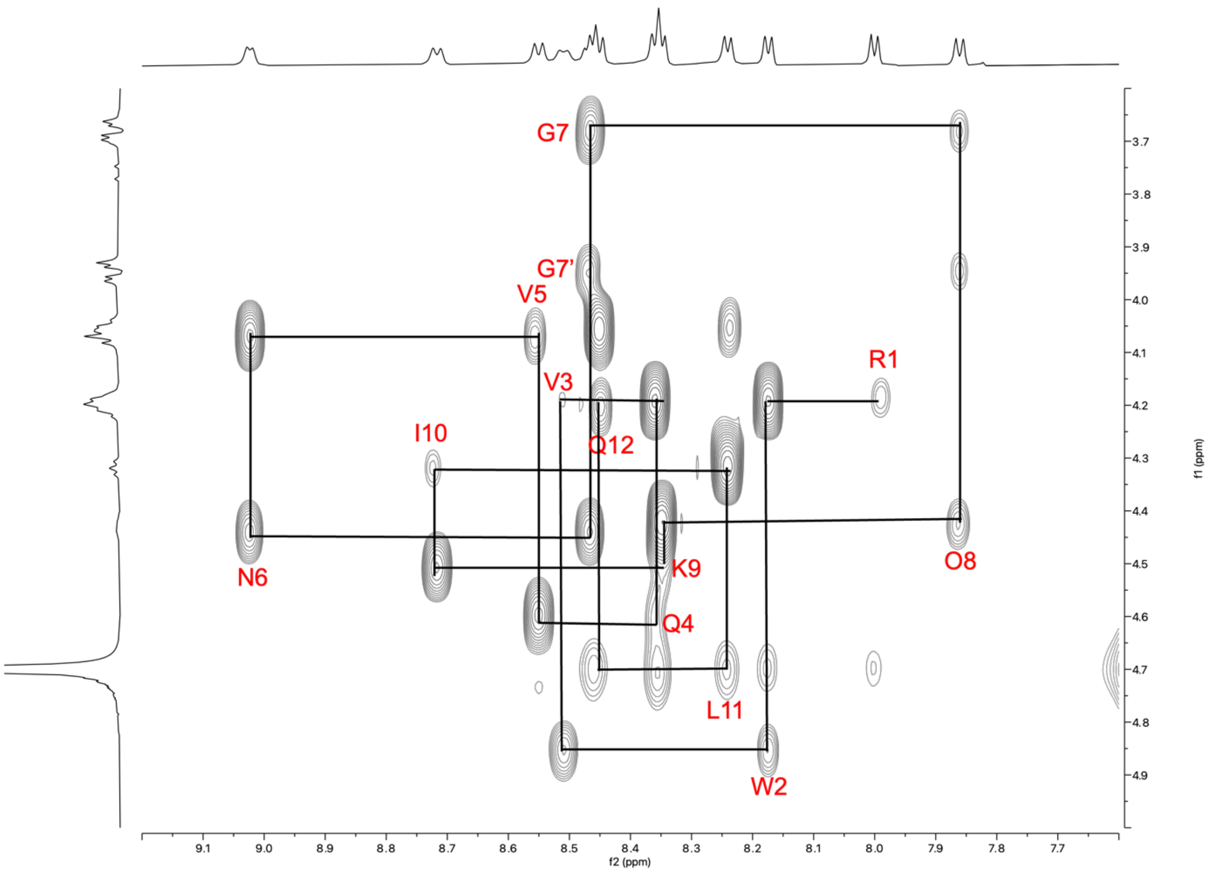

***Figure S3**. Inter-residue amide-C$_\alpha$H ROESY cross peaks of **1**. Intra-residue amide-C$_\alpha$H cross-peaks are labelled in red.*

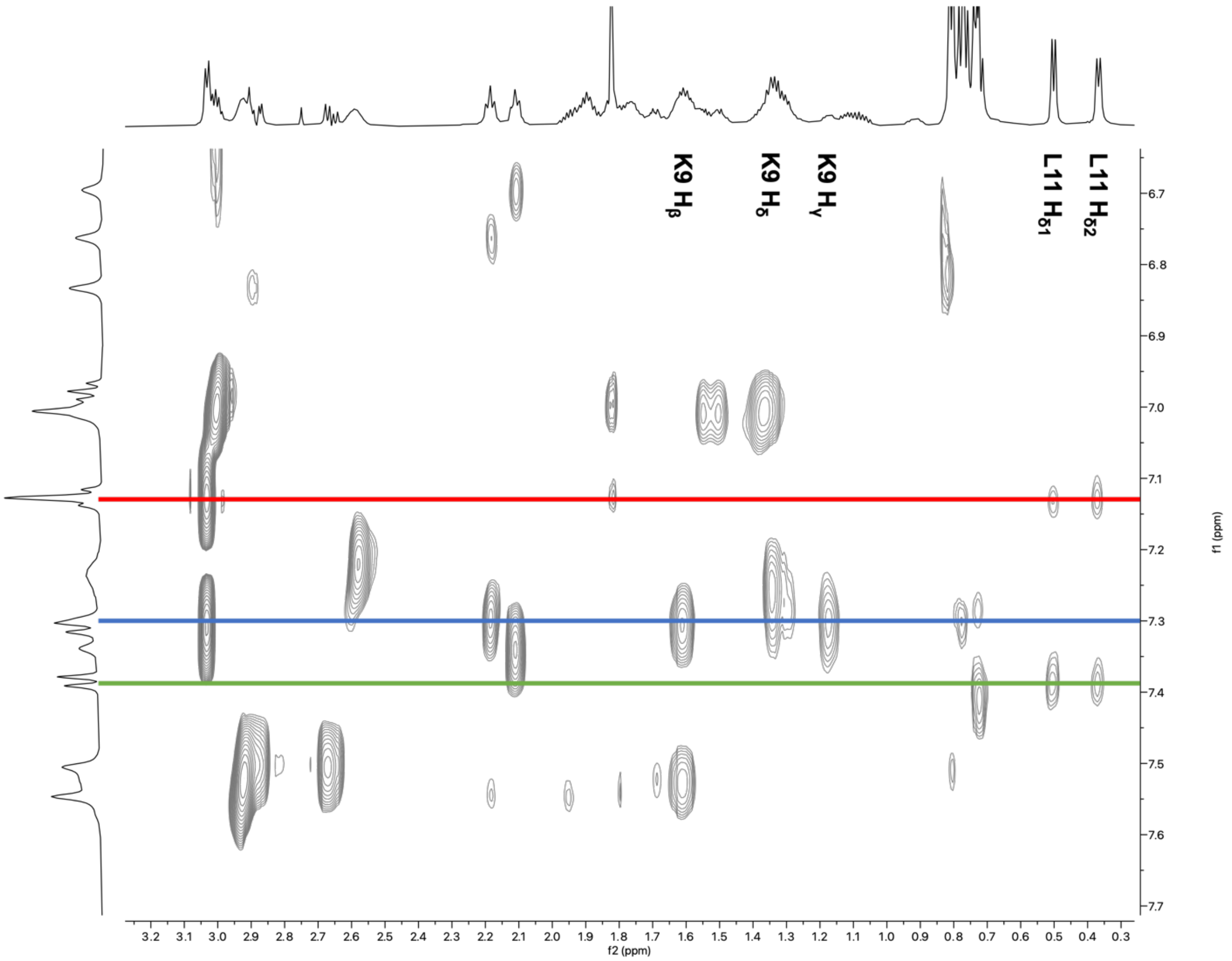

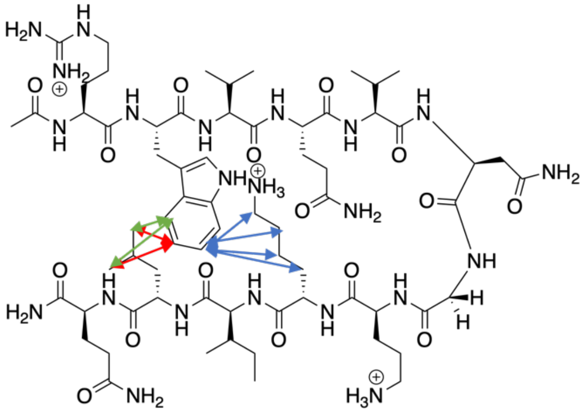

Strong NOEs

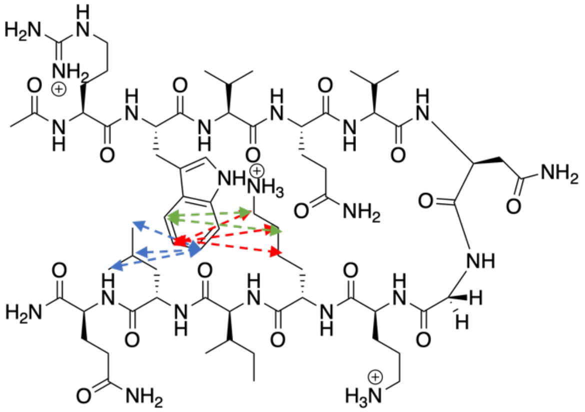

Weak NOEs

Figure S4. *ROESY cross-strand NOEs between W2 aromatic ring and other side chains of **1**. Lower structures: Strong NOEs are shown in bold arrows, whilst weak NOEs (dashed arrows) were observed with increased signal intensity. Upper spectrum: Horizontal lines (red, blue, green) represent the corresponding aromatic protons of W2 depicted in the structures.*

Table S4. *Chemical shift values (δ, ppm) for peptide* ***cyc-1***†

Residue/ Group	Amide N–H	H_{α}	H_{β}	H_{γ}	H_{δ}	H_{ε}	Side chain N–H
N-Acetyl		1.96					
C0	8.27	5.11	2.91, 2.31				
R1	8.62	4.51	1.72	1.59, 1.41	3.08		7.01
W2	8.57	5.04	2.99, 2.84	7.13, 6.99	7.68	7.38	10.05
V3	9.44	4.49	1.98	0.79			
Q4	8.47	4.98	1.87	2.07			7.29, 6.62
V5	8.92	4.16	1.83	0.81			
N6	9.49	4.32	3.02, 2.69				7.53, 6.85
G7	8.61	4.07, 3.57					
O8	7.69	4.60	1.80	1.78, 1.65	2.97		7.55
K9	8.45	4.89	1.65	1.21	1.34	2.44	7.09
I10	9.27	4.62	1.78	1.34, 1.10	0.79		
L11	8.25	3.81	1.21, -0.02	0.66	0.29, -0.44		
Q12	8.94	4.47	1.98, 1.74	2.16			7.23, 6.74
C13	8.83	4.95	2.95, 2.84				
C-Amide							7.43, 7.12

† Residues are numbered starting from 0 to avoid confusion.

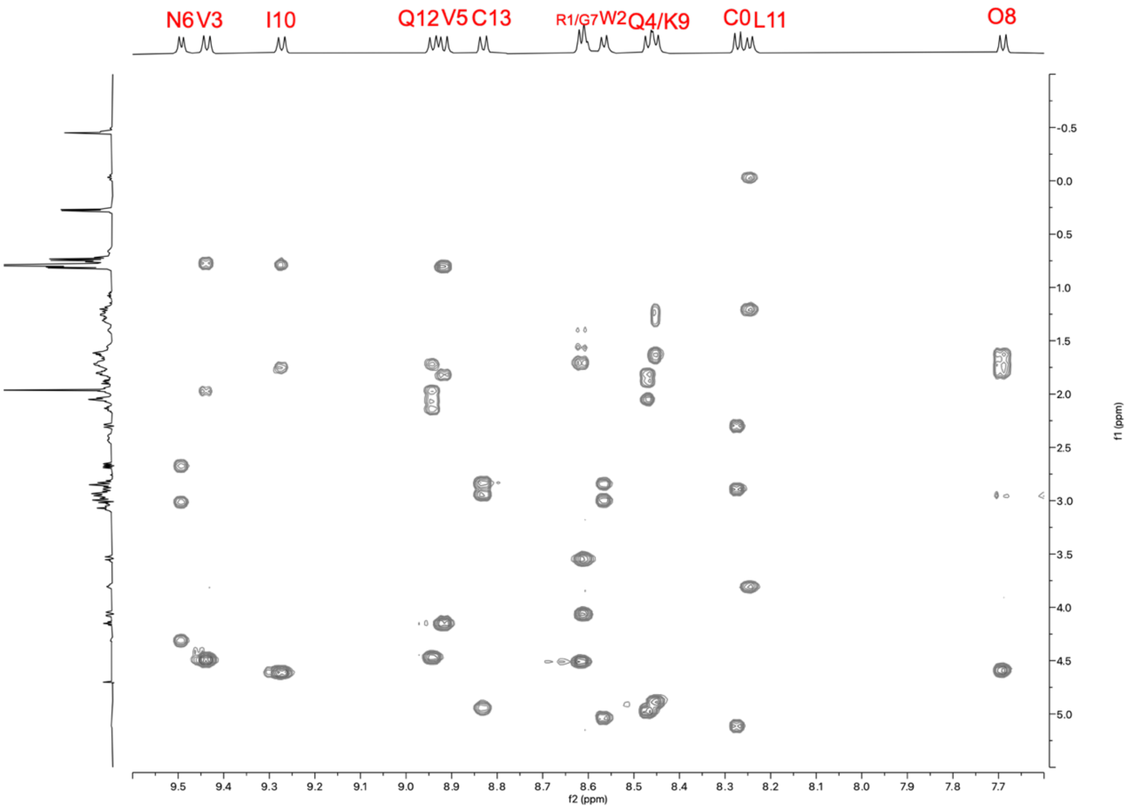

Figure S5. *TOCSY spectrum of* ***cyc-1*** *showing expected amino acid patterns*

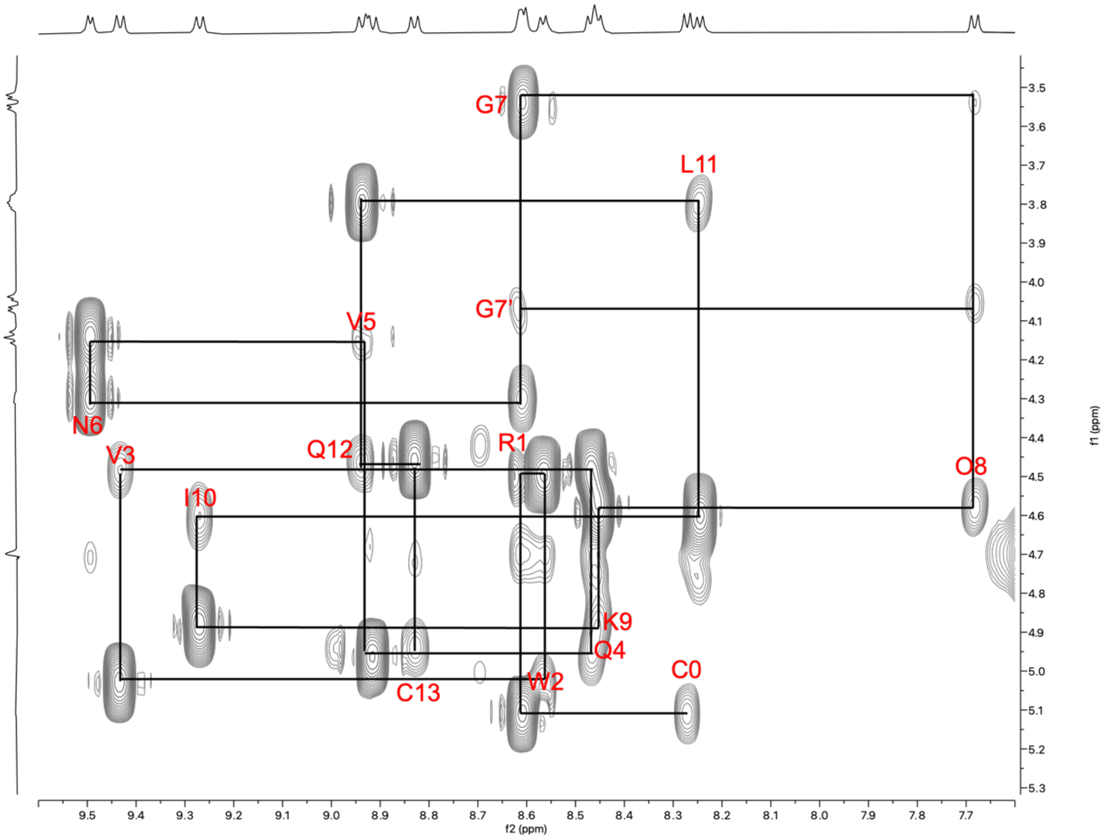

***Figure S6**. Inter-residue amide-C$_{\alpha}$H ROESY cross peaks of **cyc-1**. Intra-residue amide-C$_{\alpha}$H cross peaks are labelled in red*

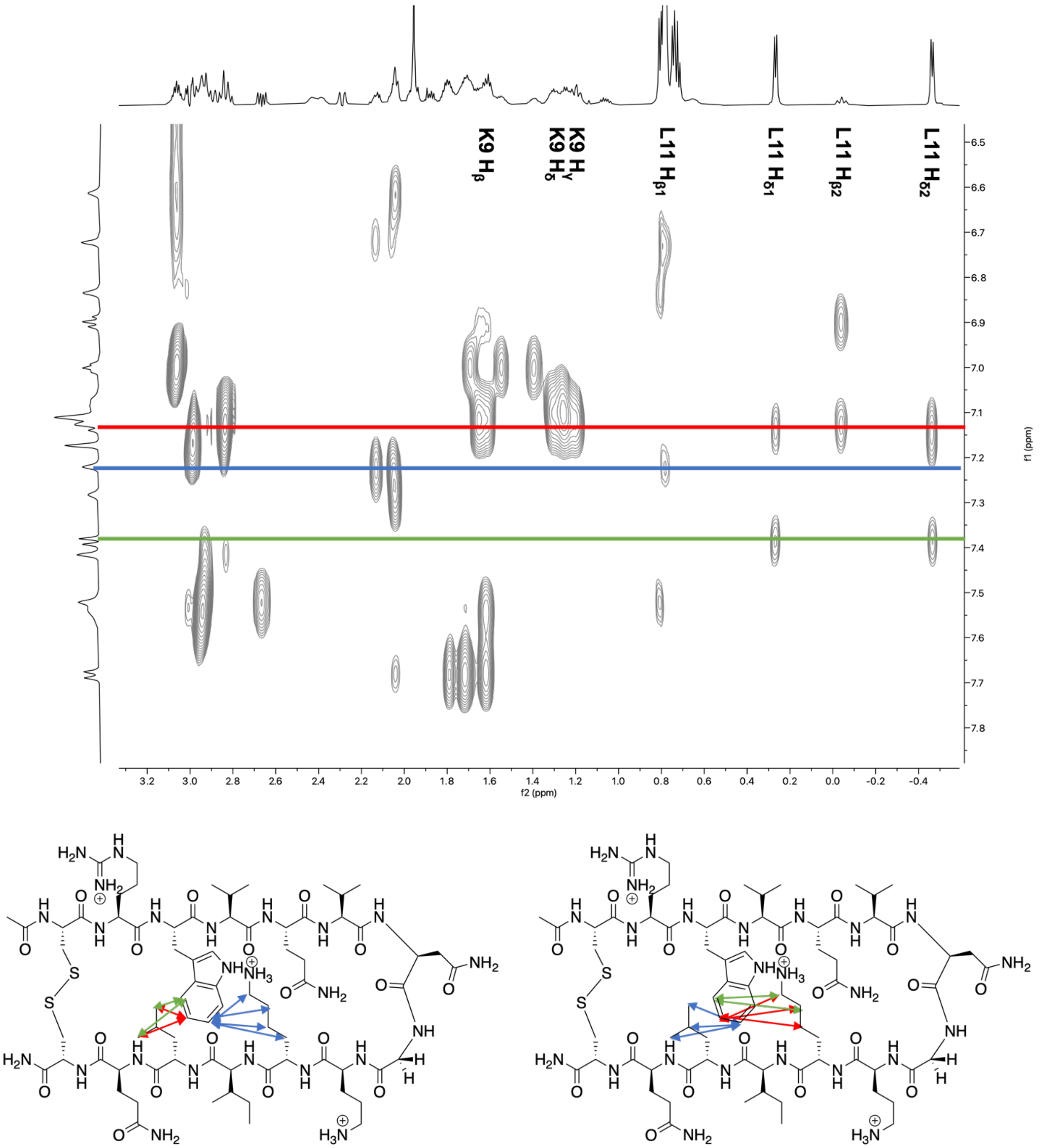

Figure 7. *ROESY cross-strand NOEs between W2 aromatic ring and other side chains of cyc-**1**. Lower structures: Strong NOEs are shown in bold arrows, whilst weak NOEs (dashed arrows) were observed with increased signal intensity. Upper spectrum: Horizontal lines (red, blue, green) represent the corresponding aromatic protons of W2 depicted in the structures.*

Table S5. *Chemical shift values (δ, ppm) for peptide* ***2***

Residue/ Group	Amide N–H	H_α	H_β	H_γ	H_δ	H_ε	Side chain N–H
N-Acetyl		1.82					
R1	7.96	4.30	1.61	1.45	3.07		7.01
W2	8.25	4.99	3.00				10.07
V3	8.92	4.49	2.00	0.78			
T4	8.39	4.84	3.92	0.94			
V5	8.78	4.11	1.85	0.84			
N6	9.33	4.38	2.94, 2.67				7.54, 6.84
G7	8.38	4.01, 3.54					
O8	7.78	4.46	1.76	1.62	2.93		7.55
K9	8.45	4.72	1.59	1.19	1.27	2.51	7.14
I10	9.09	4.52	1.78	1.34, 1.07	0.77		
L11	8.20	3.94	1.22, 0.61	0.91	0.38, 0.17		
Q12	8.56	4.22	1.96, 1.79	2.17			7.24, 6.76
C-Amide							7.58, 7.01

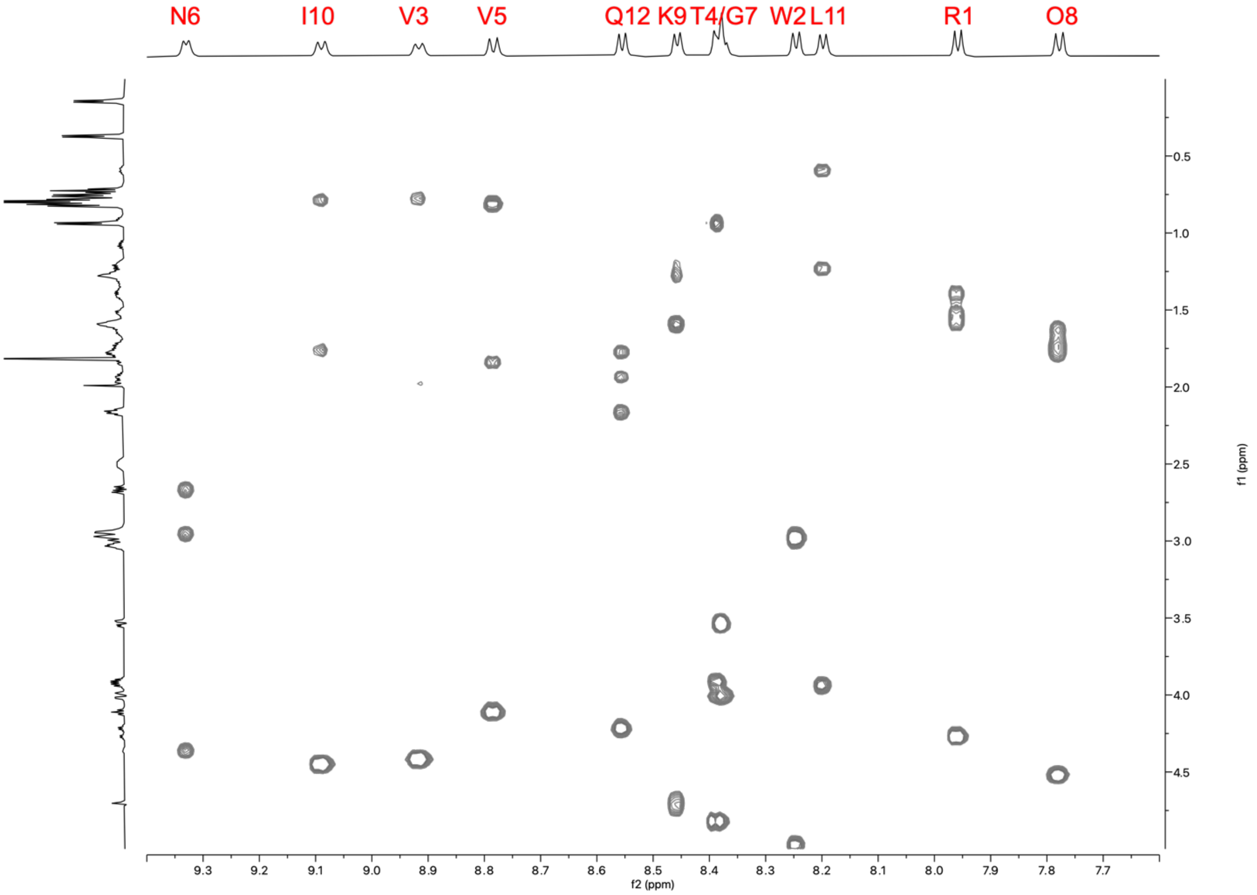

Figure S8*. TOCSY spectrum of* ***2*** *showing expected amino acid patterns.*

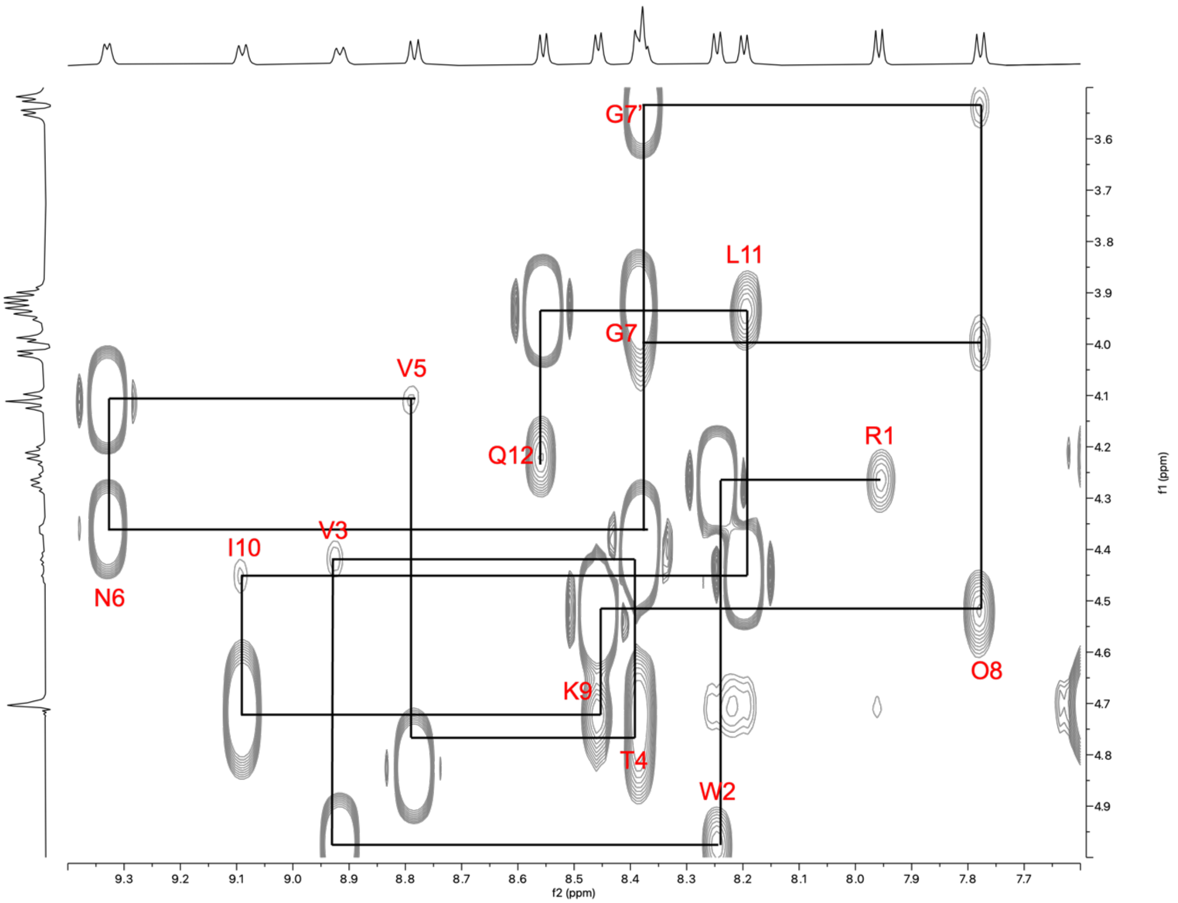

Figure S9. *Inter-residue amide-CαH ROESY cross peaks of* ***2***. *Intra-residue amide-CαH cross peaks are labelled in red.*

Table S6. Chemical shift values (δ, ppm) for peptide ***cyc-2***†

Residue/ Group	Amide N–H	Hα	Hβ	Hγ	Hδ	Hε	Side chain N–H
N-Acetyl		1.96					
C0	8.28	5.13	2.91, 2.31				
R1	8.62	4.52	1.73	1.58, 1.42	3.07		7.01
W2	8.58	5.04	2.99, 2.86	7.39, 7.12		7.18	10.05
V3	9.43	4.53	2.00	0.78			
T4	8.47	4.96	3.88	0.91			
V5	8.98	4.14	1.81	0.81			
N6	9.57	4.30	2.99, 2.68				7.53, 6.85
G7	8.40	4.06, 3.46					
O8	7.71	4.60	1.82	1.72, 1.63	2.97		7.56
K9	8.53	4.95	1.63	1.23	1.30	2.49	7.10
I10	9.41	4.60	1.76	1.34, 1.07	0.81		
L11	8.21	3.79	1.20, -0.07	0.66	0.27, -0.43		
Q12	8.93	4.47	2.02, 1.73	2.14			7.23, 6.72
C13	8.83	4.95	2.94, 2.83				
C-Amide							7.49, 7.11

† Residues are numbered starting from 0 to avoid confusion.

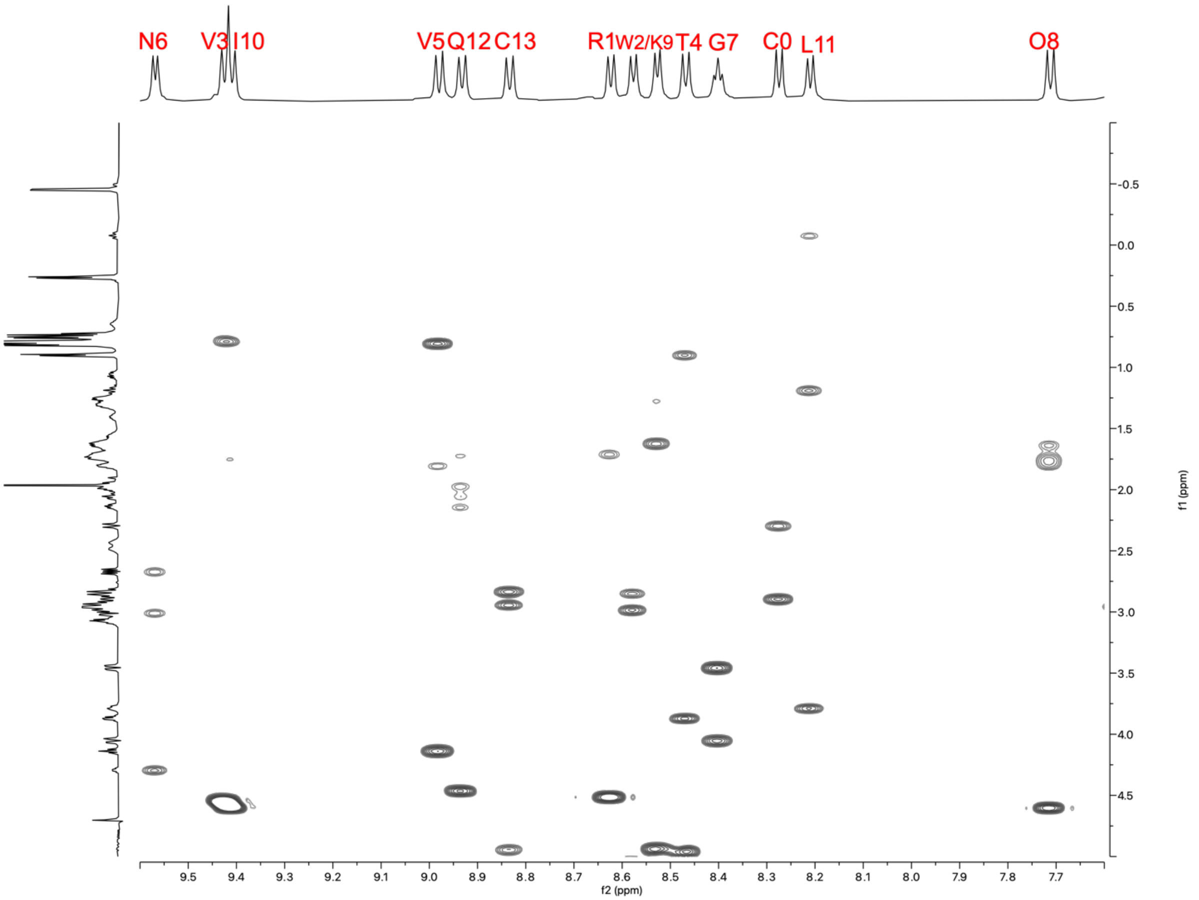

Figure S10*. TOCSY spectrum of* ***cyc-2*** *showing expected amino acid patterns.*

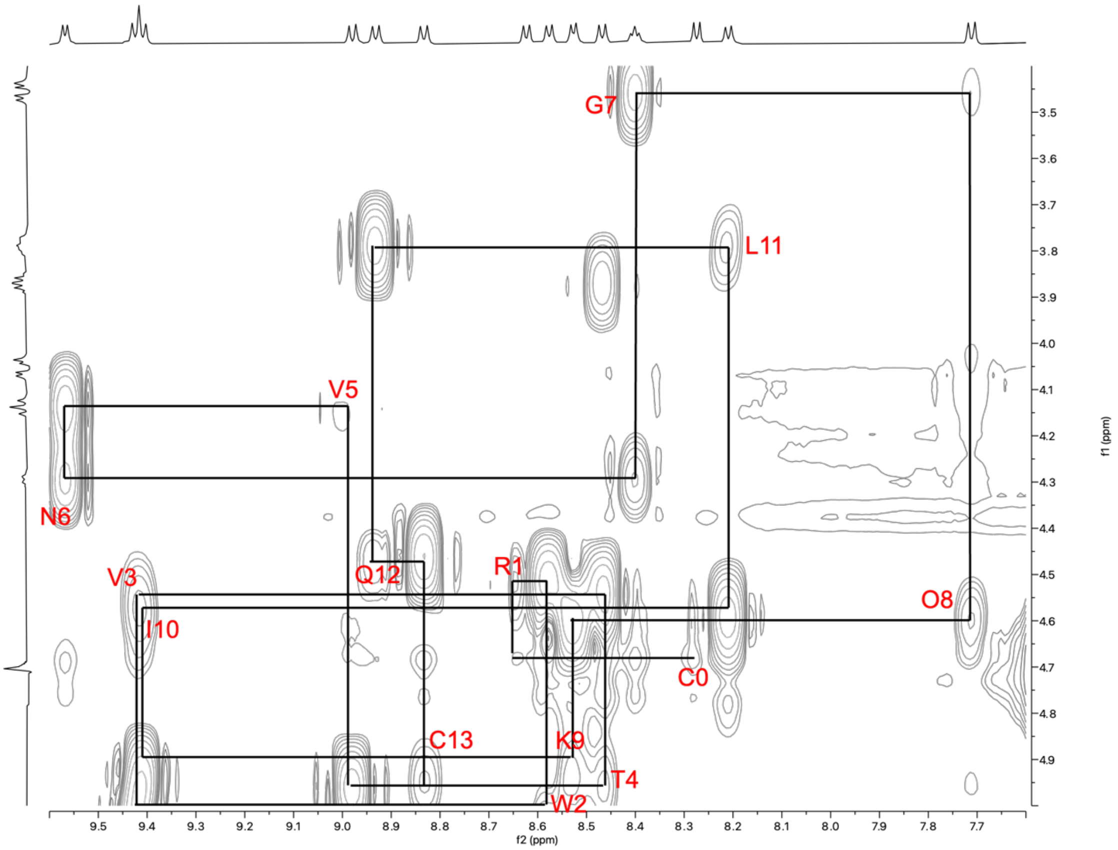

***Figure S11**. Inter-residue amide-H$_\alpha$ ROESY cross peaks of **cyc-2**. Intra-residue amide-H$_\alpha$ cross peaks are labelled in red.*

As references for peptides **1** and **2**, half-peptides **3**, **4**, and **5** were synthesized and characterized. Due to their poor solubilities, NMR spectra were collected at 1 mM for **3** and **4**.

Table S7. *Chemical shift values (δ, ppm) for peptide* ***3***

Residue/ Group	Amide N–H	H_α	H_β	H_γ	H_δ	H_ε	Side chain N–H
N-Acetyl		1.85					
R1	8.06	4.05	1.49	1.32	2.98		6.99
W2	8.06	4.62	3.22, 3.14	7.53, 7.40	7.13		10.04
V3	7.66	3.90	1.85	0.74			
Q4	8.19	4.15	2.20	1.89			
V5	8.15	3.99	1.98	0.83			
N6	8.42	4.60	2.74, 2.64				7.49, 6.81
C-Amide							7.14, 7.04

Table S8. *Chemical shift values (δ, ppm) for peptide* ***4***

Residue/ Group	Amide N–H	H_α	H_β	H_γ	H_δ	H_ε	Side chain N–H
N-Acetyl		1.83					
R1	8.04	4.02	1.47	1.37	2.96		6.96
W2	8.00	4.67	3.24, 3.13	7.55, 7.40	7.07		10.04
V3	7.76	4.03	1.91	0.77			
T4	8.10	4.25	4.05	1.10			
V5	8.12	4.02	1.99	0.83			
N6	8.39	4.58	2.72, 2.62				7.48, 6.81
C-Amide							7.12, 7.03

Table S9. *Chemical shift values (δ, ppm) for peptide* ***5***[†]

Residue/ Group	Amide N–H	H_α	H_β	H_γ	H_δ	H_ε	Side chain N–H
N-Acetyl		1.96					
G7	8.21	3.82					
O8	8.23	4.29	1.80	1.65	2.93		7.45
K9	8.30	4.21	1.66	1.31	1.30	2.92	7.51
I10	8.15	4.05	1.75	1.38, 1.11	0.79		
L11	8.28	4.29	1.61	1.48	0.82		
Q12	8.24	4.21	2.01, 1.91	2.28			7.44, 6.78
C-Amide							7.45, 7.03

[†] Residues are numbered starting from 7 to avoid confusion.

2.2. Quantification of folding

2.2.1. Fraction folded

To determine the chemical shifts of the fully folded state, 14-residue disulfide-linked analogs of peptides **1** and **2** were synthesized with a sequence of Ac-CRWVXVNGOKILQC-NH_2, where X represents either glutamine (Q) or threonine (T). A disulfide bond between C1 and C14 constrained the peptide into a β-hairpin. To determine the chemical shifts in the fully unfolded state, 6-mer peptides were synthesized with sequences Ac-RWVXVN-NH_2, Ac-GOKILQ-NH_2, where X represents either glutamine or threonine. The fraction folded was determined from global reporter G7 and equation (1).

$$\text{Fraction Folded (f)} = [\delta_{obs} - \delta_0]/[\delta_{100} - \delta_0] \qquad \textbf{(1)}$$

where δ_{obs} is the observed G7 signal split in the diastereotopic $C_\alpha H$ protons (in ppm) of the hairpin, δ_{100} is the G7 split (in ppm) of its cyclic form, and δ_0 is the G7 split of its corresponding 6-mer half peptide.

Table S10. *Fraction folded of **1** and **3**, determined from G7 splitting*

Peptide	δ_{obs} (ppm)	δ_0 (ppm)	δ_{100} (ppm)	Fraction folded
**1**	0.268	0	0.518	0.52
**3**	0.471	0	0.598	0.79

2.2.2. Backbone proton shifts analysis

a) Amide shifts

Backbone amide shifts difference between the hairpins and their 6-mer random coils were calculated as shown below. One should expect significant downfield shifts of hydrogen bonded cross-strand amide protons of three pairs: R1-Q12, V3-I10, and V5-O8. As Figure S18 shows, the R1 residue of hairpins **1** and **2** are evidently frayed. Residues N6 and O8 have relatively large downfield and upfield shifts respectively due to the nature of the type I’ turn. The amide N–H of L11 is evidently impacted by anisotropic (shielding) effects from the indole ring of W2 and undergoes a small upfield shift. To better illustrate the correlation between mainchain N-H shifts and the folded structure of the peptide, figure S19 displays the alteration in chemical shifts of backbone N-H atoms upon the connection of half peptides 6 and 7 to form hairpin **1** (Δδ = (δN-H values for **1**) - (δN-H values for **3** or **5**)), and the formation of Cyc-**1** by macrocyclization of **1** through the incorporation of a cystine bridge across the termini.

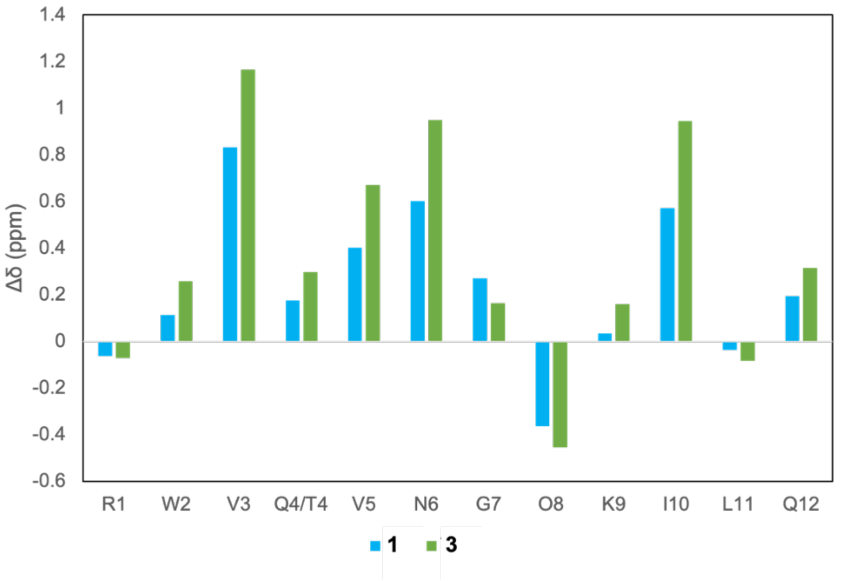

Figure S12. *Amide proton shift changes (*$\Delta\delta = \delta_{N\text{-}H\ of\ hairpins} - \delta_{N\text{-}H\ of\ random\ coils}$*) of* ***1*** *(Ac-RWVQVNGOKILQ-NH*$_2$*) and* ***2*** *(Ac-RWVTVNGOKILQ-NH*$_2$*) relatively to their 6-mer random coils by residue. The 4*th *residue is Gln for* ***1*** *and Thr for* ***2****. The more folded the peptide, the bigger change in amide proton chemical shifts.*

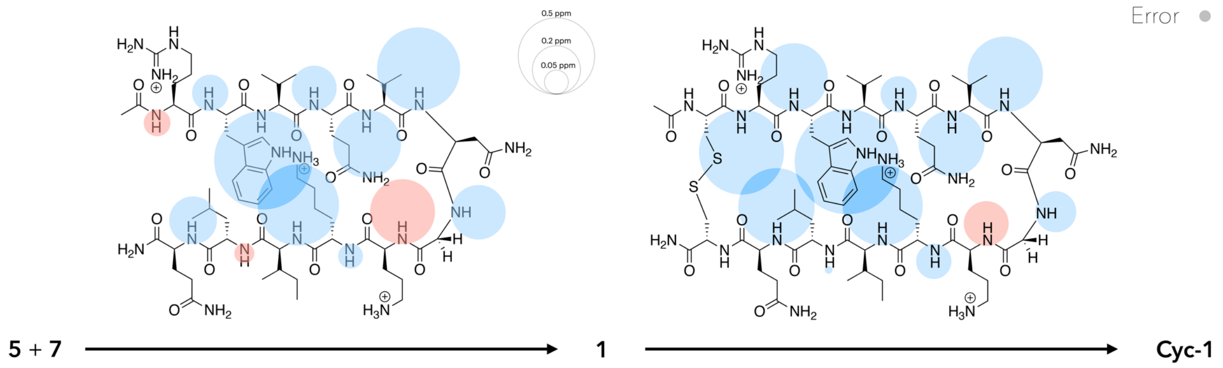

Figure S13. *Unreferenced response (Δδ) of amide N–H ^{1}H NMR signals upon the linking of half peptides* ***5*** *and* ***7*** *to generate hairpin* ***1*** *(Δδ = (*$\delta_{N\text{–}H}$ *values for* ***1****) – (*$\delta_{N\text{–}H}$ *values for* ***3*** *or* ***5****)), and the macrocyclization of* ***1*** *by the inclusion of a cystine bridge across the termini to generate* ***Cyc-1*** *(Δδ = (*$\delta_{N\text{–}H}$ *values for* ***Cyc-1****) – (*$\delta_{N\text{–}H}$ *values for* ***1****)). In both bubble maps, the areas of the circles associated with each N–H group are proportional to its ^{1}H NMR signal shift, with upfield signals shown in red and downfield shifts shown in blue (see scale, top center). Where shifts are small the circle is shown above or below the amide H atom. The error for ^{1}H NMR signal shifts (± 0.005 ppm) is shown top right.*

b) H_α shifts

Another common metric to quantify β-sheet structure are H_α shifts deviations from 6-mer coils.[5] Usually, there are upfield shifts in the backbone H_α if a peptide adopts α-helix conformation, and downfield shifts in backbone H_α signals if a β-sheet conformation is adopted.[6] As shown by the large H_α downfield shifts, significant β-sheet population was observed on both strands. The exception was for L11, which is affected by the anisotropic effects.

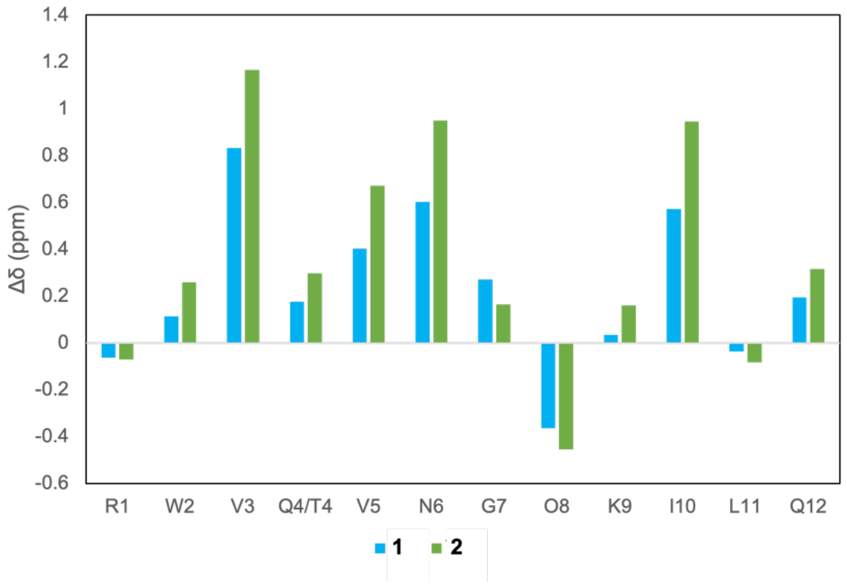

Figure S14. *Alpha proton shift changes (Δδ = $\delta_{H\alpha\ of\ hairpins} - \delta_{H\alpha\ of\ random\ coils}$) of of* ***1*** *(Ac-RWVQVNGOKILQ-NH_2) and* ***2*** *(Ac-RWVTVNGOKILQ-NH_2) relatively to their 6-mer random coils. The 4th residue is Gln for* ***1*** *and Thr for* ***2.*** *The more folded the peptide, the bigger change in alpha proton chemical shifts.*

To verify the random coil structures of **3-5**, their H_α shifts were compared with average random coil values, which have been widely used to determine secondary structures.[7] Figure S15 shows the chemical shift indexes (CSIs) of peptide **3-5**, suggesting their random coil structures in contrast to **1** and **2**. Note that since ornithine is a non-canonical amino acid, the value of lysine was used instead.

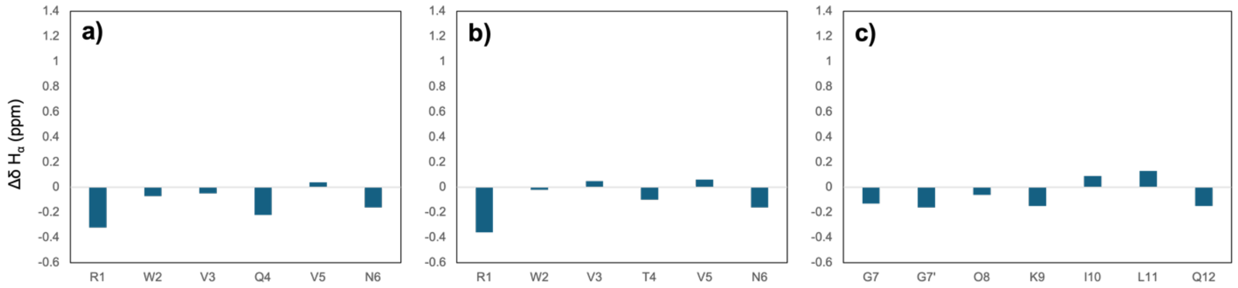

Figure S15. *Alpha proton shift changes of: a)* ***3*** *(Ac-RWVQVN-NH_2); b)* ***4*** *(Ac-RWVTVN-NH_2), and; c)* ***5*** *(Ac-GOKILQ-NH_2) relative to average random coil values (Δδ = $\delta_{H\alpha\ of\ 3\text{-}5} - \delta_{H\alpha\ of\ random\ coils}$). The y-axis is scaled as per Figure S14.*

2.2.3. CD characterization

CD spectra (with a buffer solution as baseline subtraction) were collected for five peptides **1-5** at a concentration of 100 μM at room temperature. The ellipticity at λ = 198 nm and λ = 216 nm is typically used to evaluate random coil and β-sheet structures respectively. The magnitude of the ellipticity at λ = 216 nm reflects the relative fraction folded of those peptides.

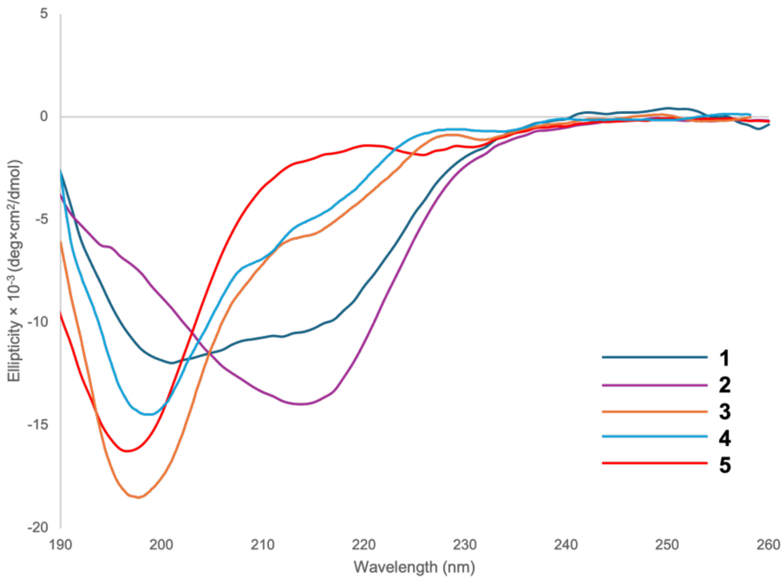

***Figure S16**. CD spectra at 298K of the peptides in this study. The negative ellipticity around λ = 198 nm and λ = 216 nm is indicative of random coil and β-sheet structures, respectively.*

2.3. Structure prediction by AlphaFold and energy minimization

The sequences of beta hairpin **1**, **cyc-1**, **and 2** were used to predict their 3-D structure using AlphaFold[8] with ColabFold[9] interface powered by Many-against-Many sequence searching (MMseqs2) cluster. The intrinsic cation-π interaction of the hairpin was characterized on the predicted structure using Gaussian 16 package with M06/6-31(d,p)[10] method. The optimized structure was then confirmed by ROESY-NMR[1]. More descriptions of the predicted structures are provided in Figure S18 and S19.

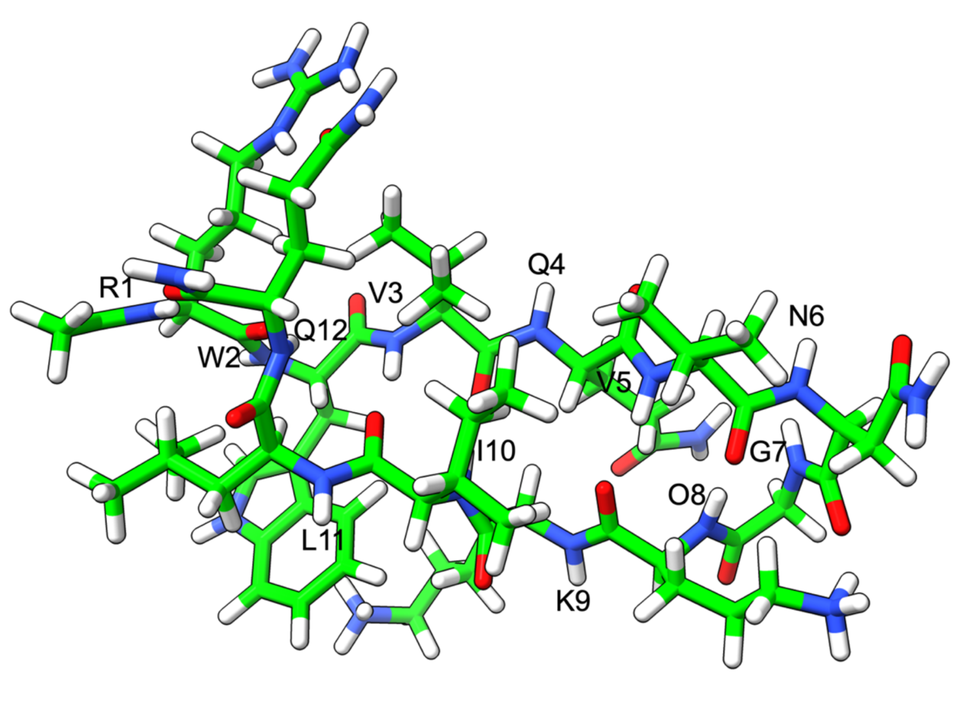

***Figure S17**. Energy minimized structure of **1**. All backbone amide protons are labelled.*

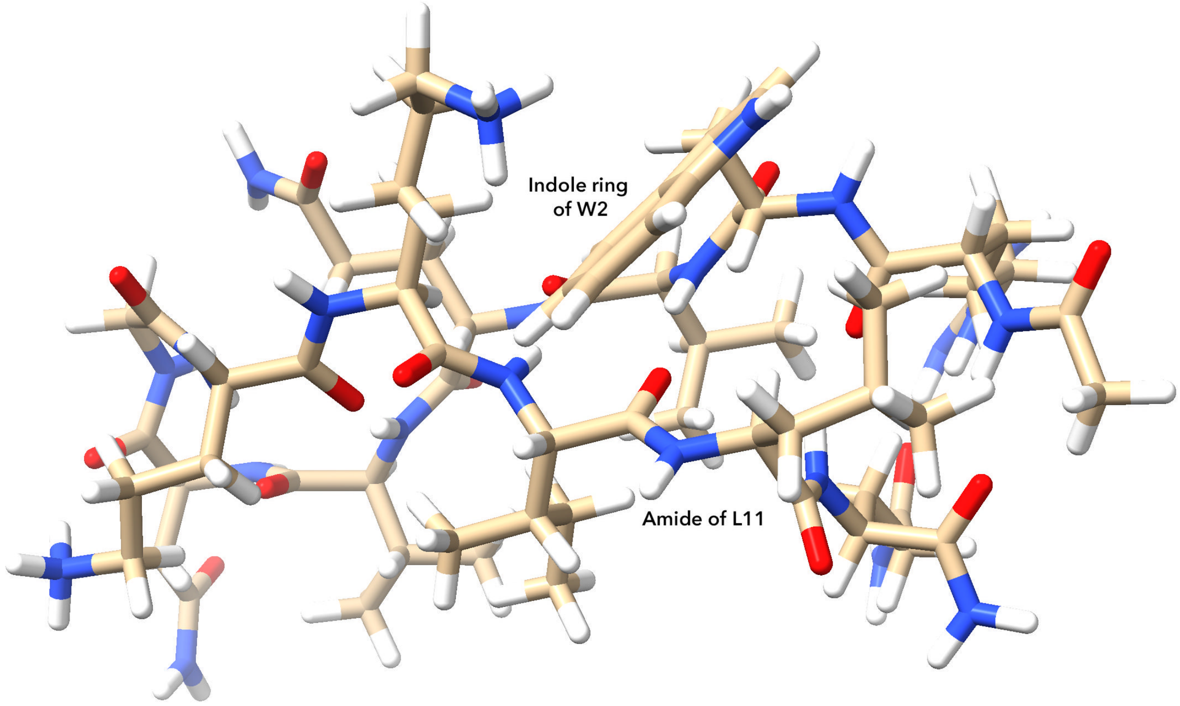

Figure S18. *Non-covalent interactions at the hydrophobic core K9 ammonium – W2 indole ring – L11 side chain of peptide* ***1****, which are also observed by ROESY NMR data.*

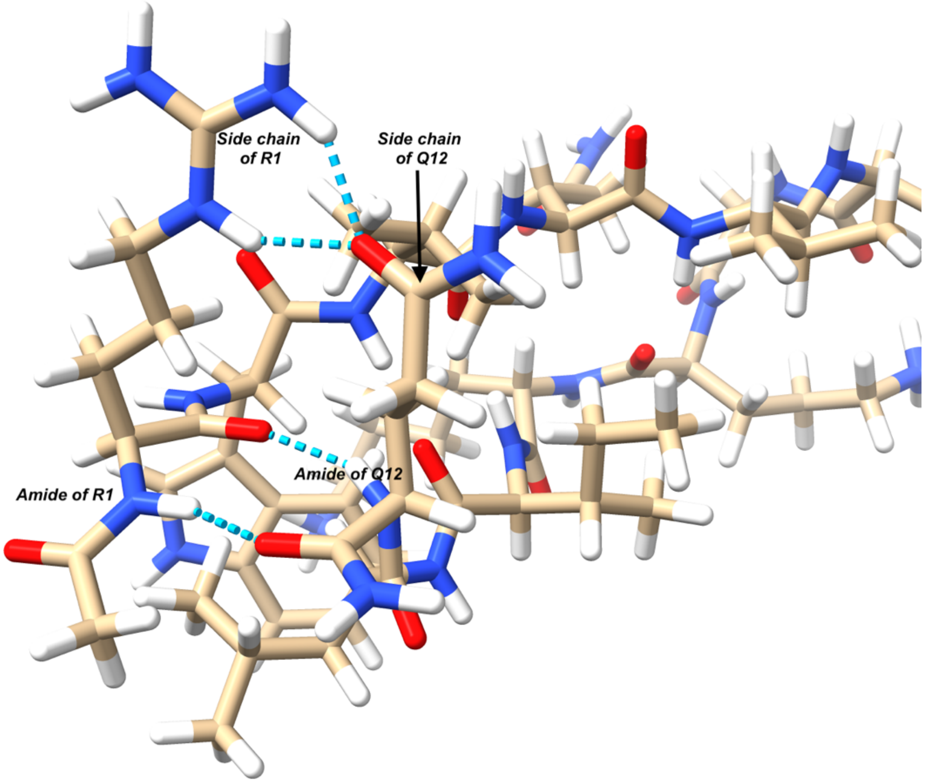

Figure S19. *Bifurcated side chain hydrogen bonding between R1 and Q12. Main chain and said chain hydrogen bonds are depicted by dashed cyan lines.*

Table S11. *Cartesian coordinates of the energy-minimized structure of* ***1.***

Atom	x	y	z
H	9.205	0.935	2.573
C	9.414	0.032	1.985
H	10.491	-0.147	2.033
H	8.865	-0.801	2.430
C	9.021	0.294	0.557
O	9.732	0.945	-0.207
N	7.813	-0.202	0.182
H	7.241	-0.732	0.843
C	7.289	0.076	-1.135
H	7.792	0.984	-1.494
C	7.555	-1.053	-2.145
H	7.321	-0.686	-3.155
H	8.633	-1.264	-2.122
C	6.750	-2.312	-1.866
H	6.916	-2.646	-0.828
H	5.676	-2.097	-1.961
C	7.083	-3.439	-2.822
H	6.921	-3.099	-3.855
H	8.139	-3.735	-2.728
N	6.204	-4.566	-2.546
H	5.742	-4.622	-1.634
C	5.944	-5.556	-3.394
N	6.564	-5.622	-4.576
H	7.366	-5.046	-4.779
H	6.362	-6.373	-5.218
N	5.017	-6.462	-3.073
H	4.613	-6.423	-2.138
H	4.933	-7.312	-3.609
C	5.797	0.303	-1.030
O	5.111	-0.336	-0.230
N	5.255	1.179	-1.894
H	5.841	1.681	-2.549
C	3.819	1.367	-1.917
H	3.479	1.417	-0.873
C	3.458	2.680	-2.615
H	2.367	2.687	-2.769
H	3.916	2.698	-3.614
C	3.873	3.853	-1.795
C	4.987	4.635	-1.939
H	5.746	4.616	-2.712
N	5.061	5.548	-0.911
H	5.783	6.244	-0.801
C	3.99 0	5.369	-0.072
C	3.629	6.044	1.096
H	4.238	6.852	1.491
C	2.458	5.646	1.728
H	2.144	6.153	2.637
C	1.664	4.601	1.212
H	0.742	4.331	1.727
C	2.037	3.921	0.058
H	1.429	3.096	-0.320
C	3.214	4.305	-0.602
C	3.135	0.186	-2.601
O	3.497	-0.230	-3.697
N	2.096	-0.286	-1.889
H	1.881	0.153	-0.996
C	1.142	-1.253	-2.371
H	1.250	-1.309	-3.465
C	1.394	-2.658	-1.784
H	0.717	-3.356	-2.302
C	2.827	-3.097	-2.058
H	3.090	-3.006	-3.119
H	2.985	-4.135	-1.741
H	3.525	-2.468	-1.484
C	1.101	-2.703	-0.290
H	0.049	-2.487	-0.064
H	1.328	-3.696	0.117
H	1.720	-1.965	0.243
C	-0.243	-0.690	-2.046
O	-0.366	0.450	-1.591
N	-1.311	-1.478	-2.258
H	-1.235	-2.407	-2.658
C	-2.644	-0.996	-1.945
H	-2.560	-0.301	-1.101
C	-3.243	-0.259	-3.146
H	-2.604	0.612	-3.346
H	-3.189	-0.919	-4.023
C	-4.674	0.212	-2.917
H	-5.388	-0.618	-3.007
H	-4.768	0.598	-1.894
C	-5.040	1.372	-3.816
O	-4.370	2.398	-3.846
N	-6.177	1.223	-4.542
H	-6.598	0.315	-4.674
H	-6.407	1.944	-5.211
C	-3.502	-2.180	-1.538
O	-3.441	-3.244	-2.146
N	-4.329	-1.933	-0.497
H	-4.310	-1.021	-0.035
C	-5.295	-2.902	-0.016
H	-5.377	-3.691	-0.776
C	-4.868	-3.525	1.324
H	-4.732	-2.687	2.027
C	-5.955	-4.448	1.861
H	-6.875	-3.909	2.121
H	-6.212	-5.223	1.125
H	-5.612	-4.956	2.768
C	-3.549	-4.265	1.171
H	-3.227	-4.673	2.135
H	-2.753	-3.607	0.803
H	-3.653	-5.101	0.467
C	-6.610	-2.165	0.155
O	-6.661	-1.126	0.822
N	-7.698	-2.677	-0.454
H	-7.657	-3.605	-0.859
C	-9.029	-2.152	-0.169
H	-9.695	-2.610	-0.911
C	-9.511	-2.555	1.211
H	-10.491	-2.104	1.413
H	-8.825	-2.181	1.984
C	-9.629	-4.065	1.312

O	-9.529	-4.796	0.332
N	-9.842	-4.532	2.559
H	-9.962	-3.92 0	3.352
H	-9.975	-5.524	2.692
C	-9.090	-0.645	-0.401
O	-9.684	0.124	0.357
N	-8.508	-0.214	-1.538
H	-8.070	-0.893	-2.147
C	-8.566	1.171	-1.944
H	-9.570	1.570	-1.767
H	-8.371	1.237	-3.019
C	-7.605	2.124	-1.250
O	-7.704	3.327	-1.457
N	-6.687	1.567	-0.423
H	-6.719	0.568	-0.225
C	-5.889	2.370	0.488
H	-6.119	3.419	0.265
C	-6.223	2.020	1.940
H	-5.88 0	0.988	2.098
H	-5.625	2.652	2.607
C	-7.718	2.134	2.252
H	-7.943	3.067	2.783
H	-8.294	2.155	1.318
C	-8.194	0.941	3.057
H	-7.875	0.979	4.101
H	-7.835	0.008	2.607
N	-9.691	0.86 0	3.031
H	-10.128	1.752	3.275
H	-10.048	0.161	3.685
H	-9.980	0.587	2.070
C	-4.426	2.053	0.223
O	-3.988	0.917	0.439
N	-3.656	3.029	-0.284
H	-4.021	3.968	-0.376
C	-2.246	2.761	-0.512
H	-2.181	1.758	-0.946
C	-1.604	3.713	-1.513
H	-2.245	3.732	-2.408
H	-0.658	3.245	-1.829
C	-1.315	5.126	-1.028
H	-2.232	5.634	-0.696
H	-0.666	5.074	-0.141
C	-0.654	5.947	-2.128
H	0.188	5.388	-2.568
H	-1.364	6.105	-2.949
C	-0.172	7.307	-1.672
H	0.069	7.964	-2.509
H	-0.905	7.809	-1.035
N	1.092	7.192	-0.861
H	1.866	6.837	-1.432
H	1.002	6.549	-0.058
H	1.383	8.100	-0.490
C	-1.533	2.749	0.843
O	-1.775	3.591	1.704
N	-0.638	1.750	0.986
H	-0.464	1.123	0.200

C	0.082	1.517	2.219
H	0.038	2.447	2.803
C	-0.549	0.388	3.067
H	0.083	0.291	3.966
C	-1.954	0.779	3.506
H	-2.340	0.073	4.249
H	-2.646	0.780	2.652
H	-1.969	1.779	3.953
C	-0.523	-0.951	2.330
H	-1.078	-0.849	1.378
H	0.514	-1.188	2.054
C	-1.097	-2.108	3.131
H	-0.913	-3.062	2.623
H	-0.635	-2.168	4.125
H	-2.180	-2.016	3.272
C	1.525	1.199	1.865
O	1.818	0.663	0.796
N	2.454	1.511	2.788
H	2.178	1.962	3.653
C	3.870	1.254	2.551
H	3.993	1.200	1.462
C	4.749	2.346	3.126
H	4.334	3.328	2.846
H	4.716	2.274	4.223
C	6.197	2.241	2.631
H	6.474	1.169	2.588
C	6.345	2.828	1.232
H	7.350	2.644	0.826
H	6.188	3.917	1.259
H	5.616	2.418	0.520
C	7.137	2.942	3.600
H	8.171	2.942	3.232
H	7.125	2.467	4.588
H	6.836	3.992	3.728
C	4.216	-0.110	3.157
O	4.485	-0.250	4.345
N	4.146	-1.142	2.287
H	4.161	-0.925	1.292
C	4.563	-2.464	2.692
H	3.955	-2.783	3.550
C	4.353	-3.418	1.520
H	3.299	-3.354	1.222
H	4.947	-3.061	0.665
C	4.711	-4.860	1.854
H	5.796	-4.966	1.992
H	4.242	-5.164	2.799
C	4.315	-5.800	0.745
O	4.569	-5.540	-0.439
N	3.683	-6.923	1.104
H	3.467	-7.134	2.066
H	3.424	-7.595	0.395
C	6.040	-2.458	3.088
O	6.883	-1.915	2.379
N	6.350	-3.141	4.202
H	5.641	-3.491	4.829
H	7.314	-3.182	4.500

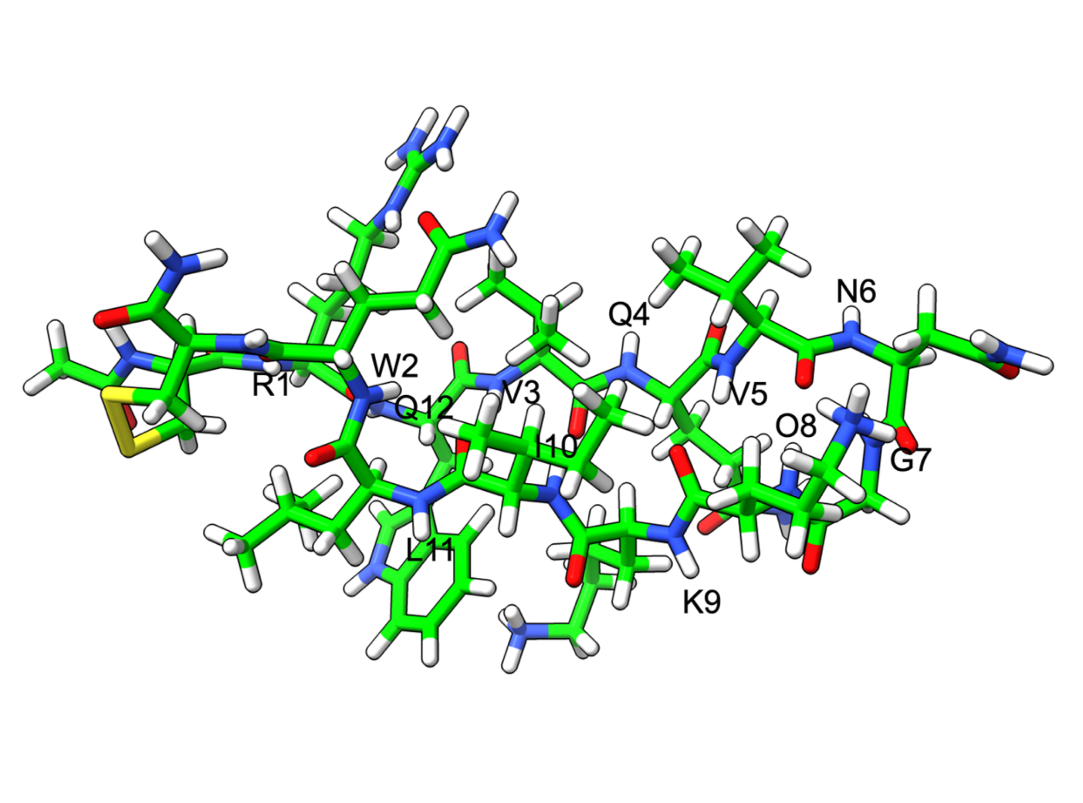

Figure S20. *Energy minimized structure of* ***cyc-1***. *All backbone amide protons are labelled*

Table S12. *Cartesian coordinates of the energy-minimized structure of* ***cyc-1***

Atom	x	y	z
C	-11.118	-0.569	-1.108
C	-12.558	-0.242	-1.412
O	-10.699	-1.720	-1.131
H	-12.737	0.816	-1.618
H	-12.882	-0.838	-2.268
H	-13.173	-0.536	-0.555
N	-10.350	0.497	-0.755
C	-8.946	0.412	-0.446
C	-8.112	0.165	-1.719
C	-8.593	-0.654	0.595
O	-8.626	-0.100	-2.796
S	-9.664	-0.755	2.076
H	-10.792	1.404	-0.707
H	-8.643	1.377	-0.040
H	-8.627	-1.623	0.098
H	-7.570	-0.479	0.927
N	-6.770	0.227	-1.540
C	-3.093	5.203	-4.209
N	-2.259	6.069	-3.636
C	-5.874	-0.170	-2.601
C	-4.575	-0.629	-1.973
C	-5.549	0.957	-3.597
O	-4.207	-0.177	-0.890
C	-4.706	2.079	-3.010
C	-4.436	3.151	-4.046
N	-3.610	4.210	-3.485
N	-3.387	5.315	-5.507
H	-6.395	0.552	-0.661
H	-6.318	-1.004	-3.144
H	-5.006	0.525	-4.438
H	-6.484	1.379	-3.965
H	-3.757	1.669	-2.665
H	-5.234	2.521	-2.165
H	-3.919	2.705	-4.896
H	-5.383	3.573	-4.382
H	-3.418	4.192	-2.494
H	-2.030	5.977	-2.657
H	-1.856	6.818	-4.181
H	-2.985	6.063	-6.053
H	-4.012	4.651	-5.942
N	-3.831	-1.483	-2.705
C	-2.433	-1.680	-2.392
C	-1.579	-0.627	-3.090
C	-1.960	-3.077	-2.813
O	-1.835	-0.227	-4.222
C	-2.518	-4.151	-1.946
C	-3.545	-5.013	-2.221
C	-2.080	-4.458	-0.615
C	-2.883	-5.528	-0.145

C	-1.080	-3.938	0.219
N	-3.767	-5.843	-1.145
C	-1.699	-5.566	1.921
C	-2.706	-6.089	1.122
C	-0.888	-4.502	1.476
H	-4.251	-1.986	-3.473
H	-2.300	-1.578	-1.315
H	-2.275	-3.258	-3.841
H	-0.872	-3.112	-2.765
H	-4.100	-5.038	-3.147
H	-0.468	-3.111	-0.110
H	-1.533	-5.982	2.904
H	-3.330	-6.900	1.467
H	-0.108	-4.120	2.119
H	-4.469	-6.568	-1.099
N	-0.505	-0.266	-2.360
C	0.633	0.444	-2.885
C	1.877	-0.290	-2.405
C	0.690	1.912	-2.402
O	1.808	-1.127	-1.501
C	-0.613	2.625	-2.731
C	0.997	1.993	-0.911
H	-0.498	-0.509	-1.380
H	0.600	0.424	-3.974
H	1.494	2.413	-2.940
H	-0.560	3.658	-2.386
H	-0.773	2.610	-3.809
H	-1.440	2.118	-2.234
H	1.934	1.475	-0.704
H	1.087	3.038	-0.615
H	0.190	1.524	-0.348
N	3.052	0.097	-2.930
C	4.303	-0.307	-2.305
C	5.250	0.872	-2.463
C	4.858	-1.591	-2.906
O	5.455	1.362	-3.568
C	6.070	-2.119	-2.146
C	6.369	-3.555	-2.526
N	7.644	-3.821	-2.879
O	5.499	-4.420	-2.487
H	3.067	0.670	-3.761
H	4.124	-0.469	-1.242
H	4.077	-2.351	-2.888
H	5.144	-1.402	-3.941
H	6.936	-1.501	-2.383
H	5.873	-2.065	-1.075
H	8.331	-3.080	-2.888
H	7.912	-4.761	-3.134
N	5.791	1.331	-1.309
C	6.530	2.580	-1.270
C	7.806	2.315	-0.502
C	5.730	3.706	-0.602
O	7.757	1.987	0.693
C	6.571	4.975	-0.539
C	4.424	3.948	-1.343
H	5.678	0.795	-0.461
H	6.782	2.877	-2.288

H	5.494	3.400	0.417
H	5.996	5.769	-0.063
H	6.845	5.280	-1.549
H	7.474	4.784	0.04 0
H	3.871	4.750	-0.854
H	3.826	3.036	-1.333
H	4.638	4.230	-2.374
N	8.967	2.388	-1.169
C	10.244	2.125	-0.514
C	10.175	0.821	0.293
C	10.716	3.302	0.319
O	10.494	0.751	1.479
C	12.138	3.091	0.799
N	12.457	3.715	1.952
O	12.933	2.414	0.158
H	8.957	2.631	-2.149
H	10.982	1.975	-1.302
H	10.675	4.207	-0.287
H	10.060	3.418	1.181
H	11.764	4.268	2.436
H	13.389	3.629	2.332
N	9.767	-0.251	-0.419
C	9.728	-1.588	0.129
C	8.478	-1.975	0.902
O	8.348	-3.120	1.314
H	9.476	-0.113	-1.376
H	9.833	-2.287	-0.700
H	10.587	-1.711	0.788
N	7.587	-0.983	1.145
C	6.550	-1.131	2.153
C	5.210	-1.245	1.447
C	6.589	0.053	3.108
O	4.667	-0.261	0.933
C	7.881	0.053	3.924
C	8.299	1.418	4.426
N	8.882	2.226	3.305
H	7.645	-0.125	0.616
H	6.732	-2.046	2.716
H	6.53 0	0.977	2.533
H	5.737	-0.004	3.785
H	7.741	-0.600	4.786
H	8.683	-0.352	3.307
H	7.428	1.936	4.828
H	9.044	1.301	5.213
H	9.787	1.853	3.056
H	8.984	3.187	3.600
H	8.268	2.183	2.504
N	4.707	-2.490	1.374
C	3.459	-2.803	0.689
C	2.315	-2.571	1.683
C	3.491	-4.229	0.168
O	2.231	-3.238	2.711
C	2.386	-4.475	-0.848
C	2.425	-5.873	-1.452
C	2.031	-6.989	-0.508
N	0.558	-6.942	-0.196
H	5.217	-3.243	1.814

H	3.338	-2.122	-0.153
H	4.455	-4.411	-0.306
H	3.368	-4.918	1.004
H	1.424	-4.338	-0.353
H	2.476	-3.743	-1.650
H	1.743	-5.893	-2.302
H	3.435	-6.066	-1.814
H	2.594	-6.886	0.419
H	2.27 0	-7.948	-0.968
H	0.323	-7.695	0.434
H	0.030	-7.041	-1.051
H	0.334	-6.058	0.238
N	1.480	-1.560	1.359
C	0.530	-0.988	2.291
C	-0.847	-0.999	1.652
C	0.918	0.467	2.633
O	-0.999	-0.580	0.501
C	2.278	0.534	3.329
C	-0.163	1.155	3.459
C	2.948	1.889	3.165
H	1.520	-1.185	0.422
H	0.512	-1.584	3.204
H	1.006	1.011	1.693
H	2.137	0.34 0	4.392
H	2.928	-0.235	2.912
H	-1.117	1.090	2.936
H	0.101	2.203	3.602
H	-0.246	0.665	4.429
H	3.91 0	1.884	3.678
H	2.312	2.663	3.595
H	3.102	2.092	2.105
N	-1.868	-1.442	2.408
C	-3.251	-1.348	1.955
C	-3.793	0.011	2.408
C	-4.126	-2.473	2.465
O	-4.413	0.173	3.455
C	-5.500	-2.439	1.783
C	-5.460	-3.091	0.407
C	-6.556	-3.087	2.664
H	-1.672	-1.850	3.311

H	-3.258	-1.375	0.865
H	-3.644	-3.427	2.252
H	-4.256	-2.368	3.542
H	-5.777	-1.394	1.647
H	-4.696	-2.608	-0.202
H	-6.432	-2.982	-0.075
H	-5.224	-4.150	0.513
H	-7.522	-3.051	2.160
H	-6.620	-2.549	3.610
H	-6.283	-4.125	2.854
N	-3.473	1.039	1.585
C	-4.025	2.346	1.857
C	-5.547	2.291	1.817
C	-3.543	3.346	0.816
O	-6.150	1.752	0.895
C	-2.050	3.603	0.912
C	-1.587	4.657	-0.061
N	-0.396	5.215	0.198
O	-2.257	4.969	-1.053
H	-2.860	0.894	0.796
H	-3.703	2.673	2.846
H	-3.768	2.955	-0.176
H	-4.075	4.287	0.955
H	-1.816	3.933	1.924
H	-1.516	2.674	0.709
H	0.129	4.929	1.012
H	-0.024	5.921	-0.421
N	-6.191	2.939	2.821
C	-7.637	2.873	2.865
C	-8.274	4.055	3.578
C	-8.142	1.549	3.431
O	-9.37 0	3.928	4.112
S	-9.822	1.194	2.771
H	-5.675	3.454	3.520
H	-7.979	2.916	1.831
H	-7.462	0.748	3.141
H	-8.185	1.612	4.518
N	-7.586	5.211	3.540
H	-8.012	6.04 0	3.929
H	-6.722	5.302	3.028

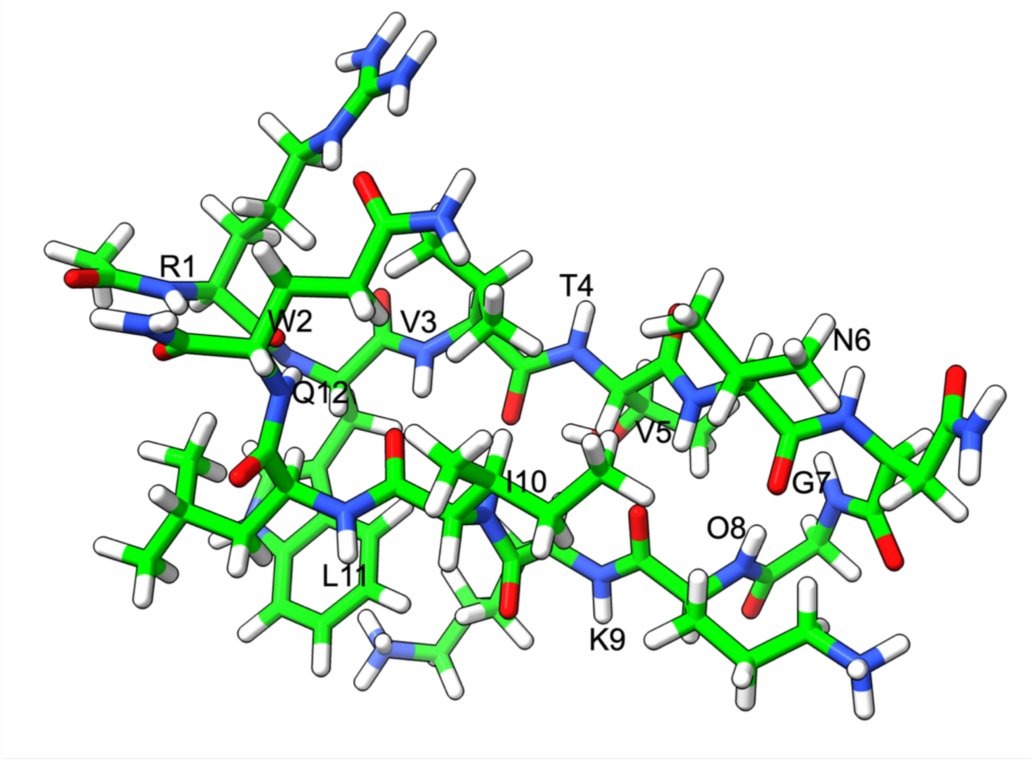

Figure S21. *Energy minimized structure of* ***2***. *All backbone amide protons are labelled.*

Table S13. *Cartesian coordinates of the energy-minimized structure of* ***2.***

Atom	x	y	z
H	9.877	-1.218	-2.347
C	10.150	-0.582	-1.499
H	11.213	-0.704	-1.290
H	9.968	0.458	-1.795
C	9.382	-0.918	-0.244
O	9.955	-1.127	0.819
N	8.022	-0.954	-0.337
H	7.519	-1.080	0.545
C	7.246	-0.740	-1.538
H	7.748	0.003	-2.172
C	7.026	-2.018	-2.374
H	6.488	-1.740	-3.292
H	8.007	-2.402	-2.684
C	6.256	-3.097	-1.633
H	5.268	-2.718	-1.338
H	6.784	-3.364	-0.705
C	6.045	-4.342	-2.469
H	5.520	-4.079	-3.401
H	7.009	-4.799	-2.739
N	5.238	-5.275	-1.696
H	4.965	-5.019	-0.745
C	4.743	-6.418	-2.163
N	5.037	-6.828	-3.399
H	5.716	-6.343	-3.964
H	4.686	-7.707	-3.746
N	3.933	-7.138	-1.388
H	3.716	-6.789	-0.454
H	3.594	-8.039	-1.684
C	5.874	-0.219	-1.154
O	5.293	-0.649	-0.158
N	5.310	0.657	-2.009
H	5.789	0.862	-2.878
C	3.882	0.890	-1.955
H	3.602	1.025	-0.901
C	3.504	2.152	-2.740
H	2.409	2.156	-2.852
H	3.916	2.076	-3.756
C	3.953	3.399	-2.059
C	5.106	4.107	-2.270
H	5.890	3.940	-2.999
N	5.189	5.156	-1.383
H	5.942	5.827	-1.345
C	4.083	5.146	-0.571
C	3.718	5.990	0.479
H	4.347	6.827	0.776
C	2.528	5.713	1.142
H	2.225	6.338	1.979
C	1.712	4.628	0.758
H	0.791	4.433	1.308
C	2.077	3.797	-0.295
H	1.450	2.947	-0.570
C	3.279	4.046	-0.970
C	3.136	-0.319	-2.523

O	3.547	-0.934	-3.502
N	1.988	-0.574	-1.868
H	1.768	-0.015	-1.045
C	0.971	-1.488	-2.322
H	1.184	-1.724	-3.375
C	0.960	-2.804	-1.514
H	0.274	-3.496	-2.026
C	2.345	-3.436	-1.520
H	2.747	-3.529	-2.536
H	2.317	-4.433	-1.064
H	3.047	-2.818	-0.938
C	0.466	-2.588	-0.088
H	-0.587	-2.273	-0.055
H	0.548	-3.513	0.495
H	1.065	-1.812	0.413
C	-0.361	-0.746	-2.239
O	-0.405	0.455	-1.933
N	-1.479	-1.448	-2.476
H	-1.448	-2.426	-2.748
C	-2.792	-0.932	-2.109
H	-2.672	-0.239	-1.266
C	-3.437	-0.163	-3.281
H	-3.249	-0.773	-4.185
C	-4.929	0.047	-3.138
H	-5.279	0.686	-3.954
H	-5.468	-0.906	-3.196
H	-5.167	0.538	-2.185
O	-2.843	1.113	-3.412
H	-1.900	1.042	-3.194
C	-3.642	-2.115	-1.686
O	-3.575	-3.184	-2.288
N	-4.459	-1.870	-0.640
H	-4.463	-0.942	-0.209
C	-5.480	-2.811	-0.215
H	-5.594	-3.554	-1.016
C	-5.116	-3.529	1.096
H	-4.923	-2.740	1.840
C	-6.277	-4.389	1.581
H	-7.145	-3.789	1.882
H	-5.975	-4.980	2.452
H	-6.604	-5.091	0.801
C	-3.858	-4.365	0.919
H	-3.012	-3.767	0.564
H	-4.029	-5.173	0.195
H	-3.570	-4.822	1.873
C	-6.754	-2.008	-0.029
O	-6.755	-0.998	0.682
N	-7.850	-2.438	-0.685
H	-7.830	-3.351	-1.126
C	-9.173	-1.879	-0.429
H	-9.831	-2.326	-1.186
C	-9.702	-2.265	0.940
H	-9.042	-1.881	1.730
H	-10.678	-1.793	1.101
C	-9.849	-3.770	1.051
O	-9.761	-4.512	0.077
N	-10.080	-4.226	2.300

H	-10.189	-3.607	3.089
H	-10.236	-5.214	2.437
C	-9.220	-0.370	-0.652
O	-9.942	0.355	0.024
N	-8.463	0.092	-1.677
H	-7.932	-0.571	-2.226
C	-8.447	1.486	-2.047
H	-8.249	1.578	-3.119
H	-9.436	1.921	-1.863
C	-7.447	2.375	-1.321
O	-7.378	3.563	-1.611
N	-6.709	1.788	-0.348
H	-6.768	0.782	-0.188
C	-5.764	2.539	0.457
H	-5.983	3.602	0.291
C	-5.849	2.190	1.945
H	-5.048	2.741	2.455
H	-5.608	1.122	2.055
C	-7.179	2.492	2.632
C	-4.359	2.186	-0.006
O	-3.972	1.015	0.056
N	-3.580	3.185	-0.464
H	-3.893	4.141	-0.350
C	-2.176	2.915	-0.722
H	-2.145	1.933	-1.199
C	-1.544	3.897	-1.699
H	-0.579	3.464	-2.007
H	-2.172	3.913	-2.601
C	-1.306	5.314	-1.201
H	-2.239	5.781	-0.852
H	-0.640	5.284	-0.326
C	-0.707	6.169	-2.311
H	0.121	5.630	-2.799
H	-1.457	6.330	-3.095
C	-0.217	7.525	-1.852
H	-0.085	8.224	-2.680
H	-0.888	7.980	-1.118
N	1.130	7.410	-1.191
H	1.439	8.305	-0.803
H	1.151	6.723	-0.420
H	1.843	7.113	-1.864
C	-1.445	2.837	0.620
O	-1.687	3.618	1.536
N	-0.542	1.833	0.693
H	-0.448	1.206	-0.107
C	0.074	1.397	1.933
H	-0.116	2.170	2.690
C	-0.547	0.054	2.364
H	-0.520	-0.574	1.457
C	0.264	-0.636	3.453
H	-0.231	-1.560	3.773
H	1.274	-0.906	3.115
H	0.364	0.010	4.336
C	-2.011	0.236	2.769
H	-2.053	0.545	3.825
H	-2.465	1.049	2.189
C	-2.837	-1.018	2.539

H	-3.888	-0.863	2.817
H	-2.466	-1.881	3.108
H	-2.816	-1.291	1.474
C	1.558	1.218	1.679
O	1.939	0.546	0.715
N	2.413	1.784	2.545
H	2.057	2.326	3.323
C	3.858	1.659	2.381
H	4.019	1.431	1.320
C	4.559	2.954	2.745
H	4.114	3.755	2.134
H	4.347	3.190	3.799
C	6.074	2.937	2.528
H	6.518	2.258	3.274
C	6.458	2.429	1.142
H	7.532	2.571	0.963
H	6.249	1.359	1.027
H	5.914	2.976	0.354
C	6.625	4.338	2.757
H	7.719	4.351	2.697
H	6.240	5.026	1.989
H	6.333	4.734	3.737
C	4.337	0.479	3.227
O	4.586	0.590	4.423
N	4.404	-0.694	2.555
H	4.423	-0.652	1.536
C	5.034	-1.837	3.170

H	4.584	-1.984	4.163
C	4.820	-3.085	2.318
H	5.395	-3.913	2.750
H	5.228	-2.902	1.312
C	3.349	-3.458	2.241
H	2.896	-3.442	3.242
H	2.794	-2.711	1.651
C	3.097	-4.800	1.604
O	3.902	-5.329	0.826
N	1.922	-5.374	1.905
H	1.289	-4.973	2.582
H	1.681	-6.265	1.495
C	6.534	-1.587	3.344
O	7.167	-0.875	2.573
N	7.101	-2.246	4.373
H	6.563	-2.787	5.033
H	8.098	-2.170	4.514
C	-8.294	1.620	2.093
N	-9.494	1.573	2.993
H	-7.439	3.554	2.527
H	-7.050	2.302	3.706
H	-7.971	0.582	1.967
H	-8.678	1.962	1.133
H	-10.221	1.007	2.545
H	-9.286	1.156	3.904
H	-9.887	2.503	3.162

2.4. Orientation of the 4th residue side chain

As shown above, Q4T mutation has a significant influence on the extent of hairpin folding (52% for **1** and 79% for **2**). The orientation of the 4th residue side chain sheds light on how the structures of **1** and **2** differ, and hence explains the significant differences in stability. ROESY NMR and energy-minimized structures were inspected as depicted below.

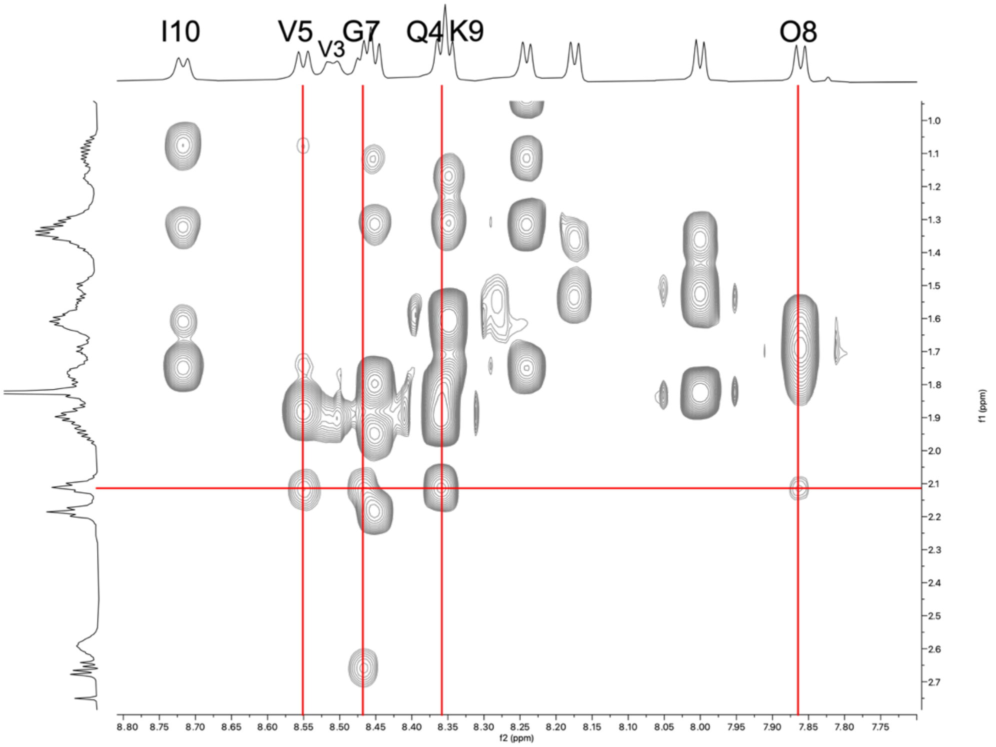

Figure S22. *A zoom-in of ROESY spectrum of* ***1****. Cross peaks of Q4's* H_γ *(marked by the horizontal line, δ = 2.12 ppm) with amide protons of V5, G7, O8 (marked by vertical lines) indicate that Q4 sidechain faces towards the turn.*

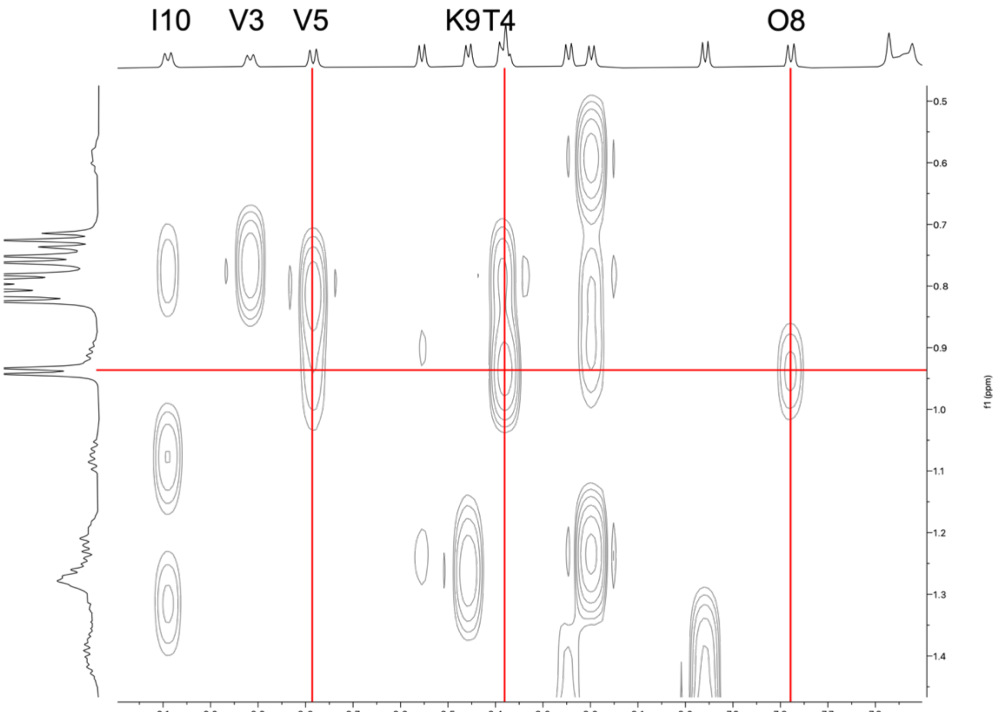

Figure S23. *A zoom-in of the ROESY spectrum of* ***2***. *Cross peaks of T4's H_γ (marked by the horizontal line, δ = 0.94 ppm) with amide protons of V5, O8 (marked by vertical lines) indicate that the T4 methyl group faces towards the turn and the hydroxyl group faces towards the termini.*

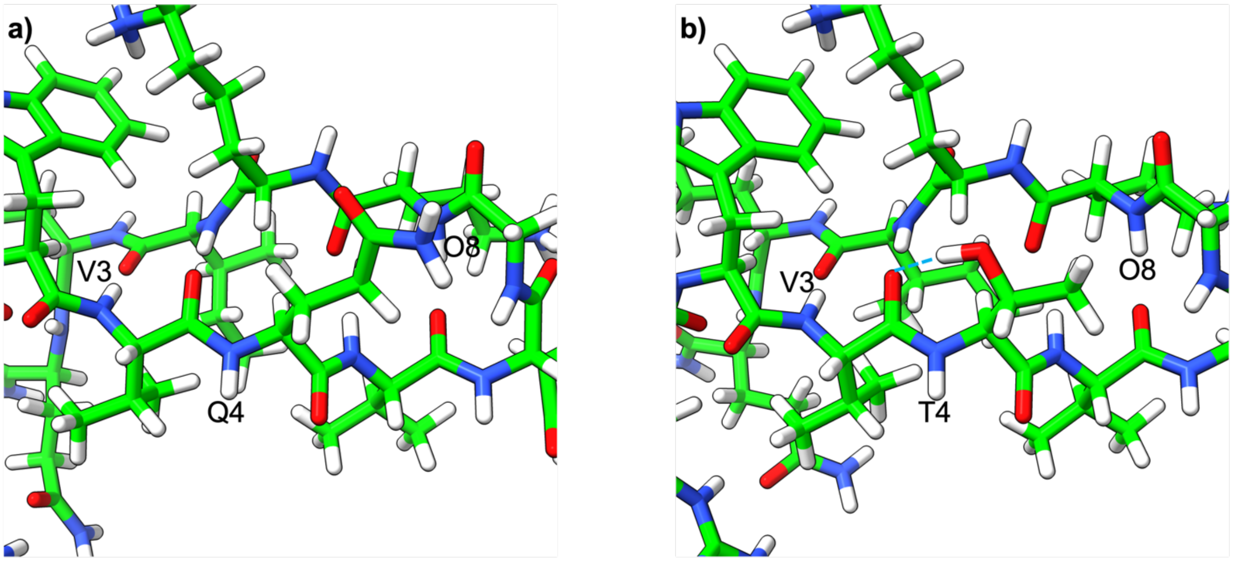

Figure S24. *Zoom-ins of energy minimized structure of a) peptide* ***1*** *and b) peptide* ***2***. *In b), T4's hydroxyl group forms a hydrogen bond with V3 carbonyl group (highlighted by a dashed blue line).*

3. NMR titration with anions

3.1. Peptide 1

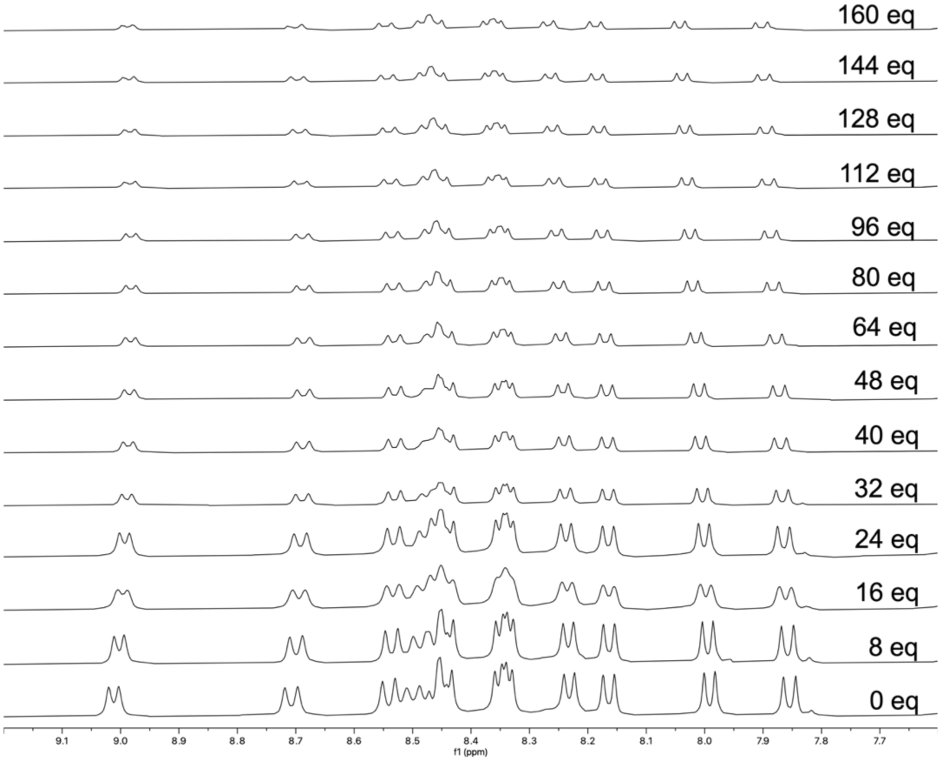

Figure S25. *Stack of NMR spectra showing the amide proton region of **1** as a function of NaCl.*

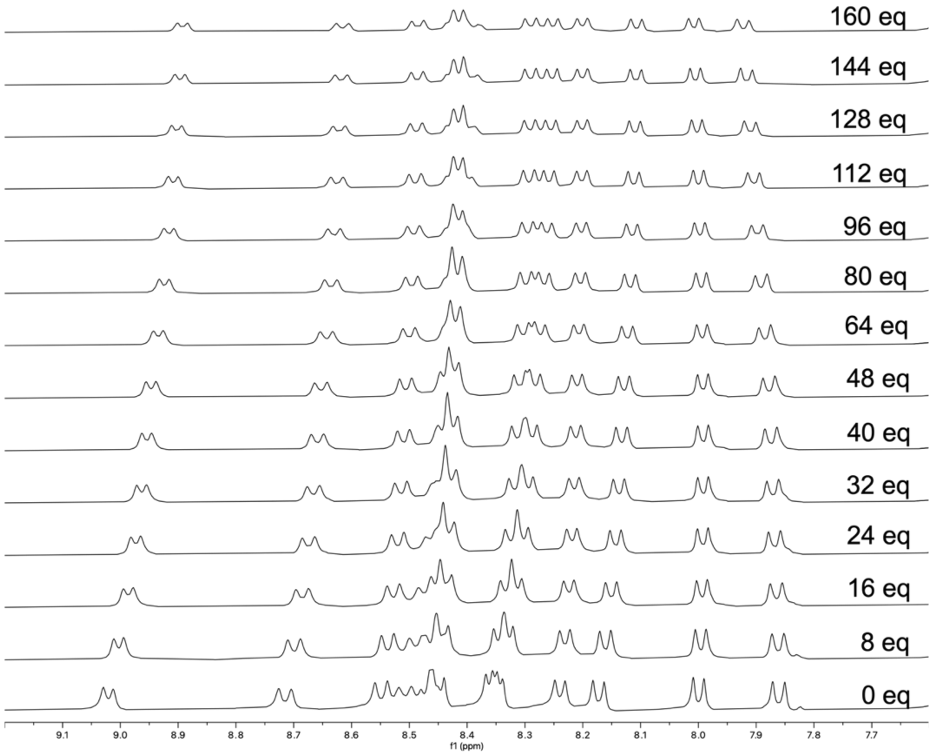

Figure S26. *Stack of NMR spectra showing the amide proton region of **1** as a function of NaI*

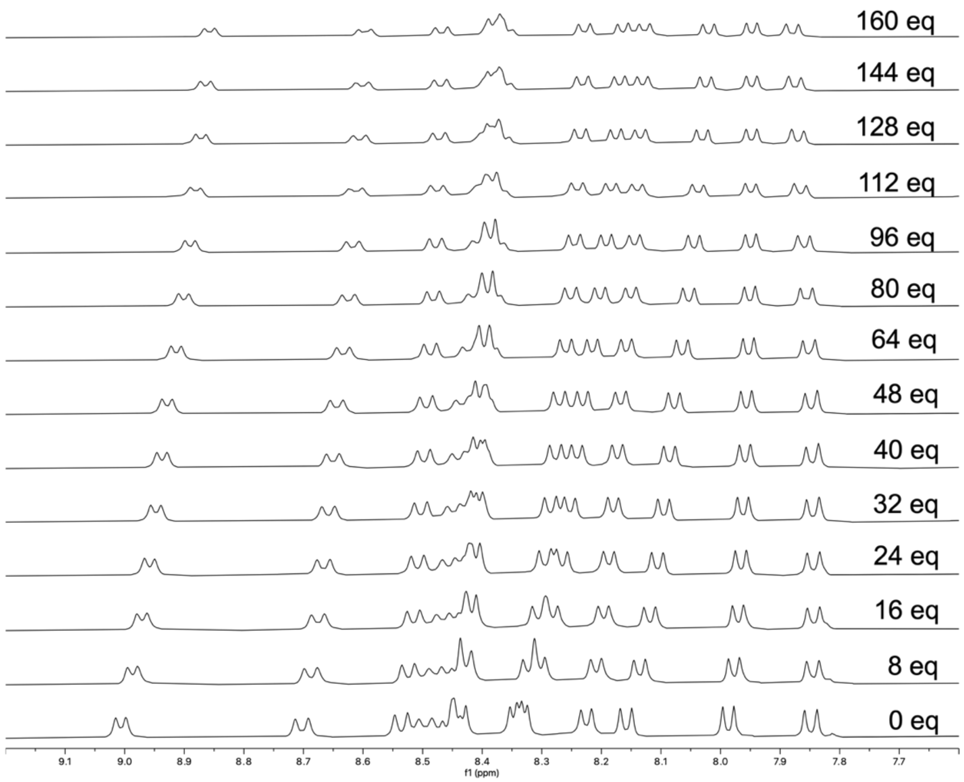

Figure S27. *Stack of NMR spectra showing the amide proton region of **1** as a function of $NaClO_4$*

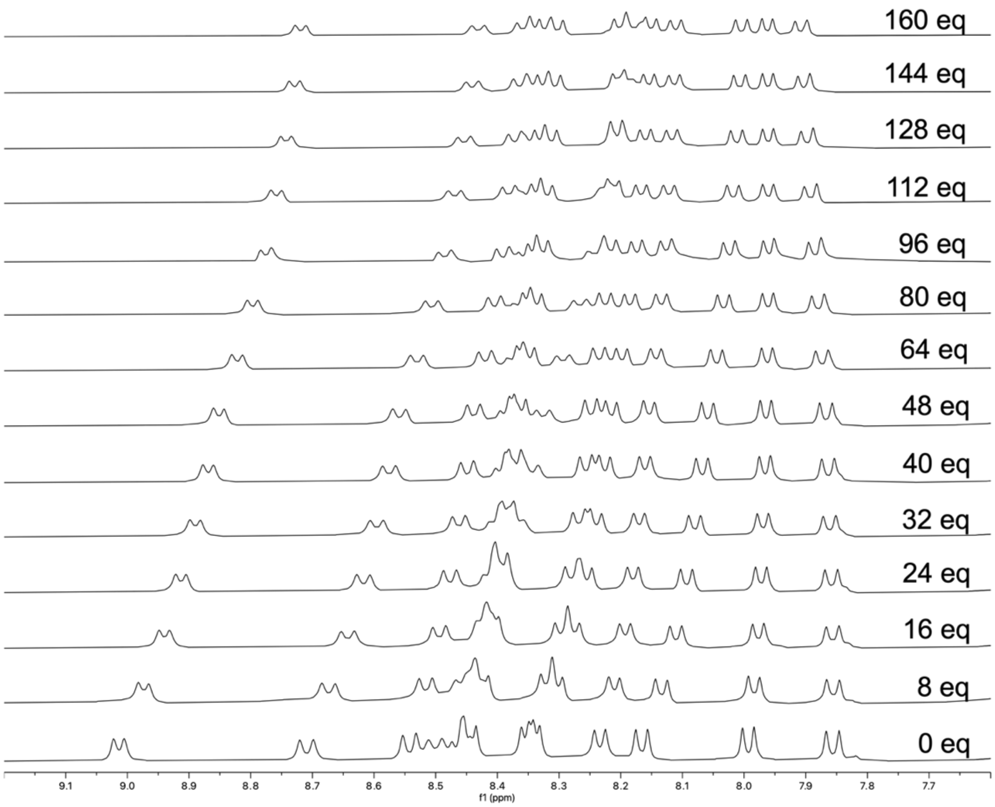

Figure S28. *Stack of NMR spectra showing the amide proton region of **1** as a function of $NaReO_4$*

3.2. Peptide 2

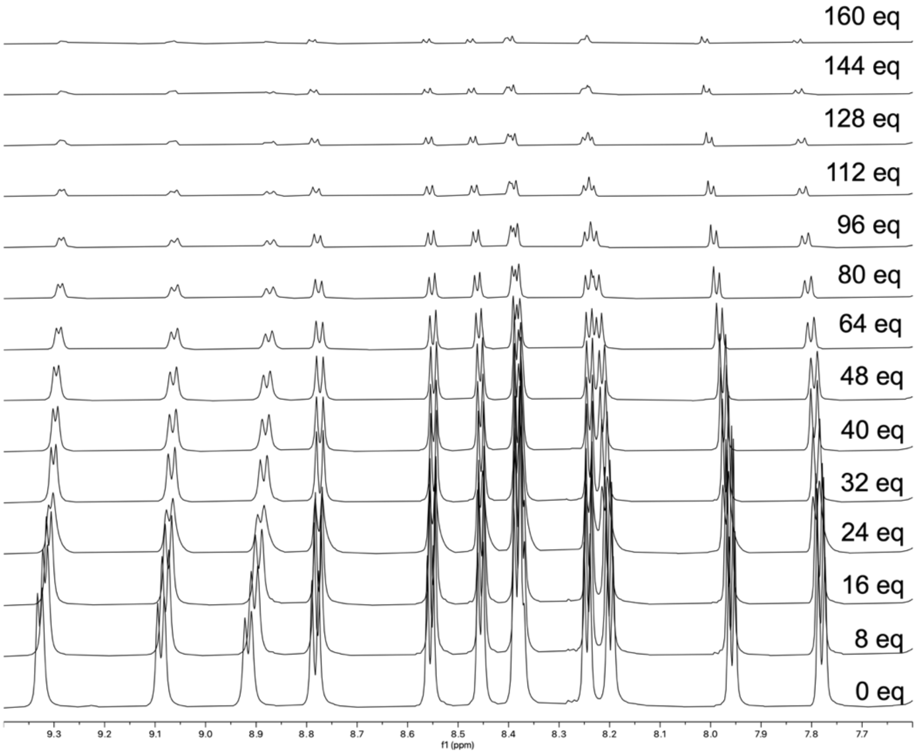

Figure S29. *Stack of NMR spectra showing the amide proton region of **3** as a function of NaCl*

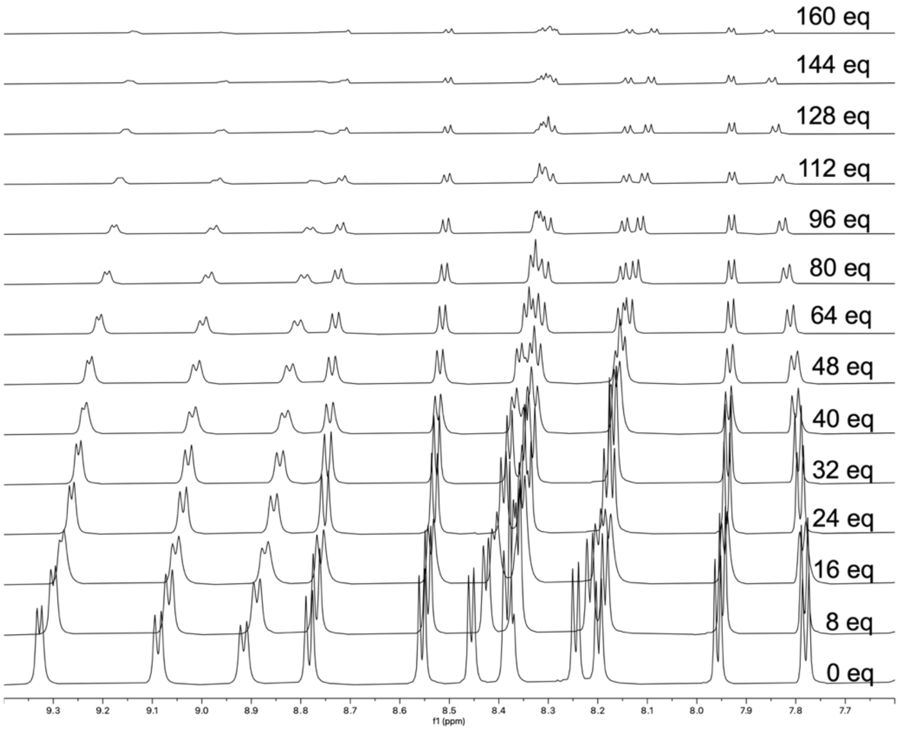

Figure S30. *Stack of NMR spectra showing the amide proton region of* ***3*** *as a function of* $NaClO_4$

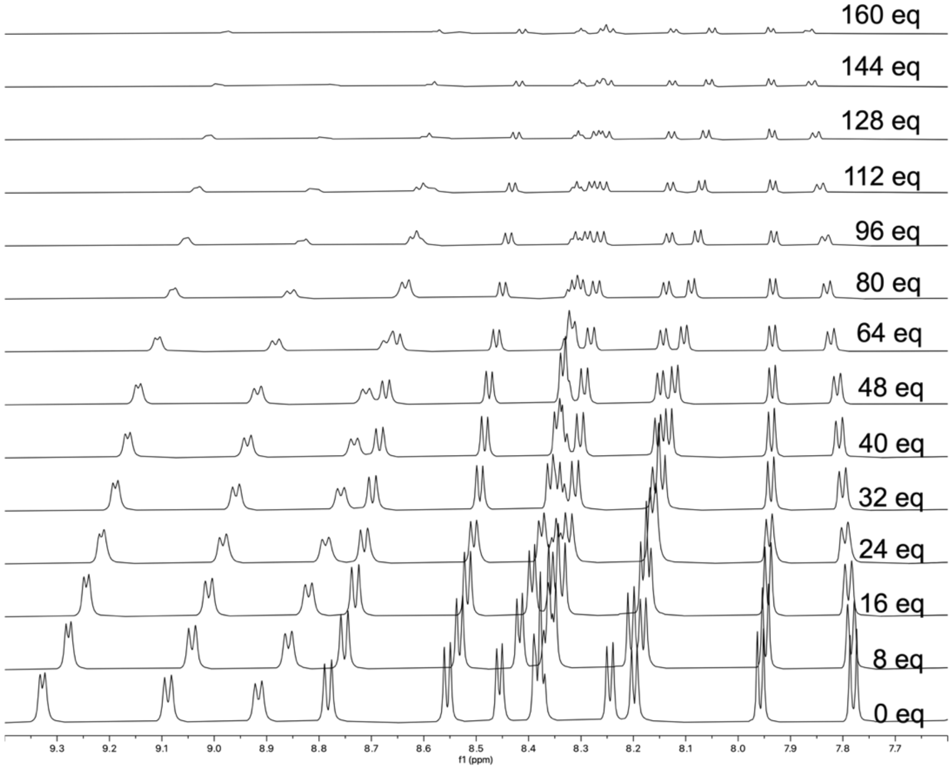

Figure S31*. Stack of NMR spectra showing the amide proton region of* ***3*** *as a function of* $NaReO_4$

3.3. Peptide cyc-1

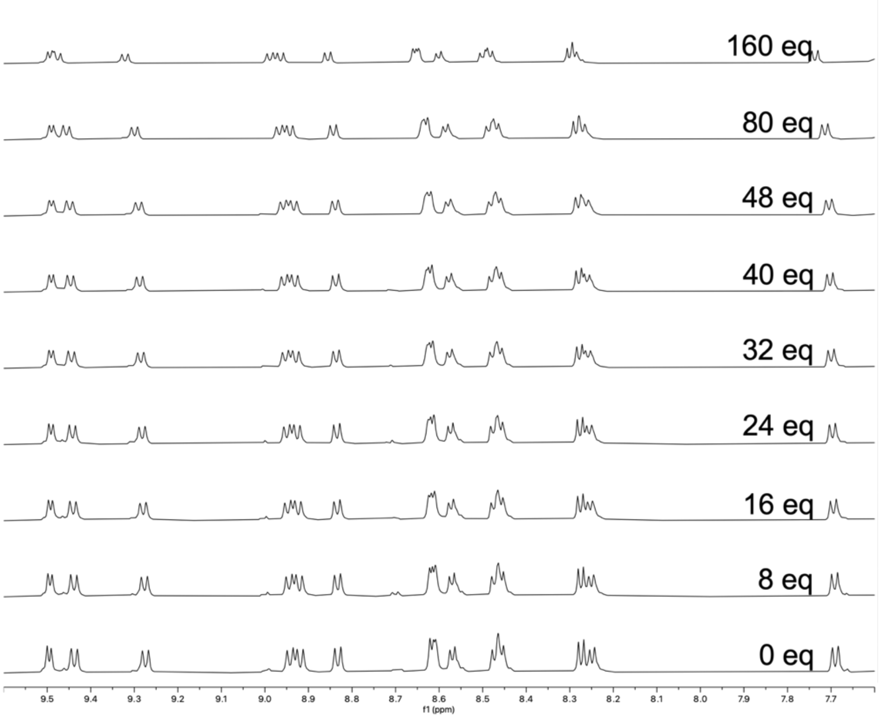

Figure S32. *Stack of NMR spectra showing the amide proton region of* ***cyc-1*** *as a function of NaCl*

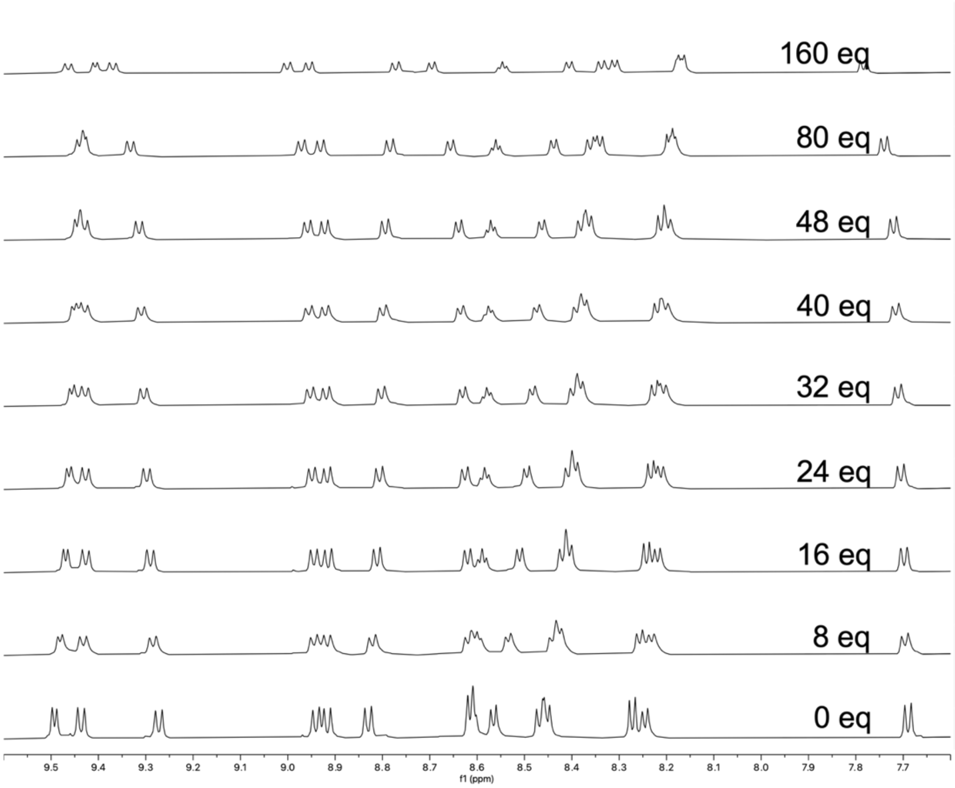

Figure S33. *Stack of NMR spectra showing the amide proton region of* ***cyc-1*** *as a function of* $NaClO_4$

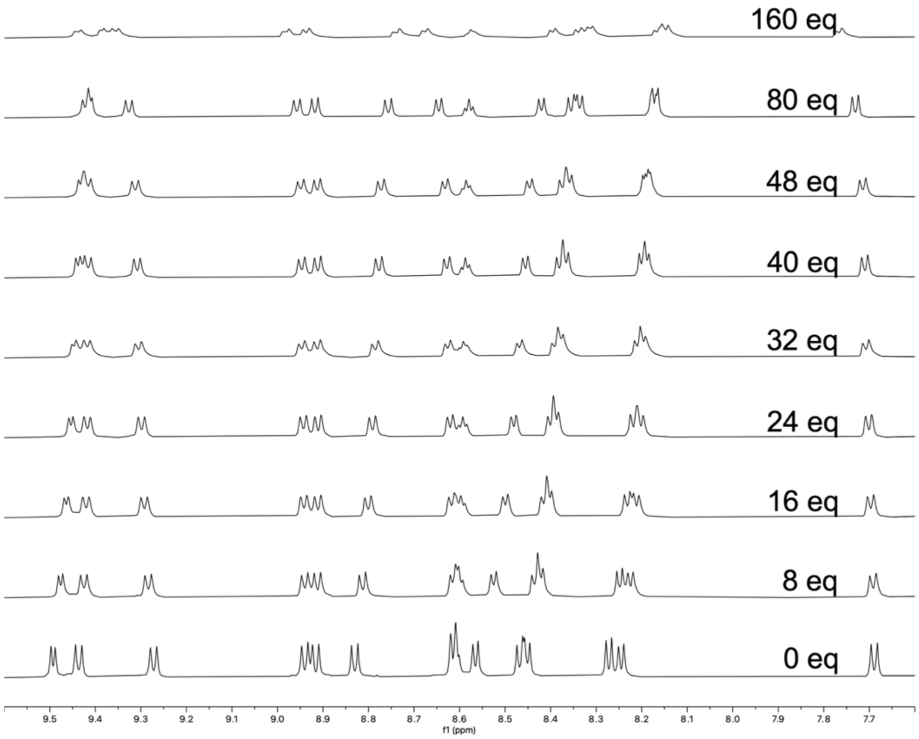

Figure S34. *Stack of NMR spectra showing the amide proton region of **cyc-1** as a function of* $NaReO_4$

3.4. Peptide cyc-2

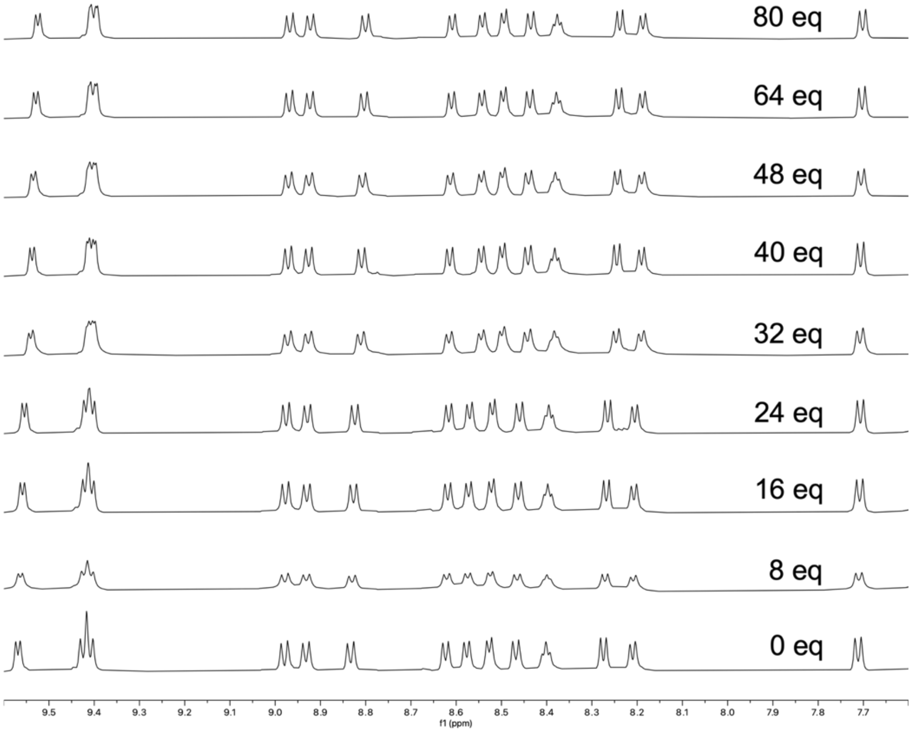

Figure S35. *Stack of NMR spectra showing the amide proton region of **cyc-2** as a function of NaCl*

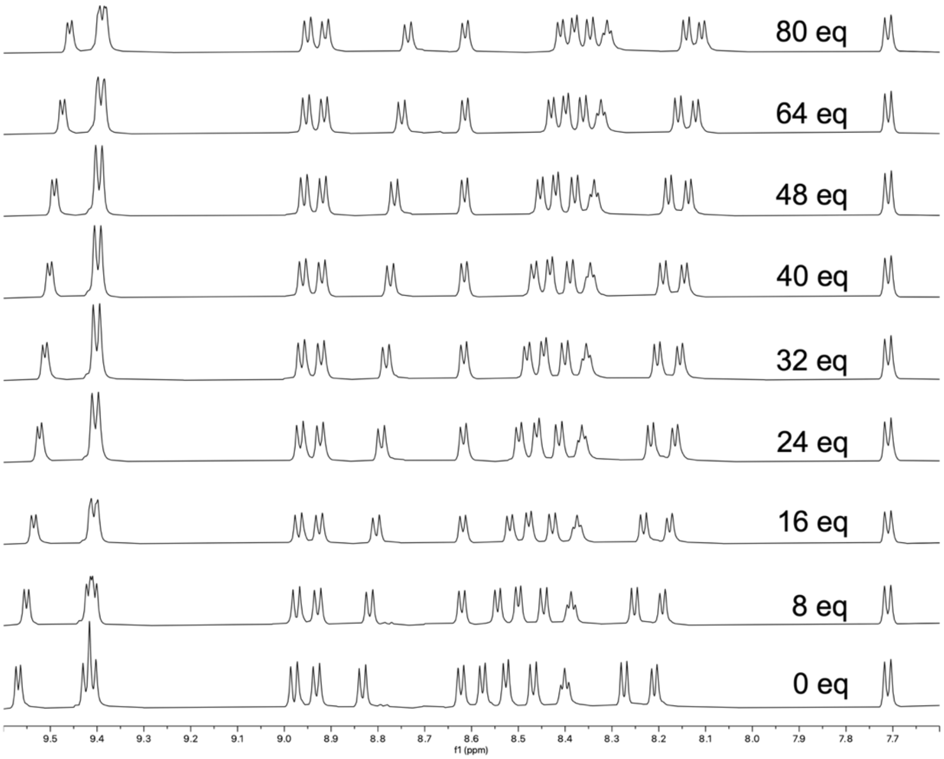

Figure S36. *Stack of NMR spectra showing the amide proton region of* ***cyc-2*** *as a function of* $NaClO_4$

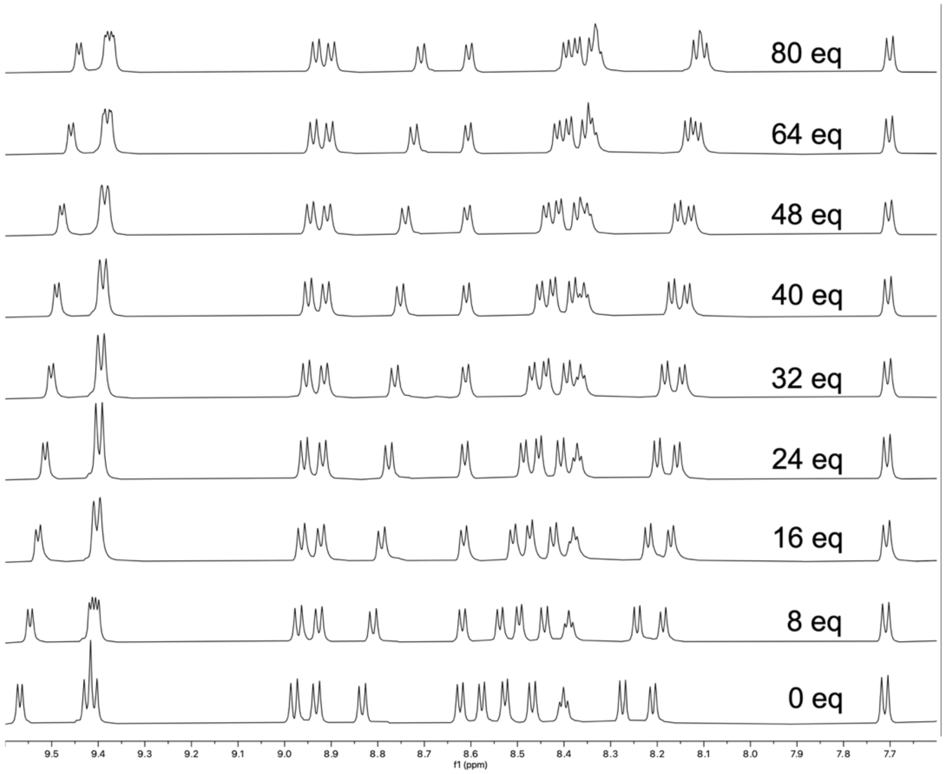

Figure S37. *Stack of NMR spectra showing the amide proton region of* ***cyc-2*** *as a function of* $NaReO_4$

4. Binding analysis and fitting

4.1. Anion affinity

All amide proton peaks were monitored in each titration experiment (0 eq of salt to 160 eq (650 mM) salt). To correct for ionic strength effects, the *N*-terminal acetyl proton was employed as an internal reference (see below).

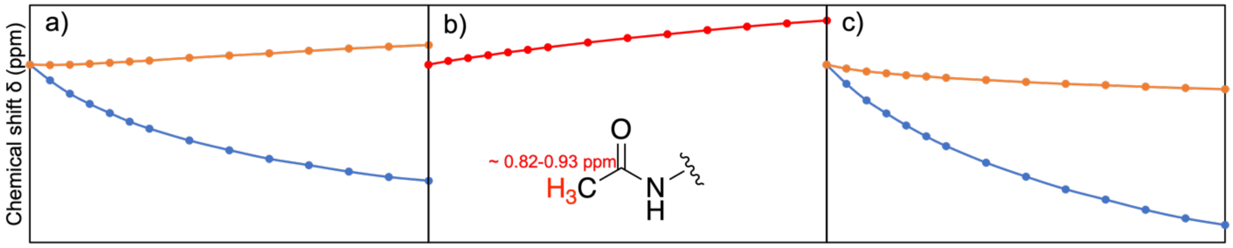

Figure S38. *Representative N-terminal acetyl subtraction. a) Representative raw data, amide proton chemical shifts as a function of salt. b) N-terminal acetyl proton (of the same peptide) chemical shifts as a function of salt. c) Referenced data, amide proton chemical shifts as a function of salts after subtraction.*

In order to evaluate the effects of anions on the peptides, chemical shift changes at 48 eq (230 mM) were chosen as a threshold. Among the four peptides, **1** and **cyc-1** was selected as examples to assess the unfolding effects of anions.

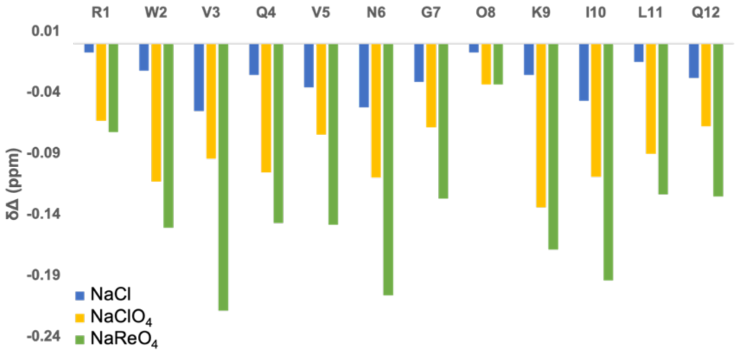

Figure S39. *Amide protons' shifts change (Δδ, ppm, and referenced) of **1** in the presence of 230 mM salts*

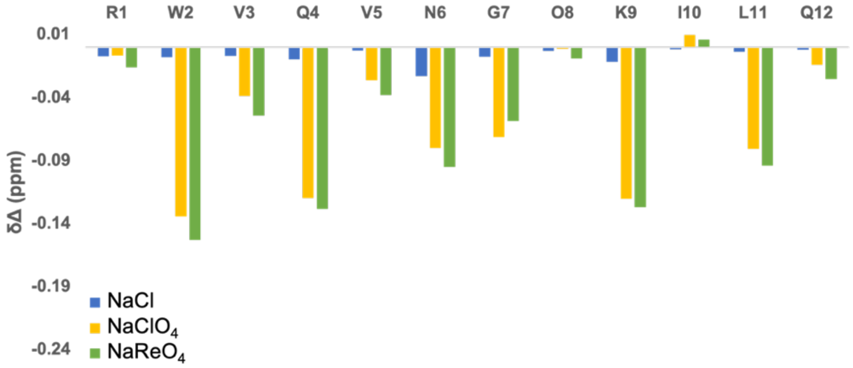

Figure S40. *Amide protons' shifts change (Δδ, ppm, and referenced) of **cyc-1** (cannot be unfolded) in the presence of 230 mM salts.*

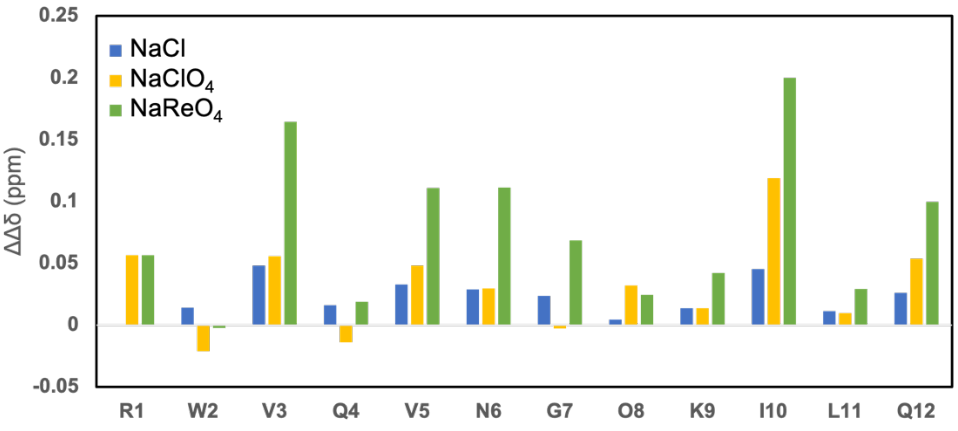

Figure S41. *Difference between of* ***cyc-1*** *and* ***1*** *from Figure S40 and S39 (ΔΔδ, ppm). There are significant differences at those intramolecular hydrogen bonded amide protons*

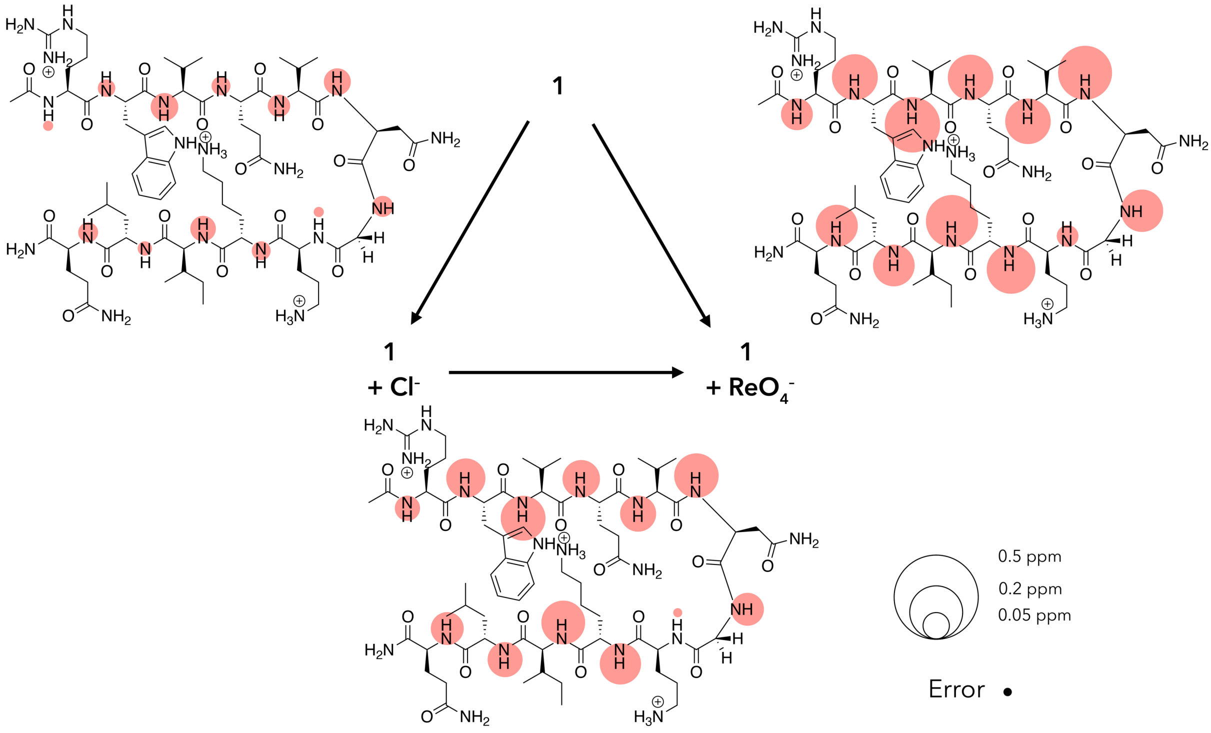

Figure S42. *Signal shifts of the mainchain amide groups of recipient* ***1*** *for the addition of 230 mM Cl⁻ (left) and ReO_4^- (right). In each, the effect of adding A⁻ to recipient* ***1*** *is defined by: $\Delta\delta = \delta_{1A^-} - \delta_1$. All signals moved upfield (red). At the base of the triangle is the difference of two sides. Where shifts are small the circle is shown above or below the amide H atom. A scale and error bubble are shown lower center.*

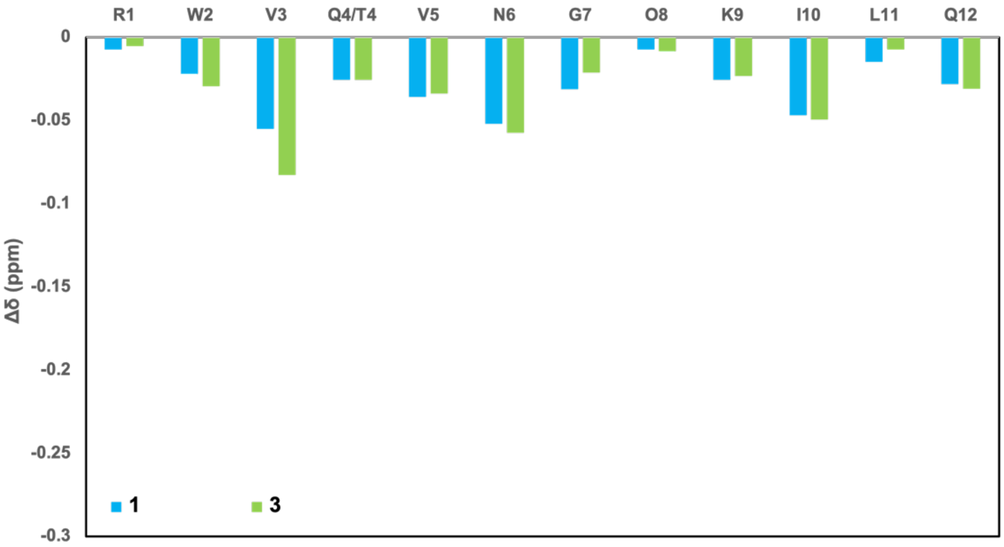

Figure S43. *Referenced amide proton signal shift changes (Δδ, ppm) of the two peptides in the presence of 230 mM NaCl. The 4th residue was glutamine for **1**, threonine for **2***

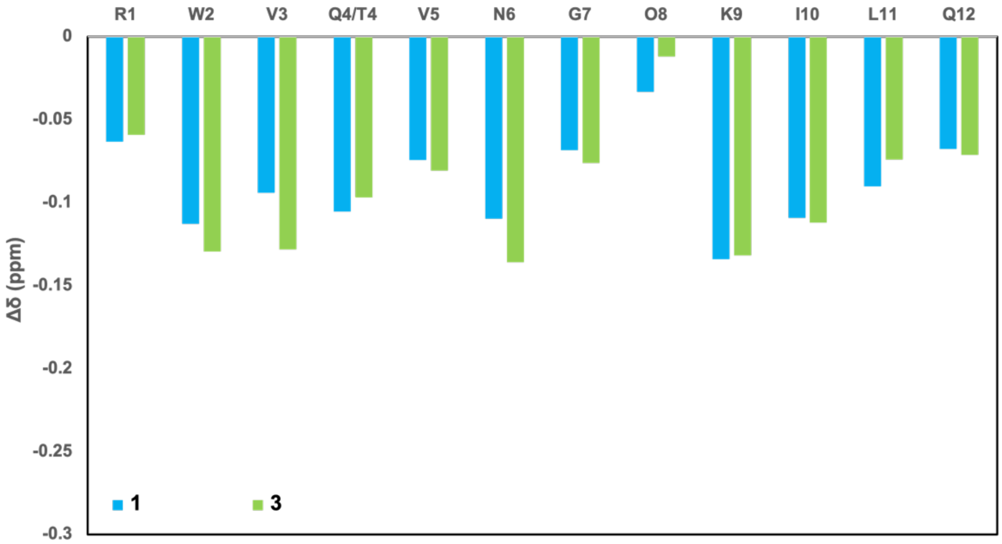

Figure S44. *Referenced amide proton signal shift changes (Δδ, ppm) of the four peptides in the presence of 230 mM $NaClO_4$. The 4th residue was glutamine for **1**, threonine for **2***

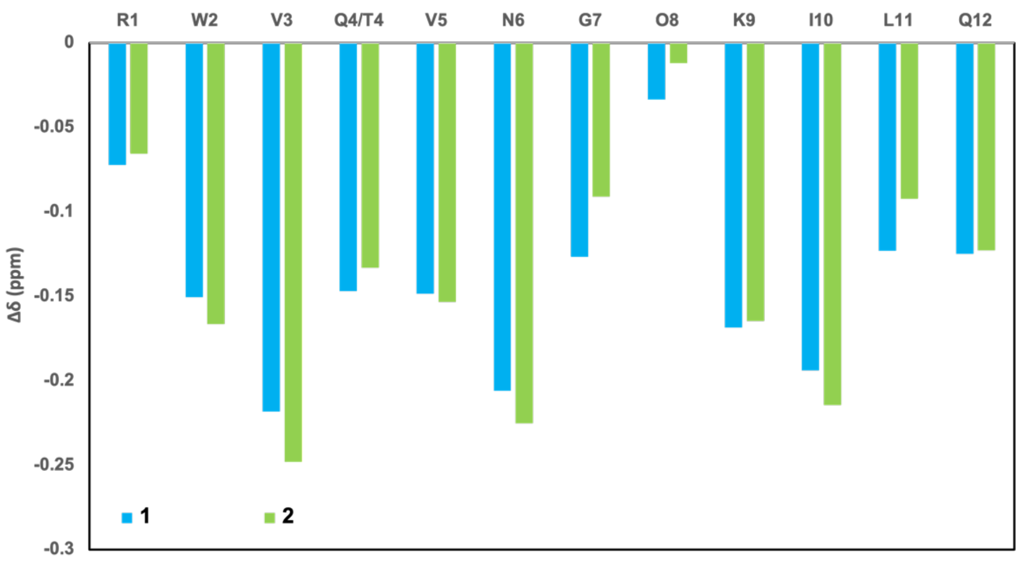

Figure S45. *Referenced amide proton signal shift changes (Δδ, ppm) of the four peptides in the presence of 230 mM $NaReO_4$. The 4th residue was glutamine for **1**, threonine for **2***

4.2. Free energy of unfolding

The stability of a β-hairpin can be described as F ⇌ U, where F and U are respectively the folded and unfolded form of the hairpin. This equilibrium has a K_{eq} = [U]/[F] = (1-f)/f; in which f is the fraction folded (see description in Section 2 above) of that peptide derived from the G7 splitting. Free energies of unfolding are lowered by anions, and this change can be calculated as:

$$\Delta\Delta G^{\circ}_{U,298K} = \Delta G^{\circ}_{U,298K,x\ eq} - \Delta G^{\circ}_{U,298K,0\ eq} = RTln\frac{K_{eq}}{K'_{eq}} = RTln\frac{(1-f)\times f'}{(1-f')\times f}$$

where $\Delta G^{\circ}_{U,298K,x\ eq}$ and $\Delta G^{\circ}_{U,298K,0\ eq}$ are the free energy of unfolding at x eq (x = 48 or 160) and 0 eq (free peptides) of anions; K_{eq} and K'_{eq} are the equilibrium constants at x eq and 0 eq anions; f and f' are fractions folded at x eq and 0 eq anions. Table S18 shows free energy of unfolding change $\Delta\Delta G^{\circ}_{U,298K}$ of four peptides at two concentrations (230 mM and 650 mM) anions (Cl^-, ClO_4^-, and ReO_4^-).

Table S14. *Free energy of unfolding change ($\Delta\Delta G^{\circ}_{U,298K}$, kJ/mol) induced by anions. Errors are ± 0.10 kJ/mol.*

Peptide	48 eq (230 mM) of anions		160 eq (650 mM) of anions	
	**1**	**2**	**1**	**2**
NaCl	-0.17	-0.48	-0.25	-0.78
$NaClO_4$	-0.19	-0.77	-0.44	-1.42
$NaReO_4$	-0.74	-1.44	-1.48	-2.57

4.3. Anion affinity determination

Fitting amide proton shifts to 1:1 binding model (equation 3) was performed as reported.[11] All fitting was performed using MATLAB and was based on the following standard 1:1 model:

$$\Delta\delta_{obs} = \frac{\Delta\delta_{max}}{\frac{2}{K_a G_t - K_a H_t - 1 + \sqrt{(1 - K_a G_t - K_a H_t)^2 + 4 K_a H_t}} + 1} \qquad \textbf{(3)}$$

where $\Delta\delta_{obs}$ (ppm) and $\Delta\delta_{max}$ (ppm) are respectively observed and maximum amide proton shifts, K_a (M^{-1}) is the binding constant, H_t and G_t are respectively host and guest concentrations. Table S19 summarize the obtained binding constant K_a between host (peptide **1**) with guests (Cl^-, I^-, ClO_4^-, ReO_4^-).

Table S15. *Binding constant (K_a, M^{-1}) of host **1** and guest (anions). Errors were ± 15%, which were obtained by triplication.*

	R1	W2	V3	Q4	V5	N6	G7	O8	K9	I10	L11	Q12
NaCl	0.37	1.19	3.36	1.65	2.31	2.29	2.28	- ¶	1.47	3.04	0.73	1.87
NaI	1.13	1.23	1.57	1.50	1.22	1.26	1.36	1.27	1.66	1.37	1.15	0.97
$NaClO_4$	1.19	1.39	1.03	1.65	1.06	0.98	1.03	1.99	1.70	1.79	1.19	0.91
$NaReO_4$	1.56	2.26	1.61	2.46	1.51	1.56	1.45	2.26	2.42	1.44	1.88	1.39

¶ Unfittable.

5. Hydrogen-deuterium exchange (HDX) experiments

A 50 mM peptide stock solution was prepared for the hydrogen-deuterium exchange (HDX) experiments. HDX data collection utilized a 700 MHz Bruker spectrometer, maintaining a constant temperature of 298 K. The peptide stock solutions were diluted in D_2O buffered with 50 mM D_3PO_4 with a pD of approximately 2.3 and loaded into NMR tubes, with and without 230 mM of one of three salts NaCl, $NaClO_4$, $NaReO_4$. Acquisition of 1D proton NMR spectra involved 16 scans, a relaxation delay of 1 second, and an acquisition time of 1 minute for each run. To monitor HDX progress, a series of 1D proton NMR spectra were acquired over 30 minutes to 1 hour. Spectra were processed and analyzed to track changes in peak attributes, revealing deuteration levels at different peptide sites. Peak integrations provided quantification of H/D exchange at specific residues. The NMR spectra exhibited gradual changes in peak features, indicating evolving deuteration levels during the experiment. Degree of deuteration was estimated by comparing peak integrations between exchanged and non-exchanged states, offering insights into solvent accessibility and peptide dynamics. Additionally, a rate constant was determined by fitting peak integrations into a first-order reaction model, $N = N_0 \cdot e^{-kt}$. Each experiment was triplicated, and the reported rate constants were averaged (with errors, standard deviations).

5.1. Without salts

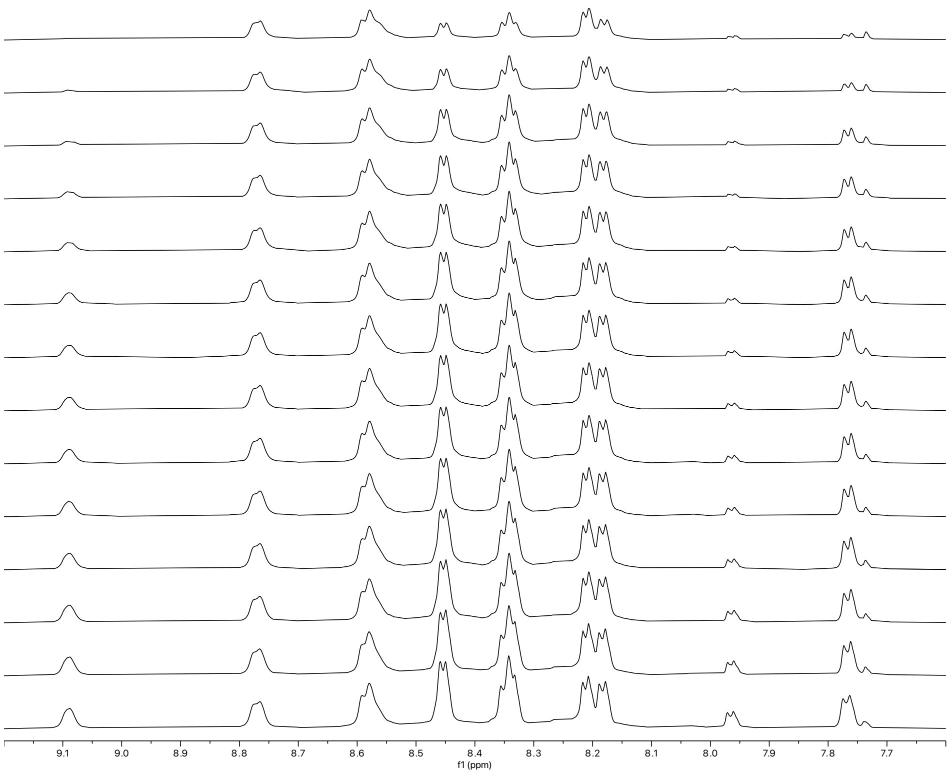

Figure S46. *Stacked NMR showing the amide proton signals of peptide **1** exchanging with deuterium at 298K*

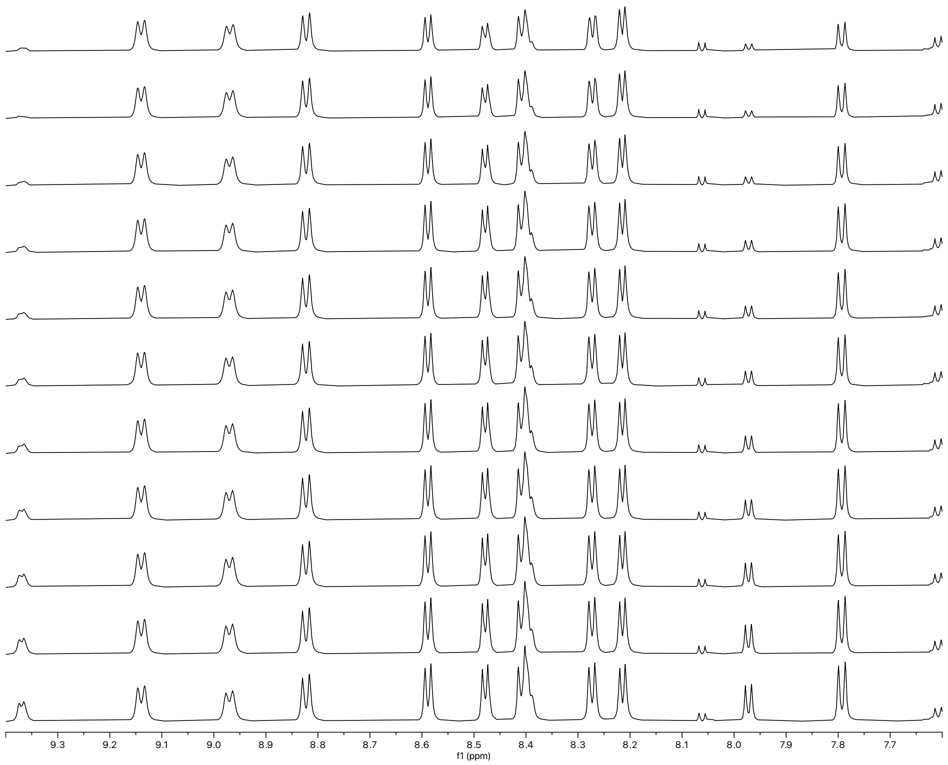

Figure S47. *Stacked NMR showing the amide proton signals of peptide* ***2*** *exchanging with deuterium at 298K*

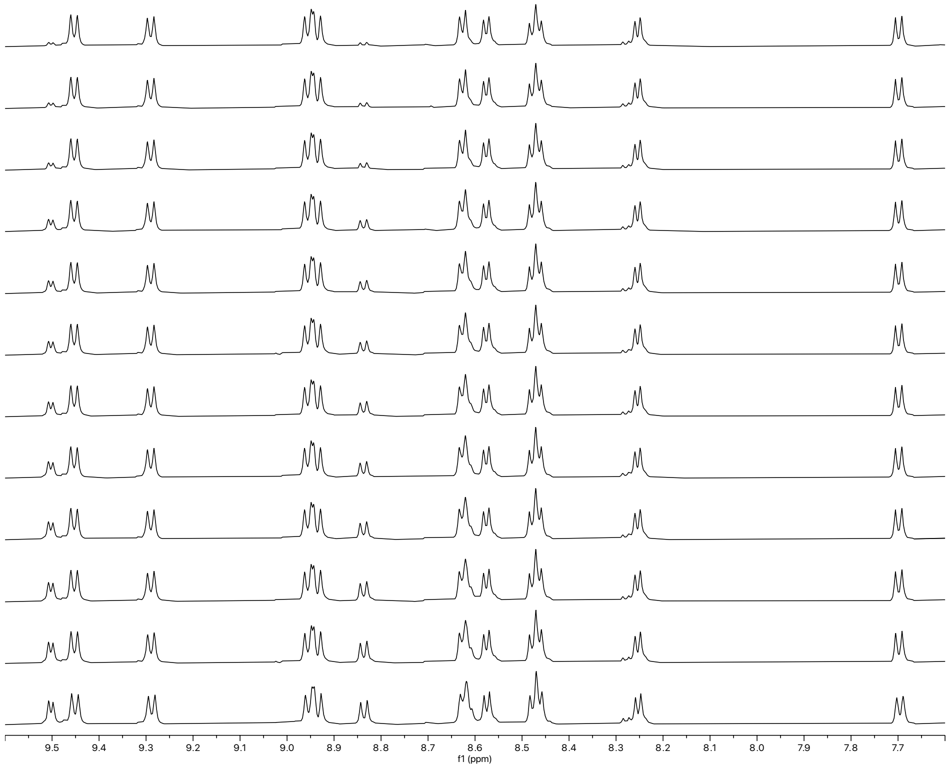

Figure S48. *Stacked NMR showing the amide proton signals of peptide* ***cyc-1*** *exchanging with deuterium at 298K*

5.2. With salts

Exchange rate of **2**'s amide protons were measured in the presence of 230 mM salts with the procedure described above. In peptide **2** with $NaClO_4$ dataset, W2 and L11 exchange rate were averaged since they have the same chemical shifts.

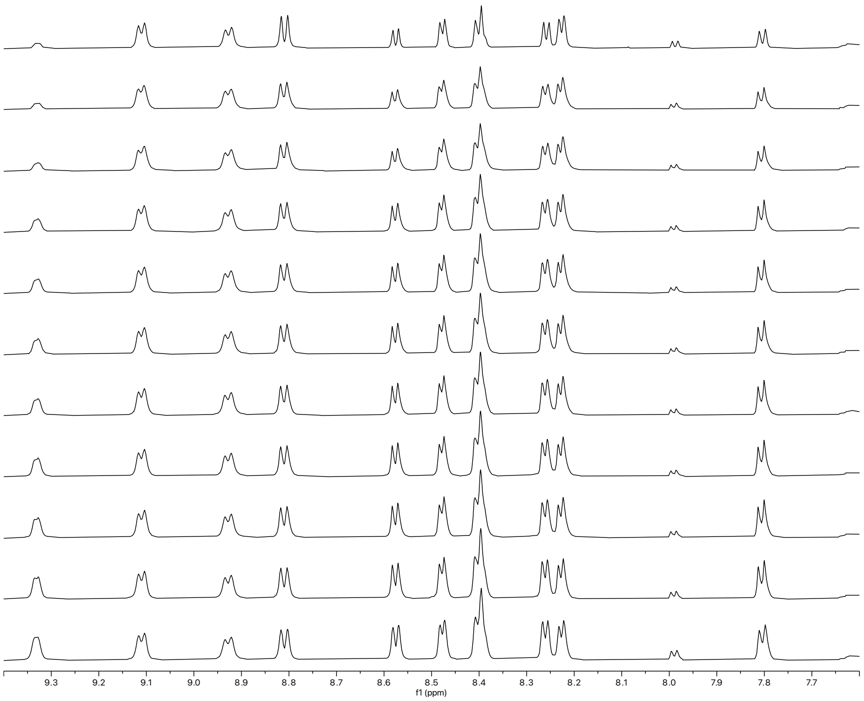

Figure S49. *Stacked NMR showing the amide proton signals of peptide* ***2*** *exchanging with deuterium in the presence of 230 mM NaCl at 298K.*

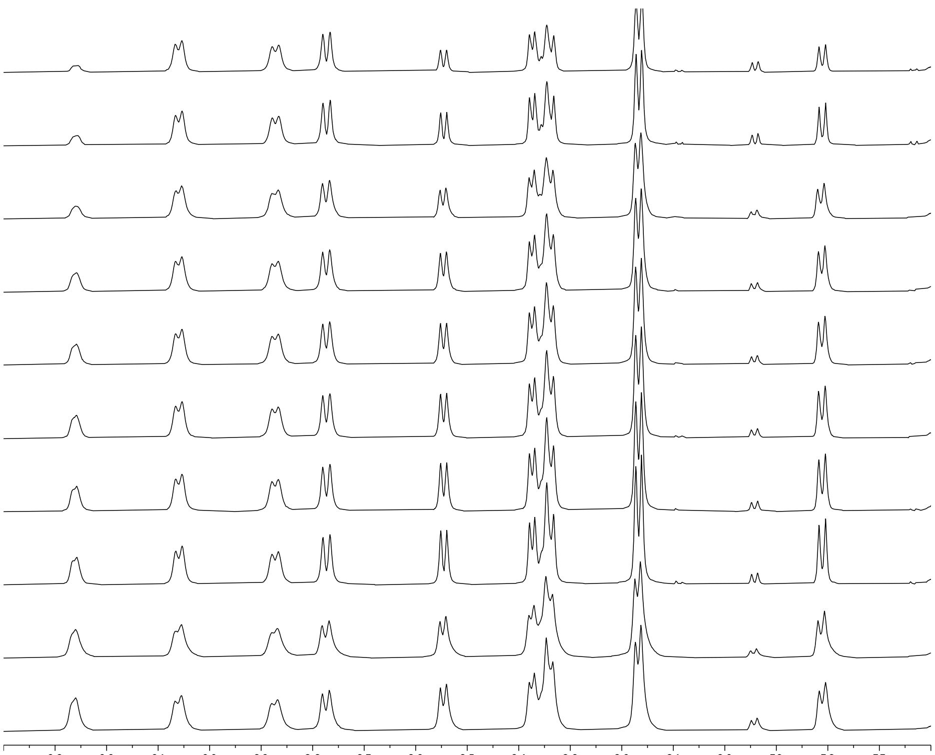

Figure S50. *Stacked NMR showing the amide proton signals of peptide **2** exchanging with deuterium in the presence of 230 mM $NaClO_4$ at 298K.*

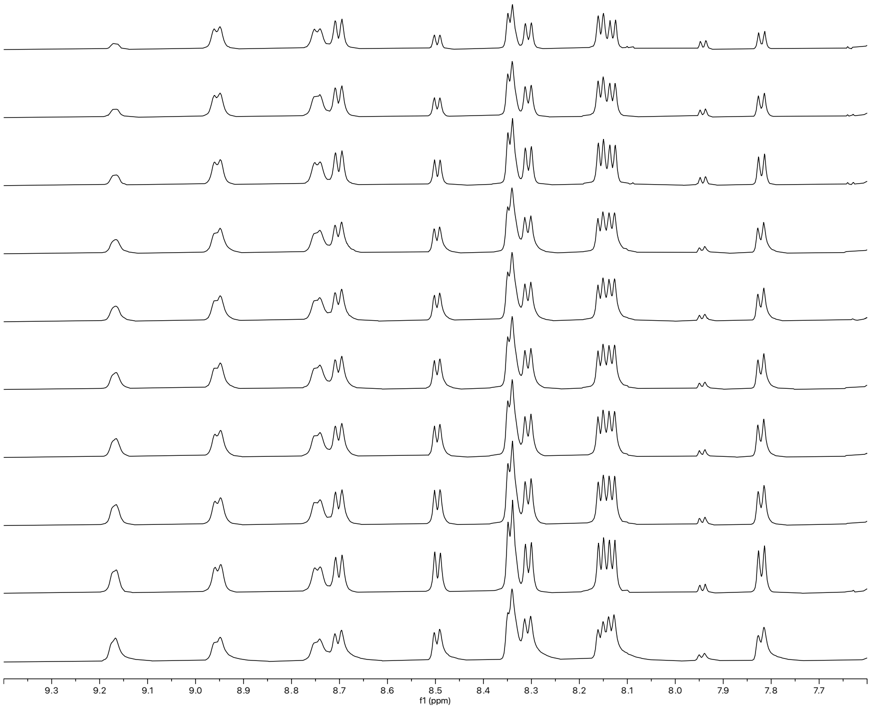

Figure S51. *Stacked NMR showing the amide proton signals of peptide **2** exchanging with deuterium in the presence of 230 mM $NaReO_4$ at 298K.*

6. Anion binding to Cyc-2: H_α and side chain analysis

The backbone amide protons of a protein or peptide serve as informative indicators of anion binding, as illustrated above. However, anions also engage in interactions with additional hydrogen bond donors, including side chain amide and ammonium groups, and, in specific instances, aliphatic protons (H_α, H_β, H_γ, etc.). Given that anions can concurrently bind to and induce the unfolding of the hairpin structure, resulting in a global shift in proton signals, discerning the specific influence of either phenomenon (binding or unfolding) on the chemical shift values becomes challenging. This ambiguity underscores the significance of employing cyclic peptides in this context, as their rigid backbones resist unfolding. Analyzing the chemical shift variations in sidechains of cyclic hairpin peptides in the presence of anions may provide valuable insights into the role of these side chains in the unfolding dynamics of β-hairpin peptides.

Peptides **2**, **cyc-2** and the anion perchlorate were chosen for analysis. Assuming that anions do not have any interactions with the *N*-terminal acetyl group, all spectra were referenced to this signal. Chemical shift changes, Δδ ($\Delta\delta = \delta_{\text{with anion}} - \delta_{\text{no anion}}$), were determined. All $\Delta\delta < 0.005$ ppm (the error) were ignored. Raw tabulated NMR Δδ data were calculated can be found below. The presented data indicates a pronounced affinity of charge-diffused anions, exemplified by perchlorate, for hydrogen bond donors and the hydrophobic core within the hairpin structure. The interaction with the hydrophobic core notably mitigates the hydrophobic effects, thereby contributing to the unfolding of the hairpin structure.

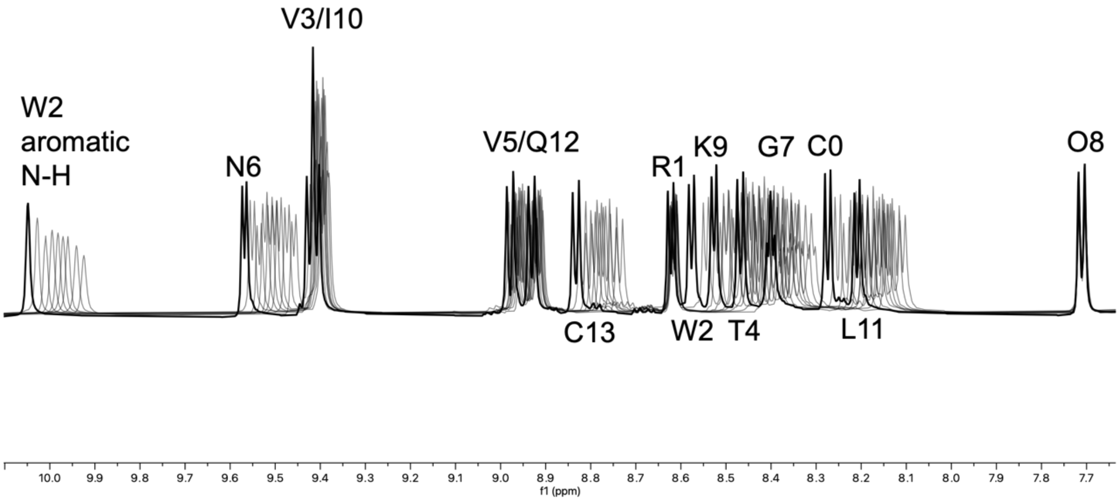

Figure S52. *Superimposed spectra of **cyc-2** showing backbone amide proton and aromatic N-H signal(s) in the presence of increasing concentration of $NaClO_4$ (to 230 mM). The bold data corresponds to zero (0) salt.*

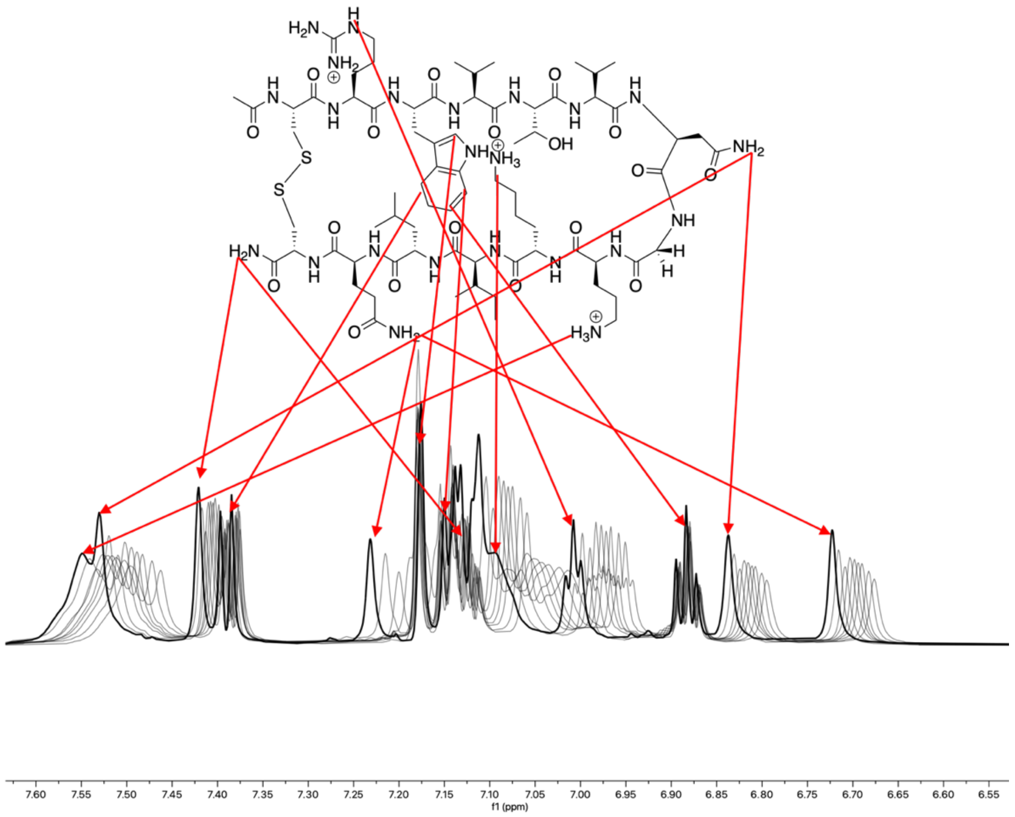

Figure S53. *Superimposed spectra of* ***cyc-2*** *showing side chain amide proton, ammonium proton, and aromatic proton signal(s) in the presence of increasing concentration of* $NaClO_4$ *(to 230 mM). The bold data corresponds to zero (0) salt.*

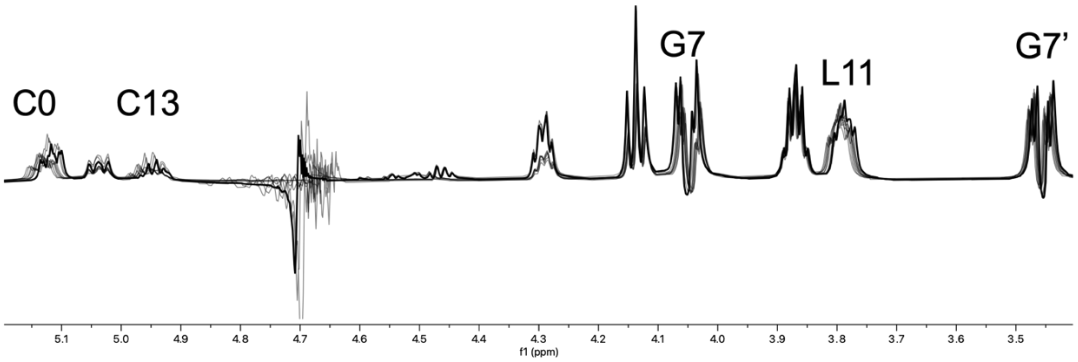

Figure S54. *Superimposed spectra of* ***cyc-2*** *showing backbone* H_α *signal(s) in the presence of increasing concentration of* $NaClO_4$ *(to 230 mM). The bold data corresponds to zero (0) salt. Only signals with* $\Delta\delta > 0.005$ *ppm are labelled.*

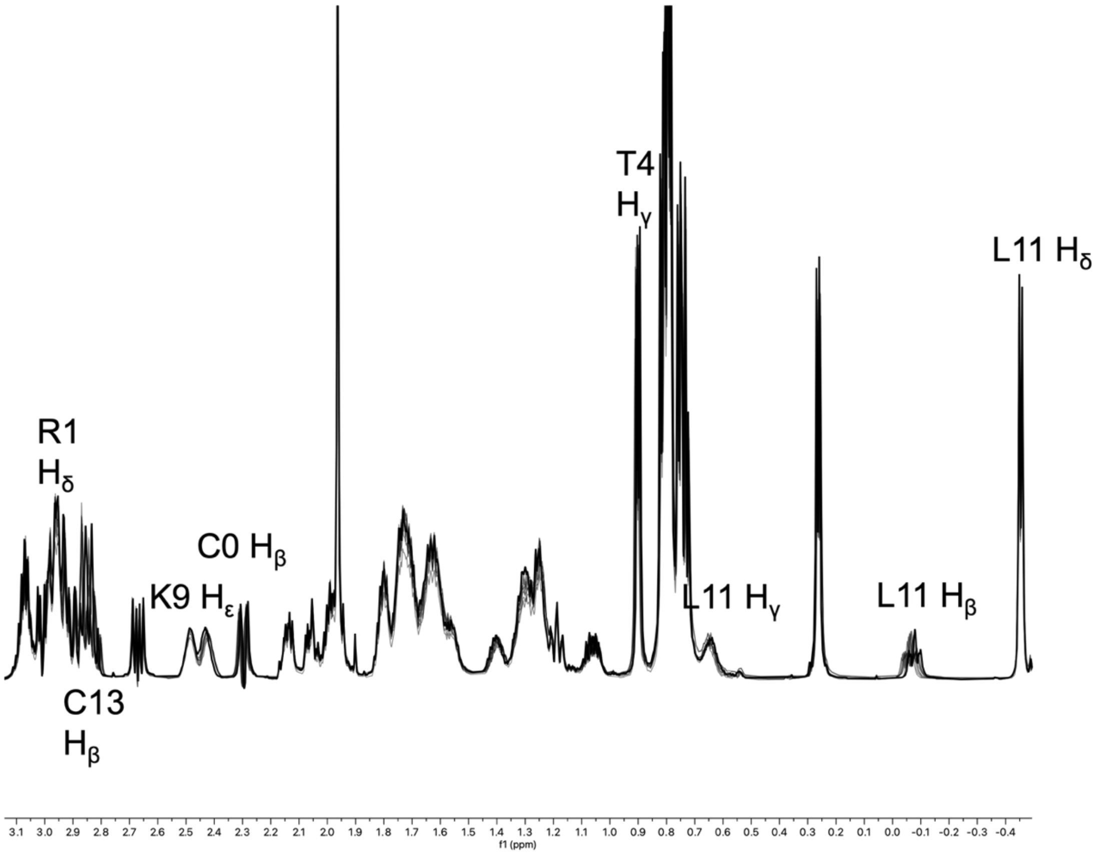

Figure S55. *Superimposed spectra of* ***cyc-2*** *showing side chain aliphatic proton signal(s) in the presence of increasing concentration of* $NaClO_4$ *(to 230 mM). The bold data corresponds to zero (0) salt. Suppressed water peaks are around 4.70 ppm. Only signals with* $\Delta\delta > 0.005$ *ppm are labelled.*

Table S16. *Chemical shift change (Δδ, ppm) for every* $\Delta\delta > 0.005$ *ppm of* ***cyc-2*** *in the presence of 230 mM* $NaClO_4$

Residue/ Group	Amide N–H	H_α	H_β	H_γ	H_δ	H_ε	Side chain N–H
C0	-0.130	0.022	0.008				
R1	-0.009				-0.007		-0.057
W2	-0.164			0.005	-0.011,0.006	-0.007,-0.009	-0.126
V3	-0.035						
T4	-0.121			0.009			
V5	-0.026						
N6	-0.108						-0.068,-0.042
G7	-0.091	-0.009, 0.009					
O8							-0.055
K9	-0.147					0.007	-0.079
I10	-0.017						
L11	-0.099		0.028	0.013	0.006		
Q12	-0.017						-0.097,-0.048
C13	-0.099	0.033	-0.011				
C-Amide							-0.053,-0.025

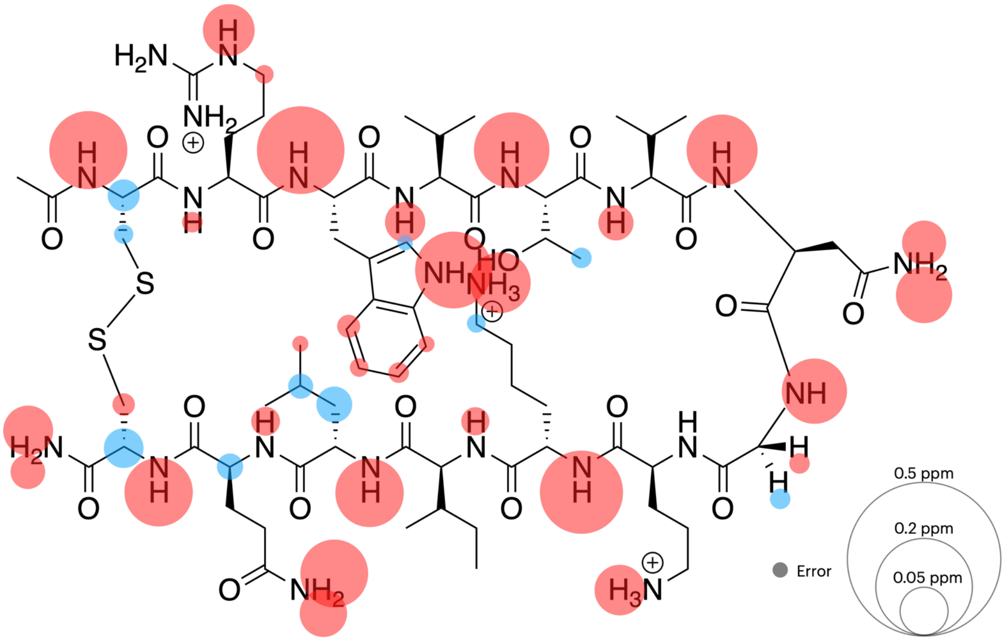

Figure S56. *Representation of Δδ values on* ***cyc-2*** *structure in the presence of 230 mM $NaClO_4$. The circles represent Δδ value with area proportionate to the absolute magnitude of Δδ. Negative values are coded in red, and positive values are coded in blue. Error and reference scale are represented at the bottom right. All HB donors have an upfield shift, suggesting N–H···X^- binding motif. Downfield shifts were observed around indole ring of W2 suggesting the weakening of alkyl-π and cation-π interactions. Significant downfield shifts were also observed at the strained termini.*

7. Molecular dynamics simulations and spatial distribution functions (SDFs)

A sequence of molecular dynamics simulations was executed employing the GROMACS 2016.3 software package to analyze the behavior of the peptide **1**. These simulations were conducted within a bulk water environment at a temperature of 25 °C and a pressure of 1 bar. The peptides were modelled using the Amber-ff03 all-atom force field,[12, 13] while the chloride, iodide, and perchlorate ions were represented using the generalized Amber force field (GAFF). The ion partial charges were derived from AM1-BCC calculations. Water molecules were described using the TIP4P-Ew model.

Peptides containing basic amino acids (lysine, ornithine, or arginine) were assigned a +1 charge for each residue to replicate their protonation state at a pH of 2.3. Each simulation encompassed a single peptide molecule along with 33 anions immersed in a solution containing 3000 water molecules. Constraints were applied to all non-hydrogen atoms of the peptide.

The simulations were conducted under isothermal-isobaric conditions, maintaining temperature using the Nosé-Hoover thermostat and pressure using the Parrinello-Rahman barostat. The equations of motion were numerically integrated using a leapfrog algorithm with a time step of 2 femtoseconds. Electrostatic interactions were computed using the particle mesh Ewald summation method with a real space cutoff of 9 Å. The simulations were run for a duration of 200 nanoseconds in a cubic simulation box with periodic boundary conditions.

The interaction potentials between the anions (A) and nitrogen atoms (N) were defined by the equation:

$$\psi(r_A,r_N)= \frac{q_A q_N}{r_{AN}} + 4\varepsilon_{AN}\left[\left(\frac{\sigma_{AN}}{r_{AN}}\right)^{12} - \left(\frac{\sigma_{AN}}{r_{AN}}\right)^{6}\right]$$

Here, the off-diagonal Lennard-Jones parameter σ_{AN} and the energy term ε_{AN} were determined using the combination rules:

$$\sigma_{AN}=\frac{(\sigma_A+\sigma_N)}{2}$$

$$\varepsilon_{AN}=\sqrt{\varepsilon_A\varepsilon_N}$$

Subsequently, the trajectories generated from these simulations were extracted and transformed into spatial distribution functions using the TRAVIS software[14]. Visualization of these results was carried out using ChimeraX.[15]

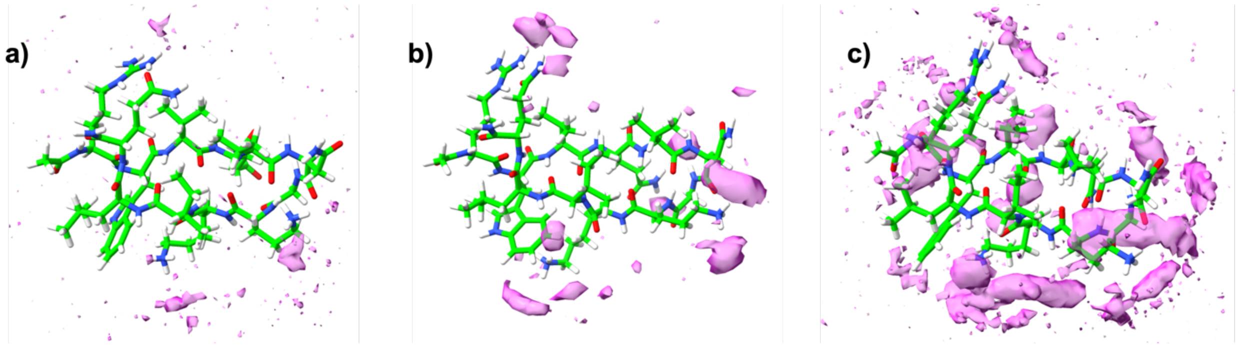

Figure S57. *Spatial distribution function of a) Cl⁻ b) I⁻ c) ClO_4^- with peptide **1**. All densities are at 20× the bulk density.*

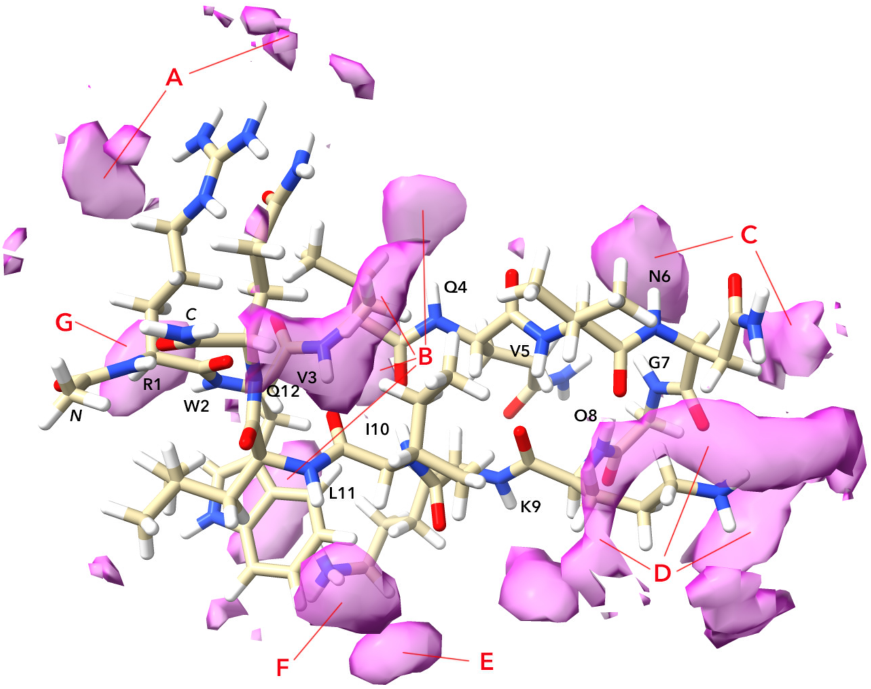

Figure S58. *Perclorate association to **1** as revealed by MD simulations and trajectory analysis (TRAVIS). The peptide mainchain amide groups and termini are labeled (black). The anionic "clouds" (magenta) represent spatial distribution of the anion as defined by probability thresholds set to 40× bulk ion density of $ClO_4^–$. Indicated with the labels A-G (red) are from the top left and clockwise: A) Anion association to the edge and face of the R1 guanidinium group; B) A belt of anion association in the groove between the sidechains of W2 and K9 (lowest highlighted cloud, and center, largely hidden), binding between the sidechains of I10 and Q12 (center, at front), and anion association to the Q4 mainchain amide N–H (top); C) Anion association to the mainchain amide N–Hs of N6 and G7, and the sidechain amide of Q4 (left cloud), and the N6 C_α–H (right cloud); D) A "C" of anion density around the O8 ammonium and between the I10 sidechain (left section) the V5 sidechain (upper left section), and the δ-methylene of the O8 sidechain (right section); E) and F), anion density associated with respectively the K9 and L11 amide N–Hs, and; G) anion association with the W2 amide N–H.*

Figure S58 shows, as magenta "clouds", anion accumulation to **1** corresponding to probability thresholds of 40× bulk $ClO_4^–$ density. The modelling suggests that the largest areas of interaction involve: A) the edge and face of the R1 guanidinium group; B) the grooves between the sidechains of W2 and K9 (*lowest* highlighted cloud, and *center*, largely hidden) and I10 and Q12 (*center*, at front), and anion association to the Q4 mainchain amide N–H (*top*); C) Anion association to the mainchain amide N–Hs of N6 and G7, and the sidechain amide of Q4 (*left* cloud), and the N6 C_α–H (*right* cloud); D) A "C" of anion density around the O8 ammonium and between the I10 sidechain (*left* section) the V5 sidechain (*upper left* section), and the δ-methylene of the O8 sidechain (*right* section); E) and F), anion density associated with respectively the K9 and L11 amide N–Hs, and; G) anion association with the W2 amide N–H. In short, the modeling highlights not only direct association with cationic groups, but also binding into the grooves between packed non-polar groups and select, exposed amide N–H groups. This model suggests that anion binding to the turn is prevalent, involving all the free hydrogen bond donors of N6 and G7 as well as the sidechain amide of Q4. However, the fixed conformation of the peptide means that anion chelation in the terminal region, and anion intercalation into the cation-π-hydrophobic core, is not possible. Thus, the modelling confirms the propensity of charge diffuse anions to bind into non-polar regions of these peptides but cannot provide a picture of induced-fit changes.

References

1. Hughes, R. M.; Benshoff, M. L.; Waters, M. L., Effects of chain length and N-methylation on a cation-pi interaction in a beta-hairpin peptide. *Chemistry* **2007,** *13* (20), 5753-64.
2. Albanese, K. I.; Leaver-Fay, A.; Treacy, J. W.; Park, R.; Houk, K. N.; Kuhlman, B.; Waters, M. L., Comparative Analysis of Sulfonium−π, Ammonium−π, and Sulfur−π Interactions and Relevance to SAM-Dependent Methyltransferases. *Journal of the American Chemical Society* **2022,** *144* (6), 2535-2545.
3. Taylor, J. R.; Thompson, W., *An introduction to error analysis: the study of uncertainties in physical measurements*. Springer: 1982; Vol. 2.
4. Wüthrich, K., NMR with Proteins and Nucleic Acids. *Europhys. News* **1986,** *17* (1), 11-13.
5. Maynard, A. J.; Sharman, G. J.; Searle, M. S., Origin of β-Hairpin Stability in Solution: Structural and Thermodynamic Analysis of the Folding of a Model Peptide Supports Hydrophobic Stabilization in Water. *Journal of the American Chemical Society* **1998,** *120* (9), 1996-2007.
6. Mielke, S. P.; Krishnan, V. V., Characterization of protein secondary structure from NMR chemical shifts. *Prog Nucl Magn Reson Spectrosc* **2009,** *54* (3-4), 141-165.
7. Wishart, D. S.; Sykes, B. D.; Richards, F. M., The chemical shift index: a fast and simple method for the assignment of protein secondary structure through NMR spectroscopy. *Biochemistry* **1992,** *31* (6), 1647-1651.
8. Jumper, J.; Evans, R.; Pritzel, A.; Green, T.; Figurnov, M.; Ronneberger, O.; Tunyasuvunakool, K.; Bates, R.; Žídek, A.; Potapenko, A.; Bridgland, A.; Meyer, C.; Kohl, S. A. A.; Ballard, A. J.; Cowie, A.; Romera-Paredes, B.; Nikolov, S.; Jain, R.; Adler, J.; Back, T.; Petersen, S.; Reiman, D.; Clancy, E.; Zielinski, M.; Steinegger, M.; Pacholska, M.; Berghammer, T.; Bodenstein, S.; Silver, D.; Vinyals, O.; Senior, A. W.; Kavukcuoglu, K.; Kohli, P.; Hassabis, D., Highly accurate protein structure prediction with AlphaFold. *Nature* **2021,** *596* (7873), 583-589.
9. Mirdita, M.; Schütze, K.; Moriwaki, Y.; Heo, L.; Ovchinnikov, S.; Steinegger, M., ColabFold: making protein folding accessible to all. *Nat. Methods* **2022,** *19* (6), 679-682.
10. Davis, M. R.; Dougherty, D. A., Cation-π interactions: computational analyses of the aromatic box motif and the fluorination strategy for experimental evaluation. *Phys Chem Chem Phys* **2015,** *17* (43), 29262-29270.
11. Gibb, C. L. D.; Tran, T. H.; Gibb, B. C., Assessing Weak Anion Binding to Small Peptides. *The Journal of Physical Chemistry B* **2024,** *128* (15), 3605-3613.
12. Hornak, V.; Abel, R.; Okur, A.; Strockbine, B.; Roitberg, A.; Simmerling, C., Comparison of multiple Amber force fields and development of improved protein backbone parameters. *Proteins* **2006,** *65* (3), 712-725.
13. Martín-García, F.; Papaleo, E.; Gomez-Puertas, P.; Boomsma, W.; Lindorff-Larsen, K., Comparing Molecular Dynamics Force Fields in the Essential Subspace. *PLOS ONE* **2015,** *10* (3), e0121114.
14. Brehm, M.; Thomas, M.; Gehrke, S.; Kirchner, B., TRAVIS—A free analyzer for trajectories from molecular simulation. *The Journal of Chemical Physics* **2020,** *152* (16), 164105.
15. Pettersen, E. F.; Goddard, T. D.; Huang, C. C.; Meng, E. C.; Couch, G. S.; Croll, T. I.; Morris, J. H.; Ferrin, T. E., UCSF ChimeraX: Structure visualization for researchers, educators, and developers. *Protein Sci* **2021,** *30* (1), 70-82.
